# Supplementary material for: A comparative analysis of important public clinical trial registries, and a proposal for an interim ideal one
Source: PLoS One. 2021 May 11;16(5):e0251191. doi: 10.1371/journal.pone.0251191 (PMC8112656; doi:10.1371/journal.pone.0251191)
Supplement: Attachment — Submitted filename: Reviewer_PONE-D-20-16268_reviewer (1).pdf [file pone.0251191.s006.pdf]

PLOS ONE

A comparative analysis of important public clinical trial registries, and a proposal for an interim ideal one

--Manuscript Draft--

**Manuscript Number:**	PONE-D-20-16268
**Article Type:**	Research Article
**Full Title:**	A comparative analysis of important public clinical trial registries, and a proposal for an interim ideal one
**Short Title:**	Important public clinical trial registries
**Corresponding Author:**	Gayatri Saberwal, Ph.D.<br>Institute of Bioinformatics and Applied Biotechnology<br>Bangalore, Karnataka INDIA
**Keywords:**	Clinical trial registry; Primary registry; ICTRP; ClinicalTrials.gov; Ethics; Regulatory issues
**Abstract:**	Background<br>It is an ethical and scientific obligation to register each clinical trial, and report its results, accurately, comprehensively and on time. The WHO recognizes 17 public registries as Primary Registries. It has introduced a set of minimal standards (International Standards for Clinical Trial Registries, or ISCTR) that these registries need to implement. This study compared these primary registries, and the US's ClinicalTrials.gov, to examine the implementation of ISCTR, with the aim of defining features of an interim ideal registry.<br><br>Methods and Findings<br>The websites of the 18 registries were evaluated for 17 features that map to one or more of the nine sections of ISCTR, and assigned scores for their versions of 14 of these features. Overall, the registries received between 27% and 80% of the maximum score of 94. The results from our analysis were used to define a set of features of an interim ideal registry. These include the number and nature of fields to conduct a search; data download formats; the nature of the audit trail; and the health condition category. The main limitations of the study are that (i) it does not assess all of the recommendations of the ISCTR, and (ii) although applied systematically, the absolute values of the scores are arbitrary.<br><br>Conclusions<br>To the best of our knowledge, this is the first study to quantify the widely divergent quality of the primary registries' compliance with ISCTR. Even with this limited assessment, it is clear that some of the registries have much work to do, although a mere dozen improvements would significantly improve them. Our study would be helpful to researchers who may wish to extend this audit and evaluate the completeness of the records or the quality of their data, two other major issues, in all 18 registries.
**Order of Authors:**	Nisha Venugopal
	Gayatri Saberwal, Ph.D.
**Additional Information:**	
**Question**	**Response**
**Financial Disclosure**<br><br>Enter a financial disclosure statement that describes the sources of funding for the work included in this submission. Review the submission guidelines for detailed requirements. View published research	GS received internal institutional funds. These were partially from the Government of Karnataka's Department of Information Technology, Biotechnology and Science & Technology (https://itbtst.karnataka.gov.in/english). There was no grant number.<br><br>The funders had no role in study design, data collection and analysis, decision to publish, or preparation of the manuscript.

Powered by Editorial Manager® and ProduXion Manager® from Aries Systems Corporation

articles from *PLOS ONE* for specific examples.<br><br>This statement is required for submission and **will appear in the published article** if the submission is accepted. Please make sure it is accurate.<br><br>**Unfunded studies**<br>Enter: *The author(s) received no specific funding for this work.*<br><br>**Funded studies**<br>Enter a statement with the following details:<br>• Initials of the authors who received each award<br>• Grant numbers awarded to each author<br>• The full name of each funder<br>• URL of each funder website<br>• Did the sponsors or funders play any role in the study design, data collection and analysis, decision to publish, or preparation of the manuscript?<br>• **NO** - Include this sentence at the end of your statement: *The funders had no role in study design, data collection and analysis, decision to publish, or preparation of the manuscript.*<br>• **YES** - Specify the role(s) played.<br><br>* typeset	
**Competing Interests**<br><br>Use the instructions below to enter a competing interest statement for this submission. On behalf of all authors, disclose any competing interests that could be perceived to bias this work—acknowledging all financial support and any other relevant financial or non-financial competing interests.<br><br>This statement **will appear in the published article** if the submission is accepted. Please make sure it is accurate. View published research articles from *PLOS ONE* for specific examples.	The authors have declared that no competing interests exist.

**NO authors have competing interests**<br><br>Enter: *The authors have declared that no competing interests exist.*<br><br>**Authors with competing interests**<br><br>Enter competing interest details beginning with this statement:<br><br>*I have read the journal's policy and the authors of this manuscript have the following competing interests: [insert competing interests here]*<br><br>* typeset	
**Ethics Statement**<br><br>Enter an ethics statement for this submission. This statement is required if the study involved:<br><br>• Human participants<br>• Human specimens or tissue<br>• Vertebrate animals or cephalopods<br>• Vertebrate embryos or tissues<br>• Field research<br><br>Write "N/A" if the submission does not require an ethics statement.<br><br>General guidance is provided below. Consult the submission guidelines for detailed instructions. **Make sure that all information entered here is included in the Methods section of the manuscript.**	Not applicable

Powered by Editorial Manager® and ProduXion Manager® from Aries Systems Corporation

**Format for specific study types**<br><br>**Human Subject Research (involving human participants and/or tissue)**<br>• Give the name of the institutional review board or ethics committee that approved the study<br>• Include the approval number and/or a statement indicating approval of this research<br>• Indicate the form of consent obtained (written/oral) or the reason that consent was not obtained (e.g. the data were analyzed anonymously)<br><br>**Animal Research (involving vertebrate animals, embryos or tissues)**<br>• Provide the name of the Institutional Animal Care and Use Committee (IACUC) or other relevant ethics board that reviewed the study protocol, and indicate whether they approved this research or granted a formal waiver of ethical approval<br>• Include an approval number if one was obtained<br>• If the study involved *non-human primates*, add *additional details* about animal welfare and steps taken to ameliorate suffering<br>• If anesthesia, euthanasia, or any kind of animal sacrifice is part of the study, include briefly which substances and/or methods were applied<br><br>**Field Research**<br><br>Include the following details if this study involves the collection of plant, animal, or other materials from a natural setting:<br>• Field permit number<br>• Name of the institution or relevant body that granted permission	
**Data Availability**<br><br>Authors are required to make all data underlying the findings described fully available, without restriction, and from the time of publication. PLOS allows rare exceptions to address legal and ethical concerns. See the PLOS Data Policy and FAQ for detailed information.	Yes - all data are fully available without restriction

A Data Availability Statement describing where the data can be found is required at submission. Your answers to this question constitute the Data Availability Statement and **will be published in the article**, if accepted.<br><br>**Important:** Stating 'data available on request from the author' is not sufficient. If your data are only available upon request, select 'No' for the first question and explain your exceptional situation in the text box.<br><br>Do the authors confirm that all data underlying the findings described in their manuscript are fully available without restriction?	
**Describe where the data may be found in full sentences. If you are copying our sample text, replace any instances of XXX with the appropriate details.**<br><br>• If the data are **held or will be held in a public repository**, include URLs, accession numbers or DOIs. If this information will only be available after acceptance, indicate this by ticking the box below. For example: *All XXX files are available from the XXX database (accession number(s) XXX, XXX.*).<br>• If the data are all contained **within the manuscript and/or Supporting Information files**, enter the following: *All relevant data are within the manuscript and its Supporting Information files.*<br>• If neither of these applies but you are able to provide **details of access elsewhere**, with or without limitations, please do so. For example:<br><br>*Data cannot be shared publicly because of [XXX]. Data are available from the XXX Institutional Data Access / Ethics Committee (contact via XXX) for researchers who meet the criteria for access to confidential data.*<br><br>*The data underlying the results presented in the study are available from (include the name of the third party*	Most relevant data are within the manuscript and its Supporting Information files. Some data is available on the websites of the registries, at URLs that are referenced in the various Supplementary Files, as relevant.

and contact information or URL).<br>• This text is appropriate if the data are owned by a third party and authors do not have permission to share the data.<br><br>* typeset	
Additional data availability information:	

Manuscript Click here to download Manuscript Manuscript.docx

A comparative analysis of important public clinical trial registries, and a proposal for an interim ideal one

Nisha Venugopal and Gayatri Saberwal*

Emails: nisha.venugopal2015@gmail.com, gayatri@ibab.ac.in

Authors' affiliation:
Institute of Bioinformatics and Applied Biotechnology
Biotech Park, Electronics City Phase 1
Bengaluru 560100
Karnataka
India

*Corresponding author:
E-mail: gayatri@ibab.ac.in

Keywords: Clinical trial registry, Primary registry, ICTRP, ClinicalTrials.gov, Ethics, Regulatory issues

Abstract

Background

It is an ethical and scientific obligation to register each clinical trial, and report its results, accurately, comprehensively and on time. The WHO recognizes 17 public registries as Primary Registries. It has introduced a set of minimal standards (International Standards for Clinical Trial Registries, or ISCTR) that these registries need to implement. This study compared these primary registries, and the US's ClinicalTrials.gov, to examine the implementation of ISCTR, with the aim of defining features of an interim ideal registry.

Methods and Findings

The websites of the 18 registries were evaluated for 17 features that map to one or more of the nine sections of ISCTR, and assigned scores for their versions of 14 of these features. Overall, the registries received between 27% and 80% of the maximum score of 94. The results from our analysis were used to define a set of features of an interim ideal registry. These include the number and nature of fields to conduct a search; data download formats; the nature of the audit trail; and the health condition category. The main limitations of the study are that (i) it does not assess all of the recommendations of the ISCTR, and (ii) although applied systematically, the absolute values of the scores are arbitrary.

Conclusions

To the best of our knowledge, this is the first study to quantify the widely divergent quality of the primary registries' compliance with ISCTR. Even with this limited assessment, it is clear that some of the registries have much work to do, although a mere dozen improvements would significantly improve them. Our study would be helpful to researchers

who may wish to extend this audit and evaluate the completeness of the records or the quality of their data, two other major issues, in all 18 registries.

Introduction

The first two calls for clinical trial registries were made in the 1970s [1]. One aimed to enhance the enrollment of patients in trials, and the other to reduce the possibility of bias in the subsequent reporting of trial results, caused by the selective publication of those with positive outcomes. Since the year 2000, trial registries have proliferated. Nevertheless, it has been a long and sustained battle by many stakeholders – activists, journals, researchers, funders, governments and the World Health Organization (WHO) – to ensure that large numbers of trials are registered [2–4]. Although the initial two aims for setting up registries continue to be among the most important uses of such databases, researchers have utilized the data in at least a dozen other ways [5–18]. Given these numerous and diverse purposes, not initially envisaged, it is even more important that all trials are registered and reported in a timely fashion, and that all the data in each record is complete, reliable and readily accessible. In view of this, the quality of data in the databases has long been the subject of analysis and comment [[illegible],19–25].

Set up to facilitate access to clinical trial information around the world, the WHO's International Clinical Trials Registry Platform (ICTRP) initiative recognizes 17 registries as Primary Registries (PRs). Although ClinicalTrials.gov (CTG), of the United States (US), is not one of them, it is the oldest, and by far the largest, public registry, and is considered a data provider to ICTRP. The platform enables a search for trials in all 18 registries [26]. In this study, we have analyzed all of them, and refer to them, collectively, as Primary Registries(+), or PR+.

WHO's International Standards for Clinical Trial Registries (ISCTR) [27] lists the minimum, and sometimes ideal, standards that PRs should adopt to ensure a basic quality of

data and accessibility. These standards are in nine sections. We wished to undertake a comparative assessment of the PR+, to assess their implementation of ISCTR. In order to do so, we have developed the Registries' Comparative Scorecard (the Scorecard) which rates the PR+ on certain features that map to various sections of the ISCTR (S1 Table). We end by defining an interim 'ideal registry' based on the best versions of each feature used by the PR+. Until such time as all the registries adopt all the standards recommended by ISCTR, the adoption of the recommended versions of each feature would be very helpful for users.

Methods

We accessed the websites of the 18 PR+ between July 2019 and April 2020, inclusive. The registries were evaluated for 17 features that map to one or more of the nine sections of ISCTR, that is (i) Content; (ii) Quality and Validity; (iii) Accessibility; (iv) Unambiguous Identification; (v) Technical Capacity; (vi) Administration and Governance; (vii) The WHO's mandated 24-field Trial Registration Data Set (TRDS); (viii) Partner Registries; and (ix) Data Interchange Standards. The list of features was compiled based on literature regarding the necessity of higher quality trial registrations [24,27], focusing on the standards listed in ISCTR [27].

All information was obtained from one or more of the following resources within each PR+ website: (i) the general pages of the site; (ii) a randomly chosen, sample interventional trial that was registered after 1 January 2019; (iii) supporting documents, if available; and (iv) where necessary and possible, via a login to do a mock registration. All analyses were performed by NV and verified by GS, with differences resolved by discussion. Further methodological details and reference URLs are available in the Supplementary files, which are referenced in the Results as relevant.

The Registries' Comparative Scorecard (the Scorecard)

Each registry has a particular variant of a given feature. This variant may be more useful or less so, and we have assigned a score accordingly. The overall scoring system is as follows: (i) if the feature is absent, the registry gets a 0; (ii) for features with multiple variants, the score ranges from 1 to 5; and (iii) for certain features, the score increases by one per field. In case a registry has multiple possible scores for a particular feature, the highest one is awarded.

Results

We first documented basic information about each of the registries, which is in Table 1. Except CTG, the acronyms used for each registry were the official acronyms. On 18 April 2020, the registries cumulatively held 5,72,901 records, with CTG accounting for 59 % of them.

Table 1. An overview of each registry, listing its acronym, full name, country where it is based, year established, number of trial records held, countries from where registration is accepted, type of registration allowed, type of study hosted, and language used.

Registry acronym	Registry full name	Country where registry is based	Year established	Number of records[1]	Countries from where registration is accepted	Type of registration allowed	Type of study	Additional language[2]
ANZCTR	Australian New Zealand Clinical Trials Registry	Australia	2005	19150	All countries. However, trials in Australia and New Zealand are prioritized	Prospective, Retrospective[3]	Interventional, Observational	–
ChiCTR	Chinese Clinical Trial Register	China	2005	31578	All countries	Prospective, Retrospective	Interventional, Observational, Others	Chinese
CRIS	Clinical Research Information Service	Republic of Korea	2010	4916	Republic of Korea	Prospective, Retrospective	Interventional, Observational	Korean
CTG[4]	ClinicalTrials.gov	USA	2000	336444	All countries	Prospective[5]	Interventional, Observational, Expanded Access	–

CTRI	Clinical Trials Registry - India	India	2007	24718	Other countries in the region which do not have a Primary Registry of their own[6]	Prospective	Interventional, Observational, PMS[7], BA/BE[7]	–
DRKS	German Clinical Trials Register	Germany	2008	9581	All countries	Prospective, Retrospective	Interventional, Observational, Epidemiological, Others	German
EU-CTR	EU Clinical Trials Register (EU-CTR)	The Netherlands	2004	36915[8]	All interventional trials that have at least one centre in the EU and EEA. Certain trials conducted entirely outside these regions.	Prospective. Retrospective if permitted by National Competent Authority of the Member State	Interventional	Older trials may have content in the host country's language
IRCT	Iranian Registry of Clinical Trials	Iran	2008	24117	All countries	Prospective	Interventional	Persian
ISRCTN	International Standard Registered Clinical/soCial sTudy Number	UK	2000	19329	All countries	Prospective, Retrospective[3]	Interventional, Observational	–
JPRN	Japan Primary Registries Network[9]	Japan	2008	44873	All countries	Prospective, Retrospective	Interventional, Observational	Japanese
LBCTR	Lebanon Clinical Trials Registry	Lebanon	2019	69	Lebanon	Prospective	Interventional, Observational	Brief summary of the study is also available in Arabic
NTR	Netherlands Trial Register	The Netherlands	2004	8521	Trials conducted in Netherlands or involving Dutch researchers.	Prospective, Ongoing studies	Interventional, Observational	Some information may be available in Dutch
PACTR	Pan African Clinical Trials Registry	South Africa	2009	2318	All countries in Africa	Prospective, Retrospective	Interventional	–
ReBEC	Brazilian Registry of Clinical Trials	Brazil	2010	3992	Brazil[6]	Prospective Retrospective	Interventional, Observational	Portugese and Spanish, for some records, and in a limited way
REPEC	Peruvian Clinical Trial Registry	Peru	2007	1845	Peru	Prospective	Interventional	Spanish

RPCEC	Cuban Public Registry of Clinical Trials	Cuba	2007	307	Cuba[10]	Prospective, Retrospective	Interventional Observational	Spanish
SLCTR	Sri Lanka Clinical Trials Registry	Sri Lanka	2006	345	All countries	Prospective	Interventional	–
TCTR	Thai Clinical Trials Registry	Thailand	2009	3883	Thailand	Prospective	Interventional, Observational	–

As mentioned, we analyzed 17 features of the PR+. However, as explained below, we evaluated only 14 of them to develop the Scorecard, details of which are provided in Table 2a. Here, we list the various features analyzed, and the maximum score possible for the feature, We also provide the score obtained by each PR+ per feature, and overall. To be noted, ISCTR recommends 24 Trial Registration Data Set (TRDS) fields. Any other field was referred to as an Extra field. In Table 2b , we describe the rationale for the scores, and the weightage given to each variant of each feature. Further details are provided below, or are available in relevant Supplementary files, which are referenced in Table 2b. We have grouped the features according to the sections of ISCTR that they mapped to.

Table 2a. The Scorecard. [We would like Table 2b to be part of 2a, on the right, but the uploading system didn't permit it.]
The list of features used to create the Scorecard; the maximum score per feature; the score obtained by each registry per feature, and overall per section; the total score per registry; and the rank of each registry.

		Max score	ANZCTR	ChiCTR	CRIS	CTG	CTRI	DRKS	EU-CTR	IRCT	ISRCTN	JPRN	LBCTR	NTR	PACTR	ReBEC	REPEC	RPCEC	SLCTR	TCTR
**1**	**Accessibility section**																			
1.1	Total number of trials in the registry	**5**	3	3	5	5	3	3	5	5	3	3	1	3	3	5	3	2	2	3
1.2	Existence of Basic search function	**5**	5	0	5	5	5	5	5	5	5	5	0	5	5	5	5	5	0	5
1.3	Advanced search function – TRDS fields	**24**	11	17	14	15	10	8	7	17	13	0	9	0	12	4	1	5	2	7
1.4	Advanced search function – Extra fields	**5**	1	5	5	5	4	5	3	5	5	0	0	0	2	1	2	0	0	0
1.5	Data download options	**5**	5	2	2	5	2	5	2	2	5	5	2	0	2	2	2	2	2	2
	**SUB-TOTAL**	**44**	**25**	**27**	**31**	**35**	**24**	**26**	**22**	**34**	**31**	**13**	**12**	**8**	**24**	**17**	**13**	**14**	**6**	**17**
**2**	**Content or TRDS sections**																			
2.1	Brief view: TRDS fields	**5**	3	3	4	5	3	3	3	3	1	3	3	1	3	3	3	1	1	5
2.2	Brief view: Extra fields	**5**	3	1	2	2	0	3	1	3	1	1	1	0	3	0	0	1	5	1
2.3	Detailed view: TRDS fields	**4**	4	4	3	4	3	2	2	2	4	4	4	3	4	0	3	1	4	3
2.4	Detailed view: Extra fields	**15**	10	5	10	15	8	6	9	7	10	0	9	5	5	4	10	6	5	9
2.5	Whether PI name is compulsory	**5**	5	5	5	0	0	5	5	2	2	2	2	2	5	0	2	5	2	2

2.6	Audit trail	**3**	1	1	3	3	1	3	0	3	2	0	1	0	3	0	0	3	2	0
	**SUB-TOTAL**	**37**	**26**	**19**	**27**	**29**	**15**	**22**	**20**	**20**	**20**	**10**	**20**	**11**	**23**	**7**	**18**	**17**	**19**	**20**
**3**	**Other sections**																			
3.1	Health condition	**5**	5	3	5	3	5	5	5	3	1	5	5	1	5	5	3	1	1	3
3.2	SSL certificate	**5**	5	0	5	5	0	5	5	5	0	5	0	5	5	0	5	0	5	5
3.3	Documentation	**3**	3	2	2	3	3	3	3	1	3	0	3	0	2	3	2	2	2	3
	**SUB-TOTAL**	**13**	**13**	**5**	**12**	**11**	**8**	**13**	**13**	**9**	**4**	**10**	**8**	**6**	**12**	**8**	**10**	**3**	**8**	**11**
	**TOTAL**	**94**	**64**	**51**	**70**	**75**	**47**	**61**	**55**	**63**	**55**	**33**	**40**	**25**	**59**	**32**	**41**	**34**	**33**	**48**
	**% of TOTAL**		**68**	**54**	**74**	**80**	**50**	**65**	**59**	**67**	**59**	**35**	**43**	**27**	**63**	**34**	**44**	**36**	**35**	**51**
	**Rank of each registry**		**3**	**9**	**2**	**1**	**11**	**5**	**7**	**4**	**7**	**15**	**13**	**18**	**6**	**17**	**12**	**14**	**15**	**10**

126

**Table 2b. Rationale for weightage given to each registry for features used to create the Scorecard.**<br>The relevant Supplementary files with further details are also referenced.			
**Feature analyzed**		**Rating scale and rationale**	**Relevant Supplementary file**
**1**	**Accessibility**		
1.1	Total number of trials in the registry	Number displayed on home page: 5<br>Number available after a search: 3<br>Number needs to be calculated: 2<br>Discrepant information at different places on the site: 1	S2 Table
1.2	Existence of Basic search function	Presence of a basic search function: 5<br>Absence of a basic search function: 0	S2 Table
1.3	Advanced search function – TRDS fields	Each TRDS field: 1	S2 Table
1.4	Advanced search function – Extra fields	Each extra field: 1, but with a cap of 5 overall, because of the idiosyncratic nature of some of the search possibilities.	S2 Table
1.5	Data download options	Excel/csv/tsv: 5<br>HTML/XML: 2<br>Word/txt/pdf: 1<br>No download options: 0<br>Since all the registries except NTR permit HTML downloads (even if it is not explicitly stated), no registry gets a rating of '1'.	S2 Table
2	**Content or TRDS sections**		
2.1	Brief view: TRDS fields	10 or more fields, which are customizable, and wrapping of text: 5<br>10 or more fields, which are customizable, but without wrapping of text: 4<br>A fixed number of fields, that are more than 3: 3<br>Upto 3 fields: 1	S3 Table
2.2	Brief view: Extra fields	Each field: 1 point	S4 Table
2.3	Detailed view: TRDS fields	The number of fields over 20	S3 Table
2.4	Detailed view: Extra fields	Each field: 1 point<br>In this case, the maximum score is dictated by the registry with the maximum number of fields.	S4 Table
2.5	Whether PI name is compulsory	PI name is compulsory: 5<br>It is not clear whether the scientific contact is the PI (regardless of whether or not this information is compulsory): 2<br>There is a field for the PI name, but it is not clear whether the information is compulsory: 2<br>The PI name is voluntary: 0	S5 Table
2.6	Audit trail	Each of the following aspects receives 1 point: (i) the existence of an audit trail; (ii) the changes made are clearly highlighted; and (iii) it is possible to compare any two versions of the record.	S5 Table
**3**	**Other Sections**		
3.1	Health condition	A drop-down menu for choosing a term from a controlled vocabulary: 5<br>A widely used controlled vocabulary is recommended: 3<br>Free text field: 1	S5 Table
3.2	SSL certificate	Website secured with SSL: 3<br>Website not secured with SSL: 0	S5 Table
3.3	Documentation	Provides (a) a glossary or the definition of each field of the record; (b) List of FAQs; (c) One or more user guides: 1 point each. No points are awarded for the quality of these documents.	S5 Table

1. Accessibility:

We first examined the accessibility of information in the PR+. For this, we assessed several features, as follows: (a) whether the registry website was live, (b) the time taken to obtain the results of a particular search, (c) the total number of trials in the registry, (d) the existence of a basic search function, (e and f) the existence of an advanced search function with TRDS fields and Extra fields and (g) the data download options. We comment on only two of them here. (a) Whether the registry website was live: During the course of this work, most of the registries were live most of the time. However we formally checked this on three occasions (11 and 25 February, and 10 March 2020). All the websites were up each time, and so we did not score this aspect. (b) Time taken to obtain the results of a search: In our work over several months, we often found that RPCEC loaded search results slowly. However when we formally assessed the time taken to obtain the results of a search with the word ‘cancer’, the average time taken over five successive days was in seconds for each registry. We found this adequate, and so we did not score this aspect either.

2. Content or TRDS sections:

Next, we examine several features that mapped to the Content or TRDS sections, as follows: (a) Brief view – TRDS fields, (b) Brief view – Extra fields, (c) Detailed view – TRDS fields, (d) Detailed view – Extra fields, (e) whether the Principal Investigator (PI) name is compulsory, (f) reason for the termination of a trial, and (g) audit trail. Since almost half (seven) the registries did not have a category of ‘terminated trials’, we did not score the relevant feature.

3. Other sections:

Finally, we examined three features that map to other sections, as follows: (a) Health condition, (b) the presence of a Secure Sockets Layer (SSL) certificate and (c) Documentation.

The maximum score that any registry could obtain is 94 points (Table 2). The PR+ received scores ranging from 27% (NTR) to 80% (CTG) of the maximum, with an average of 52%.

Discussion

As mentioned, the PR+ received scores ranging from 27% to 80% of the maximum score. This derives from an assessment of 14 features, many of which we discuss in Box 1, that largely map to (i) Accessibility, or (ii) Content or TRDS. We have barely touched upon Quality and Validity, since investigating the completeness or quality of the records in the PR+ would be a large exercise in itself. For several of the minimum standards recommended by ISCTR, either it is not possible for us to assess compliance, or the requirements do not immediately impact use of the registry data. Therefore we have also barely touched upon Unambiguous Identification (which Secondary identifying numbers, a field in TRDS, also maps to), Technical Capacity, and Data Interchange Standards. Further, we have not touched upon the sections (i) Administration and Governance, and (ii) Partner Registries. Despite the limited nature of our audit, the lowest- and highest-scoring registries receive scores that differ by over 50%. To the best of our knowledge, this widely divergent quality of the PR+ has not been documented before.

Box 1 Discussion of specific features assessed in the scorecard

Here we discuss the features that map to some of the nine sections of the ISCTR.

We begin with the section Accessibility, a major goal of ISCTR, which we assessed from multiple angles. ISCTR requires that registry data be available to users at all times. During the course of this work, we have found this to be so. The time taken to load the results of a search is also an aspect of accessibility to data, and we found the search times of the PR+ to be largely adequate. However it would require a more detailed audit to evaluate this feature comprehensively, and therefore we have not scored it.

One of the most important reasons for the existence of such registries is to provide the public with information, and to thereby increase trust in the trial enterprise [28] .One of the fundamental pieces of information concerns how many records the database holds. This should be readily accessible, and we have therefore analyzed the ease of accessing this number. For nearly all users, however, the search function is the most crucial part of accessing information in a registry. ISCTR recommends that at the minimum, the registries must allow a basic text search as well as searches within the interventions and conditions fields. Several PR+ go much further than this, and therefore we have conducted a detailed assessment of their search capabilities. Having conducted a search, users may wish to download many fields of data, for many records. All the data download options are adequate for the inspection of a few records, but it is essential that each PR+ provide a csv, excel or tsv format to enable larger scale analyses. It should not be necessary to utilize web scraping tools to retrieve large amounts of data, since that would limit access to users with programming skills.

Next we examined multiple features that map to the Content or TRDS sections, which overlap since the TRDS fields are a form of content. Each registry provides information about a trial in two different ‘views’. After the user conducts a search, there is a list of trials which always contains the title of the trial, but may also contain other information. This is called the Brief view. After

clicking on the title, the Detailed view becomes available, with much more information. It can be very helpful for a user if the number of fields in the Brief view can be customized, and we have therefore given the highest score for this. The Detailed view tends to have all the TRDS fields. However, all the PR+ do not yet list the four fields that have been included in the latest version of TRDS [29]. We can expect more of them to do so over time.

In both the Brief and Detailed views, registries list Extra fields. Some of these, such as last update date, and whether registration was prospective or retrospective, are recommended by ISCTR. Interestingly, one-third or more of the PR+ list several fields that the ISCTR does not specifically recommended. This seems to reflect a certain level of agreement among the registries that particular fields are important.

Among the large number of fields listed by one or another registry, we wish to specifically comment on a few of them. First, the issue of whether the PI name is compulsory. For the sake of accountability, it is important that this be so [23]. ISCTR states that the PI is the 'Contact for Scientific Queries', unless the PI delegates this task to somebody else. Although we assessed 'Contact for Scientific Queries' as a TRDS field, we have not assumed that this person is the PI, and therefore have separately looked into whether the PI name is compulsory. However, WHO documents [27,29] have contradictory information on this issue. They require that the PI's name, title and email ID be provided, but state that this should be a functional name, not a personal name. It is only if the PI name is compulsory – and preferably recorded in a fixed format [30] – that researchers can quantify the number of unique PIs in a country, ask whether a PI has been taking on too many trials, and so on, and therefore we have assessed this feature.

Second, we looked into the retrospective or prospective registration status of a trial. Prospective registration is crucial to prevent unrecorded 'outcome switching', which creates a bias in the medical evidence base [10]. Nevertheless, it has been argued that (i) it is a duty to trial participants to register each trial, and subsequently publish the results, and (ii) not registering a trial could lead to its loss from the documented universe of trials [31]. As such, retrospective registration is better than non-

registration, and therefore many PR+ permit it. Users may have more confidence in the results of a prospectively than a retrospectively registered trial. Further, flagging retrospective ones may shame the registrants into registering prospectively in future [32]. Accordingly, over half of the PR+ display the registration status of a trial.

Third, it is important to know why a study was terminated [28]. Researchers who have studied the leading causes of trial termination have suggested that the cause should be selected from a fixed set of options [33]. However, only four registries provide this information at all, and only three provide drop-down menus.

Fourth, ISCTR requires that the audit trail of each record should be publicly available, and the Archive module of CTG has been termed a 'powerful tool' [34]. As an example of the use of an audit trail, CONSORT guidelines [35] permit an outcome to be changed mid-trial, provided the reason is specified. As such, we have examined the presence and usefulness of this feature.

Finally, we documented three features that map to other sections of ISCTR. First, the issue of classifying health conditions, which maps to Data Interchange Standards. Comparisons across registries are easier if each one uses a controlled vocabulary, and in particular one that maps to a widely-used metathesaurus [36] as recommended in ISCTR [27]. It is therefore preferable that the health condition be selected from a fixed set of options. However only half the PR+ provide drop-down menus for this field. Second, the security of the website. In the Technical Capacity section, ISCTR requires that each registry have adequate protection against the corruption or loss of data. We have assessed a very basic feature, that is whether the website is secured with an SSL certificate, as is evident when a website URL contains 'https'. Only two-thirds of the PR+ websites do. Third, the issue of documentation. Various documents help users to understand the processes of a registry, or the data it hosts. Although the three documents that we have scored do not strictly map to any section of the ISCTR, they assist users in registering their trial correctly. As such, this feature maps to Quality and Validity.

It is known that users trust public registries more than those created by companies or patient groups [37]. Also, public registries are often the primary sources on which other databases are built [37]. It follows that the information in each one should be comprehensive, high quality and available in a user-friendly fashion. Accordingly, there have been calls for (i) a comparison of such registries, to help develop suitable standards [27], and (ii) ways to improve the accessibility and content of the PR+ [32]. However, several years ago it was shown that there had been non-compliance with the WHO minimal dataset [38], and non-optimal website functionality and user experience [23,24,39]. Since across-the-board improvements have not taken place, this issue needs to be reiterated. However instead of undertaking a purely qualitative assessment, we drew inspiration from other researchers' scorecards. These have either been developed [40–43] or proposed [44,45] to track whether trialists register their studies and report the results accurately, comprehensively and on time. Accordingly, we developed one to assess various features of the PR+.

An ideal registry

We found that the registries show a high degree of variability for a given feature, ranging from a sophisticated version to a routine variant or its complete absence. We have used the best versions of the features analyzed to define an interim ideal registry. In this,

(i) the total number of trials is displayed on the home page;

(ii) a search is possible through (a) a basic search function, (b) each of the TRDS fields, and (c) a few extra fields;

(iii) the data download options include a csv, excel, or tsv format;

(iv) the Brief view is customizable, with 10 or more fields, which can be wrapped;

(v) the Detailed view includes all the TRDS fields;

(vi) there is clarity on whether or not the scientific contact is the PI;

(vii) the PI name is compulsory;

(viii) the reason for the termination of a trial is provided, after being selected from a drop-down menu of possible reasons;

(ix) each trial has an audit trail that enables a comparison of any two versions;

(x) at the very least, the following documents are provided, in English: (a) a definition of each field of the record, (b) a list of FAQs, and (c) one or more user guides;

(xi) the website is secured with an SSL certificate; and

(xii) the health condition category has a drop-down menu to enable trialists to choose a term from a controlled vocabulary, preferably a widely used one.

Although ISCTR recommends several other standards, and it is hoped that all registries will implement all of them in due course, in the interim, all the registries may wish to implement the list above if they have not already done so.

In summary, to the best of our knowledge, this is the first study undertaking a comparative analysis of WHO recognized registries to assess compliance to ISCTR. Our use of a scorecard, based on preset criteria, [illegible]ured an impartial quantification of the quality of the features analyzed. As such, even though our study analyzed a limited set of features, it clearly shows the substantial variation in compliance with the recommended minimal standards. Registries have many users, such as patients and their families, clinicians, researchers, trial sponsors, policy makers and so on. It is the perspective of researchers such as the authors, concerned with the health of the trial ecosystem overall, that has guided the current analysis. Other users may have a different focus, and may wish to alter the assessed fields or the scores. Further, there are other registries, either public or private, and either based on the data in the PR+ or not, which may be interested in the results of this analysis.

This study has a few limitations, as follows: (i) It assesses only some of the many features in each registry. In particular, it does not evaluate any aspect of trial methodology or results, which are crucial sections of such registries. As such, otherwise outstanding registries may have fared less well than expected. (ii) We have not evaluated the completeness of any records or the quality of their data. (iii) Each registry has been evaluated with respect to the

list of fields in a recently registered trial. Earlier records in the same registry may have different content if the required details have changed over time. (iv) We have primarily focused on information that is available in English and may have missed important content in other languages. (v) Although applied systematically, the absolute values of the scores are arbitrary.

Conclusions

Over the years, CTG has received most of the attention of those interested in the accessibility and integrity of the data in public trial registries. As noted above, 41% of the records are held in the other PR+, and a searchlight needs to be turned on them as well. We have identified the best versions of several features that have already been implemented by one or more of these registries, and which serve as pointers on how the others may improve. Running a registry is not merely a bureaucratic task, but is part of a mission to safeguard patients' lives, and the ethics and science of medicine. We hope that our analysis is of some assistance in this. We also believe that our study would be helpful to researchers who may wish to extend this audit and evaluate the completeness of the records or the quality of their data, two other major issues, in all 18 registries.

References

1. Dickersin K, Rennie D. Registering clinical trials. JAMA. 2003 Jul 23;290(4):516-23. doi: 10.1001/jama.290.4.516. PMID: 12876095.

2. Krleza-Jerić K. Clinical trial registration: the differing views of industry, the WHO, and the Ottawa Group. PLoS Med. 2005 Nov;2(11):e378. doi:

10.1371/journal.pmed.0020378. Epub 2005 Oct 18. PMID: 16221000; PMCID: PMC1255765.

3. Lundh A, Krogsbøll LT, Gøtzsche PC. Access to data in industry-sponsored trials. Lancet. 2011 Dec 10;378(9808):1995-1996. doi: 10.1016/S0140-6736(11)61871-0. PMID: 22153200.

4. Lemmens T and Bouchard R, Mandatory Clinical Trial Registration: Rebuilding Public Trust in Medical Research (2007). GLOBAL FORUM UPDATE ON RESEARCH FOR HEALTH, Vol. 4: Equitable Access: Research Challenges for Health in Developing Countries, pp. 40-46, London: Pro-Book Publishing, 2007. Available at SSRN: https://ssrn.com/abstract=1083565.

5. Frost & Sullivan Report. Asia: Preferred Destination for Clinical Trials 2016.

6. Askie LM, Hunter KE, Berber S, Langford A, Tan-Koay AG, Vu T et al. The clinical trials landscape in Australia 2006–2015. Sydney: Australian New Zealand Clinical Trials Registry 2017.

7. Chaturvedi M, Gogtay NJ, Thatte UM. Do clinical trials conducted in India match its healthcare needs? An audit of the Clinical Trials Registry of India. Perspect Clin Res. 2017 Oct-Dec;8(4):172-175. doi: 10.4103/2229-3485.215970. PMID: 29109934; PMCID: PMC5654216.

8. Feizabadi M, Fahimnia F, Jarrahi AM, Naghshineh N, Tofighi S. Iranian clinical trials: An analysis of registered trials in International Clinical Trial Registry Platform (ICTRP). J Evid Based Med. 2017 May;10(2):91-96. doi: 10.1111/jebm.12248. PMID: 28444844.

9. Glasziou P, Altman DG, Bossuyt P, Boutron I, Clarke M, Julious S et al. Reducing waste from incomplete or unusable reports of biomedical research. Lancet. 2014 Jan 18;383(9913):267-76. doi: 10.1016/S0140-6736(13)62228-X. Epub 2014 Jan 8. PMID: 24411647.

10. Goldacre B, Drysdale H, Dale A, Milosevic I, Slade E, Hartley P et al. COMPare: a prospective cohort study correcting and monitoring 58 misreported trials in real time. Trials. 2019 Feb 14;20(1):118. doi: 10.1186/s13063-019-3173-2. PMID: 30760329; PMCID: PMC6375128.

11. Jones CW, Safferman MR, Adams AC, Platts-Mills TF. Discrepancies between ClinicalTrials.gov recruitment status and actual trial status: a cross-sectional analysis. BMJ Open. 2017 Oct 11;7(10):e017719. doi: 10.1136/bmjopen-2017-017719. PMID: 29025842; PMCID: PMC5652524.

12. Nundy S, Gulhati CM. A new colonialism?--Conducting clinical trials in India. N Engl J Med. 2005 Apr 21;352(16):1633-6. doi: 10.1056/NEJMp048361. PMID: 15843665.

13. Potthast R, Vervölgyi V, McGauran N, Kerekes MF, Wieseler B, Kaiser T. Impact of inclusion of industry trial results registries as an information source for systematic reviews. PLoS One. 2014 Apr 17;9(4):e92067. doi: 10.1371/journal.pone.0092067. PMID: 24743113; PMCID: PMC3990559.

14. Scaffidi J, Mol BW, Keelan JA. The pregnant women as a drug orphan: a global survey of registered clinical trials of pharmacological interventions in pregnancy. BJOG. 2017 Jan;124(1):132-140. doi: 10.1111/1471-0528.14151. Epub 2016 Jun 14. PMID: 27297096.

15. Tharyan P, George AT, Kirubakaran R, Barnabas JP. Reporting of methods was better in the Clinical Trials Registry-India than in Indian journal publications. J Clin Epidemiol. 2013 Jan;66(1):10-22. doi: 10.1016/j.jclinepi.2011.11.011. Epub 2012 Mar 27. PMID: 22459428.

16. Institute of Medicine (US) Forum on Drug Discovery, Development, and Translation. Transforming Clinical Research in the United States: Challenges and Opportunities: Workshop Summary. Washington (DC): National Academies Press (US); 2010. doi:10.17226/12900. PMID: 21210556.

17. FDAAA TrialsTracker. EBM DataLab. 2018 [Cited 2019 Nov 14]. Available from: https://fdaaa.trialstracker.net/

18. Turner L. ClinicalTrials.gov, stem cells and 'pay-to-participate' clinical studies. Regen Med. 2017 Sep;12(6):705-719. doi: 10.2217/rme-2017-0015. Epub 2017 Jul 19. PMID: 28721755.

19. Alarcon-Ruiz CA, Roque-Roque JS, Heredia P, Gómez-Briceño AR, Quispe AM. Twenty-two years' experience registering trials in a low-middle income country: The Peruvian Clinical Trial Registry. J Evid Based Med. 2019 Aug;12(3):187-193. doi: 10.1111/jebm.12354. Epub 2019 Jun 18. PMID: 31215157.

20. Chan AW, Song F, Vickers A, Jefferson T, Dickersin K, Gøtzsche PC et al. Increasing value and reducing waste: addressing inaccessible research. Lancet. 2014 Jan 18;383(9913):257-66. doi: 10.1016/S0140-6736(13)62296-5. Epub 2014 Jan 8. PMID: 24411650; PMCID: PMC4533904.

21. Chaturvedi N, Mehrotra B, Kumari S, Gupta S, Subramanya HS, Saberwal G. Some data quality issues at ClinicalTrials.gov. Trials. 2019 Jun 24;20(1):378. doi: 10.1186/s13063-019-3408-2. PMID: 31234923; PMCID: PMC6591874.

22. Fleminger J, Goldacre B. Prevalence of clinical trial status discrepancies: A cross-sectional study of 10,492 trials registered on both ClinicalTrials.gov and the European Union Clinical Trials Register. PLoS One. 2018 Mar 7;13(3):e0193088. doi: 10.1371/journal.pone.0193088. PMID: 29513684; PMCID: PMC5841737.

23. Viergever RF, Ghersi D. The quality of registration of clinical trials. PLoS One. 2011 Feb 24;6(2):e14701. doi: 10.1371/journal.pone.0014701. PMID: 21383991; PMCID: PMC3044717.

24. Viergever RF, Karam G, Reis A, Ghersi D. The quality of registration of clinical trials: still a problem. PLoS One. 2014 Jan 10;9(1):e84727. doi: 10.1371/journal.pone.0084727. PMID: 24427293; PMCID: PMC3888400.

25. Zarin DA, Tse T, Williams RJ, Califf RM, Ide NC. The ClinicalTrials.gov results database--update and key issues. N Engl J Med. 2011 Mar 3;364(9):852-60. doi: 10.1056/NEJMsa1012065. PMID: 21366476; PMCID: PMC3066456.

26. ICTRP Search Portal [Internet]. [cited 2020 Mar 21]. Available from: http://apps.who.int/trialsearch/

27. International Standards for Clinical Trial Registries – Version 3.0. Geneva: World Health Organization; 2018. License: CC BY-NC-SA 3.0 IGO.

28. Committee on Clinical Trial Registries; Board on Health Sciences Policy; Institute of Medicine. Developing a National Registry of Pharmacologic and Biologic Clinical Trials: Workshop Report. Washington (DC): National Academies Press (US); 2006. doi:10.17226/11561. PMID: 26740992.

29. WHO | WHO Data Set [Internet]. [cited 2019 Nov 14]. Available from: https://www.who.int/ictrp/network/trds/en/

30. Pillamarapu M, Mohan A, Saberwal G. An analysis of deficiencies in the data of interventional drug trials registered with Clinical Trials Registry - India. Trials. 2019 Aug 28;20(1):535. doi: 10.1186/s13063-019-3592-0. PMID: 31455366; PMCID: PMC6712861

31. Harriman SL, Patel J. When are clinical trials registered? An analysis of prospective versus retrospective registration. Trials. 2016 Apr 15;17:187. doi: 10.1186/s13063-016-1310-8. PMID: 27079379; PMCID: PMC4832501.

32. WHO ICTRP Registry Network Meeting Summary Report [Internet]. Geneva: World Health Organization; 2015. Available from: https://www.who.int/ictrp/news/ICTRP_registry_network_meeting_report_2015_web.pdf?ua=1

33. Williams RJ, Tse T, DiPiazza K, Zarin DA. Terminated Trials in the ClinicalTrials.gov Results Database: Evaluation of Availability of Primary Outcome Data and Reasons for Termination. PLoS One. 2015 May 26;10(5):e0127242. doi: 10.1371/journal.pone.0127242. PMID: 26011295; PMCID: PMC4444136

34. Huić M, Marušić M, Marušić A. Completeness and changes in registered data and reporting bias of randomized controlled trials in ICMJE journals after trial registration policy. PLoS One. 2011;6(9):e25258. doi: 10.1371/journal.pone.0025258. Epub 2011 Sep 21. PMID: 21957485; PMCID: PMC3177887.

35. Moher D, Hopewell S, Schulz KF, Montori V, Gøtzsche PC, Devereaux PJ, Elbourne D, Egger M, Altman DG. CONSORT 2010 explanation and elaboration: updated guidelines for reporting parallel group randomised trials. Version 2. BMJ. 2010 Mar 23;340:c869. doi: 10.1136/bmj.c869. PMID: 20332511; PMCID: PMC2844943.

36. Metathesaurus [Internet]. [cited 2020 Mar 22]. Available from: https://www.nlm.nih.gov/research/umls/knowledge_sources/metathesaurus/index.html

37. The-Clinical-Trial-Registry-of-the-Future-Proposal.pdf [Internet]. [cited 2020 Mar 22]. Available from: http://www.transceleratebiopharmainc.com/wp-content/uploads/2017/11/The-Clinical-Trial-Registry-of-the-Future-Proposal.pdf

38. Moja LP, Moschetti I, Nurbhai M, Compagnoni A, Liberati A, Grimshaw JM, Chan AW, Dickersin K, Krleza-Jeric K, Moher D, Sim I, Volmink J. Compliance of clinical trial registries with the World Health Organization minimum data set: a survey. Trials. 2009 Jul 22;10:56. doi: 10.1186/1745-6215-10-56. PMID: 19624821; PMCID: PMC2734552.

39. Ogino D, Takahashi K, Sato H. Characteristics of clinical trial websites: information distribution between ClinicalTrials.gov and 13 primary registries in the WHO registry network. Trials. 2014 Nov 5;15:428. doi: 10.1186/1745-6215-15-428. PMID: 25373358; PMCID: PMC4234832.

40. Miller JE, Wilenzick M, Ritcey N, Ross JS, Mello MM. Measuring clinical trial transparency: an empirical analysis of newly approved drugs and large pharmaceutical companies. BMJ Open. 2017 Dec 5;7(12):e017917. doi: 10.1136/bmjopen-2017-017917. PMID: 29208616; PMCID: PMC5728266.

41. Goldacre B. How to get all trials reported: audit, better data, and individual accountability. PLoS Med. 2015 Apr 14;12(4):e1001821. doi: 10.1371/journal.pmed.1001821. PMID: 25874719; PMCID: PMC4396123.

42. Our scoring method [Internet]. [cited 2020 Mar 21]. Available from: http://policyaudit.alltrials.net/our-scoring-method/

43. Goldacre B, Gray J. OpenTrials: towards a collaborative open database of all available information on all clinical trials. Trials. 2016 Apr 8;17:164. doi: 10.1186/s13063-016-1290-8. PMID: 27056367; PMCID: PMC4825083.

44. Azar M, Riehm KE, Saadat N, Sanchez T, Chiovitti M, Qi L et al. Evaluation of Journal Registration Policies and Prospective Registration of Randomized Clinical Trials of Nonregulated Health Care Interventions. JAMA Intern Med. 2019 May 1;179(5):624-632. doi: 10.1001/jamainternmed.2018.8009. PMID: 30855655; PMCID: PMC6503638.

45. Miller J, Ross JS, Wilenzick M, Mello MM. Sharing of clinical trial data and results reporting practices among large pharmaceutical companies: cross sectional descriptive study and pilot of a tool to improve company practices. BMJ. 2019 Jul 10;366:l4217. doi: 10.1136/bmj.l4217. PMID: 31292127; PMCID: PMC6614834.

Supporting information

S1 Table. Mapping to ISCTR. The 17 features analyzed in this study map to the following nine sections of ISCTR: (i) Content, (ii) Quality and Validity, (iii) Accessibility, (iv) Unambiguous Identification, (v) Technical Capacity, (vi) Administration and Governance, (vii) The 24-field TRDS, (viii) Partner Registries, and (ix) Data Interchange Standards.

S2 Table. Data on six aspects of each registry. (a) Time taken to obtain the results of a search, (b) Total number of trials in the registry, (c) Existence of a basic search function, (d and e) Advanced search function – TRDS fields and Extra fields, and (f) Data download options.

S3 Table. The list of the TRDS fields that are present in the Brief view and the Detailed view. The presence or absence of the field is indicated by a 1 or 0, respectively. The number of the sample trial used for each registry is also provided.

S4 Table. For each registry, a listing of the Extra fields in the Brief and Detailed views.

S5 Table. Data on multiple aspects of each registry. (a) whether the PI name is compulsory; (b) reason for the termination of a trial, and whether there is a drop-down menu of reasons; (c) audit trail; (d) health condition (e) SSL certificate, and (f) documentation.

Click here to access/download
Supporting Information
S1 Table.xlsx

Click here to access/download
Supporting Information
S2 Table.xlsx

Click here to access/download
Supporting Information
S3 Table.xls

Click here to access/download
Supporting Information
S4 Table.xlsx

Click here to access/download

Supporting Information

S5 Table.xlsx
