# Supplementary material for: A comparative analysis of important public clinical trial registries, and a proposal for an interim ideal one
Source: PLoS One. 2021 May 11;16(5):e0251191. doi: 10.1371/journal.pone.0251191 (PMC8112656; doi:10.1371/journal.pone.0251191)
Supplement: Attachment — Submitted filename: PONE-D-20-16268_R1_Feedback.pdf [file pone.0251191.s008.pdf]

PLOS ONE

A comparative analysis of important public clinical trial registries, and a proposal for an interim ideal one

--Manuscript Draft--

**Manuscript Number:**	PONE-D-20-16268R1
**Article Type:**	Research Article
**Full Title:**	A comparative analysis of important public clinical trial registries, and a proposal for an interim ideal one
**Short Title:**	Important public clinical trial registries
**Corresponding Author:**	Gayatri Saberwal, Ph.D.<br>Institute of Bioinformatics and Applied Biotechnology<br>Bangalore, Karnataka INDIA
**Keywords:**	Clinical trial registry; Primary registry; ICTRP; ClinicalTrials.gov; ethics; Regulatory issues
**Abstract:**	Background<br>It is an ethical and scientific obligation to register each clinical trial, and report its results, accurately, comprehensively and on time. The WHO recognizes 17 public registries as Primary Registries, and has also introduced a set of minimal standards in the International Standards for Clinical Trial Registries (ISCTR) that primary registries need to implement. These standards are categorized into nine sections — Content, Quality and Validity, Accessibility, Unambiguous Identification, Technical Capacity, Administration and Governance, the Trial Registration Data Set (TRDS), Partner registries and Data Interchange Standards. This study compared the WHO's primary registries, and the US's ClinicalTrials.gov, to examine the implementation of ISCTR, with the aim of defining features of an interim ideal registry.<br><br>Methods and Findings<br>The websites of the 18 registries were evaluated for 14 features that map to one or more of the nine sections of ISCTR, and assigned scores for their variations of these features. The assessed features include the nature of the content; the number and nature of fields to conduct a search; data download formats; the nature of the audit trail; the health condition category; the documentation available on a registry website; and so on. Overall, the registries received between 27% and 80% of the maximum score of 94. The results from our analysis were used to define a set of features of an interim ideal registry.<br><br>Conclusions<br>To the best of our knowledge, this is the first study to quantify the widely divergent quality of the primary registries' compliance with the ISCTR. Even with this limited assessment, it is clear that some of the registries have much work to do, although a mere dozen improvements would significantly improve them.
**Order of Authors:**	Nisha Venugopal
	Gayatri Saberwal, Ph.D.
**Response to Reviewers:**	Response to Reviewers<br><br>REVIEWER #1: Overall this is a useful and very timely piece of work which, as the authors say, could trigger further work on registry assessment and a wider debate on how trial registries can both improve the features they offer and become more consistent. In general it is well written and well referenced, and is supported by a comprehensive set of detailed data as supplementary files. The authors acknowledge the limitations of their study and include a useful set of suggestions for an 'ideal registry' as an aspiration to work towards.<br><br>I have some reservations about some aspects of the paper, however, which I think

detract from its overall quality – but which I hope can be easily rectified:
Authors: We thank the reviewer for the appreciative comments.

- - - - - - - - - - - - - - - - - -
1) I found the organisation of some of the material confused. In particular the very short methods section provides little detail about the 17 features selected as the basis of assessment, how and why they were selected, and by whom, and how decisions on weighting were made, and why 3 were not assessed. Later on, in table 2b and as a large part of the 'Discussion of specific features…' in Box 1, much of this material is covered, but I think it would have been simpler and more logical to bring these explanations together as part of an expanded methods section. Box 1 is embedded in the discussion but its content seems largely a justification of the scorecard's construction. The result is that the reader has to work harder than they should to understand how and why the scoring system was constructed.
Authors: We have reorganized the Methods section, and added further details. This includes portions from Box 1. We have also moved Table 2b to the methods section (where it is now Table 1) to make the rationale of the scorecard available upfront. Additionally, we wish to highlight that lines 128–130 describe how the authors selected the criteria for the scorecard, based on a review of the literature, but mainly focussing on the ISCTR guidelines.

- - - - - - - - - - - - - - - - - -
2) Similarly I think the results section could be better organised. Why not simply go through the results for each of the 14 areas assessed, noting at that point the median and the range of scores, techniques and difficulties in assessment, and possible caveats around the scores obtained? The current section provides useful tables and a brief summary, but much of the text is simply restating what was accessed. Would a simple pie chart be a useful way of summarising the total numbers data in table 1, to show the proportion of total registry entries included in each?
Authors: We have rewritten and reorganized the Results and Discussion. We have created the suggested pie chart, and have also presented other data from the erstwhile Table 1 as a figure.

- - - - - - - - - - - - - - - - - -
3) A minor point, but there 10 superscript references in Table 1, presumably to some explanatory notes about the data point presented, but I could not find any explanation for them, either in the main text or the supplementary material. They should either be removed or (better) the explanatory notes should be provided.
Authors: These notes were inadvertently left out due to the complications of submitting large tables in a particular format. They are visible in the revised Table.

- - - - - - - - - - - - - - - - - -
4) I thought the discussion was a little timid. The work was done in early 2020, in the context of a pandemic that has dramatically underscored the need for good quality, consistent and easily available information from trial registries, partly to be able to track the numbers, types and results of trials relating to COVID-19, partly because public health decisions require a network of data sources at a global level and registries should be a key part of this. That point might have been worth including – improving trial registry systems has become more urgent!
Authors: We have rewritten the discussion, which include the following lines.
“The ongoing Covid-19 pandemic has forcefully brought home the need for high quality trial registries with information that is consistent, comprehensive and available in a user-friendly fashion. Billions of people need to be immediately protected from the virus, and large numbers of drugs and vaccines are in trials. There is world-wide interest in these trials, and information that is being tracked includes what is being trialled; where are these trials taking place; and what are the results of these trials? Each country needs to take public health decisions, which will evolve as trials running in different parts of the world yield results. Public trial registries are one of the fastest ways of communicating these results.

Further, the publicly available, freely accessible information in trial registries helps to build trust with the public [26,44]. Covid-19 trials have been among the fastest recruiting trials in history [50,51], and it is possible that the publicly available information in trial registries has helped many of the potential trial participants decide to

enrol.

It is not just that everyone is interested in the positive outcomes of trials. For example, an inspection of the CTRI records of hundreds of covid-19 trials being run in India has thrown up quality issues in almost all of them. Based on negative publicity, the government has taken action in some cases [52].”

- - - - - - - - - - - - - - - - - -

5) Similarly, although there is a general sentiment expressed that registries should improve, there were no concrete suggestions as to how this might be achieved or who needs to be involved, e.g. by greater collaboration between registries, perhaps orchestrated by the WHO, or by using the influence of funders and publishers to re-iterate the need for greater consistency. Are some of the aspects that were assessed easier to improve than others? If so how could they be progressed? Should there be a web page with a regularly updated 'score card' for the trial registries? I appreciate this was an initial survey but I think it might have been useful to venture, if only briefly, into this area in the discussion.

Authors: We have added the following lines to the Results and Discussion:

There is a long history of various stakeholders arguing for the need to improve registries and the quality of trial registration. Examples include academics and health activists [53–55], journals (ICMJE) [56], WHO [41], registry managers [57], funders [58,59] and governments [60]. Each of these efforts has led to some improvements in the number and quality of trial records hosted by registries. However none of them has led to a perfect set of records. It is likely that the only way this will be achieved is if all stakeholders continue to apply pressure on the registries. Studies such as this one help to highlight deficiencies, which adds to the other efforts aimed at improving registries. Further, the authors would welcome other researchers’ efforts to create and update a website that lists the scorecard, with periodic updates. Should such a website not be created by any other group, the authors intend to re-evaluate the registries’ performance on the scorecard every few years.

- - - - - - - - - - - - - - - - - -

6) Another issue largely missing from the discussion: the authors mention that registries have many different types of users – researchers, clinicians, members of the public, data scientists, etc. I wonder if this should therefore lead to different scoring systems – perhaps with different weightings and / or items – for each of those major user groups. Those could provide additional insight into the strengths and weakness of different repositories, and thus more clearly identify areas of improvement, but could also be consolidated into an overall score if desired. For example, although the authors state their assumption is that most users would not have the technical expertise to use APIs, and / or scraping and crawling systems to retrieve data, the integration of trial registries with other data systems, and thus the ability to support bulk download by machines, is becoming increasingly important. I would have liked to have seen this aspect more explicitly included in any 'to do list' of possible future assessments, along with considerations of data quality, completeness, and the support for reporting results

Authors: In the Discussion, while enumerating the various kinds of users of registry data, we have stated that “Other categories of users, such as medical professionals, patients, trial sponsors, policy makers, data scientists and so on, may wish to alter the assessed features, or the scoring, in order to rank the registries according to their priorities. For instance, a data scientist would be very appreciative of ANZCTR, which specifically enables web crawling of its records [49]. Furthermore, the managers of other registries, either public or private, and either based on the data in the PR+ or not, may be interested in the results of this study.”. We do not feel confident of creating different scoring systems. Ideally, this should be done by polling at least a few individuals in each category of users, and we would find it extremely challenging to do this in India. As such, any additional scoring system that we developed would be based on unvalidated assumptions, and would be unconvincing, even to us.

- - - - - - - - - - - - - - - - - -

7) There is a minor but distracting typo in the first paragraph of the Results section (5,72,901)

Authors: We had used the Indian system. We have corrected this to 572,901.

- - - - - - - - - - - - - - - - - -

Powered by Editorial Manager® and ProduXion Manager® from Aries Systems Corporation

Having listed all of the points above I would re-iterate that overall I think the paper is useful and should be published. The points are offered as suggestions for possible improvement.

REVIEWER #2:
The concept is interesting but needs to be re-written.

1. The paper should first start with a good explanation of the origins of the ISCTR. For example, "following the Ministerial Summit on Health Research that took place in Mexico City, Mexico, in November 2004, participants called for the WHO to facilitate the establishment of: "a network of international clinical trials registers to ensure a single point of access and the unambiguous identification of trials".
Authors: We have rewritten the Introduction to include these events and further details of the ICTRP.

- - - - - - - - - - - - - - - - - -
2. https://www.who.int/ictrp/about/en/ The authors need to be more complete in explaining the WHO registry network including primary vs partner registries as well as data providers; the differences of each. Then as it relates to registries what kind of papers have been published; findings; some of this is introduced at a high level in the discussion section which belongs in the introduction.
Authors: We have include these points in the Introduction.

- - - - - - - - - - - - - - - - - -
3. The authors are not clear in their terminology (for example, versions vs features). There is reference to WHO's 24-field Trial Registration Data Set vs 17 features vs 14 features selected by the authors; there is reference to the 9 standards; hence it is not clear how these "concepts" inter-relate (24 vs 17 vs 9; data set vs standards) and why the authors selected 14 features (which is perhaps more attributes than features).
Authors: We have now ensured the following:
(a) the word ‘section’ only refers to one or more of the nine sections of the ISCTR;
(b) the word ‘feature’ only refers to one or more of the 14 features of each registry that are the focus of this study;
(c) since the different registries may have different variants of each ‘feature’, we use the word ‘variation’ in this context.
We have also included this list as a Box within Methods so that readers have no confusion regarding the terminology used.

- - - - - - - - - - - - - - - - - -
4. How does WHO refer to author defined "features".
Authors: As described in Methods, the ‘features’ defined in this study have been compiled by the authors from different sources, including ISCTR. Therefore ISCTR discusses some, but not all, of these features directly. However, each feature maps to one or more standards set forth in ISCTR.

- - - - - - - - - - - - - - - - - -
5. The score card is hard to follow; the scoring is not understandable (for example, "for features with multiple variants, the score ranges from 1 to 5" is not clear and then how scores were calculated: as in the case of chCTR for advanced search fields TRDS a score of 17 was assigned).
Authors: We have reorganized the Methods section, and added further details. This includes portions from Box 1. We have also moved Table 2b to the methods section (where it is now Table 1) to make the rationale of the scorecard available upfront. Further, we have rewritten the Results and Discussion to include a more detailed analysis of the findings. We hope this alleviates the confusion around the scorecard.

- - - - - - - - - - - - - - - - - -
6. The meaning of the scorecard is not clear; the interpretation of the findings are inconsistent and leaves the reader bewildered.
Authors: We regret that the first version of the manuscript was so confusing. Please refer to our response to the comment before this (Comment 5).

- - - - - - - - - - - - - - - - - -

7. Please see specific comments in the attached word document.
Authors: Please find below a response to each of the comments in the manuscript file, which we have numbered from 7.1 to 7.44). In each case we have referenced the line in the original pdf, where the reviewer's comment has been taken from.

- - - - - - - - - - - - - - - - - -
7.1 Perhaps to elaborate on the 14 features briefly: what do they cover off on. (Line 31)
Authors: This has been done. [Lines 32–35, and 41–44 of the revised manuscript]

- - - - - - - - - - - - - - - - - -
7.2 Would not include limitations here. Only in the body of the paper under the proper section. (Line 42)
Authors: This has been done.

- - - - - - - - - - - - - - - - - -
7.3 New information should not be introduced in the conclusion; rather include in the results section. (Line 51)
Authors: This has been done. [Lines 587–589 of the revised manuscript]

- - - - - - - - - - - - - - - - - -
7.4 such as? Elaborate (Line 53)
Authors: This has been done. [Lines 63–68 of the revised manuscript]

- - - - - - - - - - - - - - - - - -
7.5 elaborate on types of comments and analyses. (Line 67)
Authors: This has been done. [Lines 72–76 of the revised manuscript]

- - - - - - - - - - - - - - - - - -
7.6 awkwardly written. "Set up to facilitate"? The ICTRP was designed to help facilitate. (Line 68)
Authors: This has been reworded. [Lines 81–85 of the revised manuscript]

- - - - - - - - - - - - - - - - - -
7.7 replace wording with "not an ICTRP recognized registry"... (Line 71)
Authors: This has been reworded. [Lines 85–88 of the revised manuscript]

- - - - - - - - - - - - - - - - - -
7.8 why were the other partner registries not included? or at least some of the other partner registries? (Line 74)
Authors: We have not included *any* partner registry in our analysis. This issue has been covered in more detailed now. [Lines 88–93 of the revised manuscript]

- - - - - - - - - - - - - - - - - -
7.9 Write as Primary Registries Plus (PR+) (Line 74)
Authors: This has been done. [Lines 92–93 of the revised manuscript]

- - - - - - - - - - - - - - - - - -
7.10 Define the nine sections... (Line 77)
Authors: This has been done. [Lines 97–100 of the revised manuscript]

- - - - - - - - - - - - - - - - - -
7.11 Recommend using 3rd person objective. Not "we" (Line 77)
Authors: It would be extremely challenging to rewrite the Methodology in 3rd person. However, we have ascertained that 'we' is used in the Methods' section, in articles that have appeared in well-known journals including PLOS ONE such as:
https://www.bmj.com/content/362/bmj.k3218
https://trialsjournal.biomedcentral.com/articles/10.1186/1745-6215-15-428
https://journals.plos.org/plosone/article?id=10.1371/journal.pone.0193088#sec006
We hope that it is alright if we leave the construction as it is.

- - - - - - - - - - - - - - - - - -
7.12 not clear: using terms like versions, features, sections - difficult for the reader to follow (Line 83)
Authors: We have addressed this in our response to this Reviewer's point 3.

- - - - - - - - - - - - - - - - - -
7.13 important to note that not all fields in clinicaltrial.gov are mandatory. is this the case in other registries as well? (Line 96)
Authors: Yes, it is true that all fields are not mandatory in any registry. However we are only examining the *presence* of certain fields, not whether trialists have filled each of them.

- - - - - - - - - - - - - - - - - -
7.14 Authors? (Line 98)
Authors: We have rewritten this sentence and it now reads, ‘All analyses were performed by one author (NV) and verified by the other (GS).”

- - - - - - - - - - - - - - - - - -
7.15 Is this part of Methods (Line 102)
Authors: We have formatted the manuscript to more clearly demarcate the different levels of headings.

- - - - - - - - - - - - - - - - -
7.16 needs to be defined "multiple variants, score ranges" (Line 106)
Authors: We have defined the variants and the score ranges in Table 1.

- - - - - - - - - - - - - - - - - -
7.17 difficult to follow; confusing (Line 107)
Authors: We have moved Table 2b to the methods section (where it is now Table 1) to make the rationale of the scorecard available upfront. We have also illustrated our scoring system with examples early in the Methods section. Additionally, we wish to highlight that lines 128–130 describe how the authors selected the criteria for the scorecard, based on literature review but mainly focussing on the ISCTR guidelines.

- - - - - - - - - - - - - - - - - -
7.18 perhaps to refer to as "attributes"? (Line 117)
Authors: We have determined that ‘feature’ and ‘attribute’ are synonyms. Since we have used ‘features’ throughout the manuscript, we preferred to stick to it. Also, as detailed in our response to the comment 7.12, above, we have rationalized our use of the word ‘features’ so that there is no confusion over its usage.

- - - - - - - - - - - - - - - - - -
7.19 why 14 of the 17? (Line 118)
Authors: While revising the manuscript, we have removed the three features that were not assessed quantitatively. This leaves 14 features, all of which are in the scorecard. We believe that these changes have removed room for confusion on this point, and improved the readability of the paper.

- - - - - - - - - - - - - - - - - -
7.20 we went from 14 of 17 to 24. Not sure how this ties back to the 9 standards introduced under Methods (Line 120)
Authors: As mentioned in point 7.19, above, we have removed the three features that were assessed quantitatively, and the final number is 14.
Regarding the number 24: As described in the manuscript, one of the 14 features is the WHO’s Trial Registration Data Set, or TRDS, which in turn is composed of 24 distinct fields (as defined by ISCTR).

- - - - - - - - - - - - - - - - - -
7.21 weight not weightage (Line 123)
Authors: This has been rewritten. [Lines 229–233 of the revised manuscript]

- - - - - - - - - - - - - - - - - -
7.22 why is this a feature of the "registry" vs what is required by government in the local country. (Line 133)
Authors: In order to avoid possible confusion, we have rephrased the sentence to: “As a first step, it is important to know how many records the database holds. This number should be readily available, and we have therefore analysed the ease of accessing it.”

- - - - - - - - - - - - - - - - - -
7.23 why were these two subjects highlighted especially as they were not noteworthy or substantive. (Line 136)
Authors: We have deleted these two features now.

- - - - - - - - - - - - - - - - - -
7.24 is this a server issue; connectivity; (Line 140)
Authors: We do not know the reason for the lag in loading RPCEC results. It seems to be at the RPCEC end, since other registries gave us no problem. In any case, we have deleted the entire feature now.

- - - - - - - - - - - - - - - - - -
7.25 would think this is an important aspect to score; the logic to not score this as half of the registries did not contain is a shortcoming of the analysis as registries that do not have this attribute should receive a lower rating. Completion of variables is less about the registry vs the owner of the "data" (sponsor). (Line 140)
Authors: We have now included in the Results and Discussion our analysis of whether the registry provides the reason for trial termination. However, the information pertaining to this feature is already present in the Extra fields. Hence we have not scored this features separately, since that would result in it being double counted.

- - - - - - - - - - - - - - - - - -
7.26 why these attributes? Why is SSL important? (Line 154)
Authors: We have now included a more detailed description of why SSL is important and why we have included this feature. [Lines 471–481 of the revised manuscript]

- - - - - - - - - - - - - - - - - -
7.27 this is the sponsor's responsibility: QC (Line 166)
Authors: In the Results and Discussion section we have now described why even though QC is the sponsor's responsibility it does not always do this, and how it is the duty of the registry to facilitate higher quality registrations. [Lines 496–501 of the revised manuscript]

- - - - - - - - - - - - - - - - - -
7.28 these points should have been provided in the introduction. (Line 181)
Authors: We have now included these points in the Introduction.

- - - - - - - - - - - - - - - - - -
7.29 there is no enough discussion on what has been done, why there is a gap and how this fills the gap in a meaningful way. (Line 189)
Authors: We have rewritten the Introduction and have discussed the work done so far, and how our study fills a lacuna in the analysis and reporting of registries' performance.

- - - - - - - - - - - - - - - - - -
7.30 why is this ideal? (Line 195)
Authors: As described in the manuscript, we propose an 'interim ideal' registry based on the features that we have assessed. That it is a limited goal on the way to achieving everything that ISCTR requires. And it is the 'ideal' from amongst the various options that one or more of the registries are already using.

- - - - - - - - - - - - - - - - - -
7.31 seems per line 180 to 189 there have been other studies. not clear. (Line 215)
Authors: We believe that the revised manuscript addresses this concern.

- - - - - - - - - - - - - - - - - -
7.32 how was this impartial? (Line 216)
Authors: Primarily based on the recommendations of ISCTR, we determined which features of the registries to assess. All scoring rationales were also based on the minimum standards outlined in the ISCTR, and recommendations from earlier studies in our literature survey. We believe that our scoring is impartial since this protocol rules out scoring that may be biased in favour of, or against, any particular registry.

- - - - - - - - - - - - - - - - - -

Powered by Editorial Manager® and ProduXion Manager® from Aries Systems Corporation

	7.33 over reach as this is a subjective statement; only state represents the authors' perspective not that of others. (Line 220)<br>Authors: We have changed this sentence, which now reads “The scorecard above analyses features that are of interest to the authors and, by extension, possibly to other researchers concerned with the health of the trial ecosystem overall.”<br><br>- - - - - - - - - - - - - - - - - -<br>7.34 not clear (Line 222)<br>Authors: We have rephrased this to the following: “Other categories of users, such as medical professionals, patients, trial sponsors, policy makers, data scientists and so on, may wish to alter the assessed features, or the scoring, in order to rank the registries according to their priorities.”<br><br>- - - - - - - - - - - - - - - - - -<br>7.35 registries cannot be interested in something; only those who work on registries (Line 224)<br>Authors: We have rephrased the sentence to the following: “Further, the managers of other registries, either public or private, and either based on the data in the PR+ or not, may be interested in the results of this analysis.”<br><br>- - - - - - - - - - - - - - - - - -<br>7.36 less about the registry and more about how users utilize unless all fields are required by the registry. (Line 230)<br>Authors: We are not sure that we have understood this question. It is true that in no registry are all fields mandatory. Thus it would not be advisable to evaluate the quality of registration, and of information in a particular field, by comparisons across registries. However, as we have described in our response to Reviewer comment 7.13, we are only examining the presence of certain fields, not whether trialists have filled each of them.<br><br>- - - - - - - - - - - - - - - - - -<br>7.37 need to write using English proper language fit for scientific publication (Line 238)<br>Authors: We have rephrased the sentence to the following: “As noted above, 41% of the records are held in the other PR+, and they need to be examined as well.”<br><br>- - - - - - - - - - - - - - - - - -<br>7.38 Define (Page 19, Box 1)<br>Authors: We have described our assessment of this feature in greater detail in the revised manuscript.<br><br>- - - - - - - - - - - - - - - - - -<br>7.39 more importantly why do registries include something not recommended by ISCTR? (Page 20, Box 1)<br>Authors: We have added the following lines to the manuscript: “There may be a range of reasons for including these Extra fields. For instance, India had been criticized for the lack of appropriate oversight to ensure the ethical conduct of trials, and therefor CTRI asked trialists for details of the ethics committee even before ISCTR required this information [38,39]. Also, there have been demands from the Cochrane collaboration, and many other individuals and groups, to include several additional items in the ISCTR list, which WHO has not agreed to. It is alleged that the recommended list is closer to what industry demanded [40]. As such, although ISCTR may not list every field that many people believed to be essential, managers of particular registries may have chosen to list some of them.”<br><br>- - - - - - - - - - - - - - - - - -<br>7.40 Restructure: what does WHO require; what do registries do; where is the difference and impact on value of registries. (Page 20, Box1)<br>Authors: In each section of the Results and Discussion, we provide an introductory line, then the results, then the discussion. We have done this, incorporating the Reviewer’s points in this section.<br><br>- - - - - - - - - - - - - - - - - -<br>7.41 is this an author term? It is a prospective trial even if data is only entered after the study starts. (Page 21, Box1)

	Authors: 'Retrospective trials' is used by other researchers – it is not our term. Nevertheless, earlier in the manuscript, we have added an explanatory line "Trials may be registered either prospectively or retrospectively, that is before the enrolment of the first participant or after." Also, in order to avoid ambiguity, we have rephrased this sentence as follows: "Further, flagging retrospectively registered ones may shame the registrants into registering prospectively in future".<br><br>- - - - - - - - - - - - - - - - - -<br>7.42 what were the findings? (Page 21, Box1)<br>Authors: We have revised the text to include the findings of the analysis.<br><br>- - - - - - - - - - - - - - - - - -<br>7.43 how was 3 assigned out of 5: what does a value of 1 or 2 or 3 or 4 or 5 represent? (Page 8, Table 2a)<br>Authors: As explained in the response to the Reviewer's comment 5, we have moved Table 2b to the methods section (where it is now Table 1) to make the rationale of the scorecard available upfront. We hope this will alleviate the confusion regarding scoring.<br><br>- - - - - - - - - - - - - - - - - -<br>7.44 11? 17? thought scores were 1 to 5. (Page 8, Table 2a)<br>Authors: Please refer our response to the previous point (Reviewer's comment 7.43)<br><br>- - - - - - - - - - - - - - - - - -<br>8. The topic is interesting but the paper needs to be rewritten and the concept of a scorecard has to be rethought to ensure it has logical relevance to the reader, that the scoring is understandable and can be interpreted thereby leading to actionable insights.<br>Authors: We have rewritten and reorganized the manuscript, and we hope that these changes address the reviewer's concerns.<br><br>- - - - - - - - - - - - - - - - - -<br>9. The paper has to be placed in context of other relevant studies completed to date.<br>Authors: We have rewritten the Introduction to address this concern.
**Additional Information:**	
**Question**	**Response**
**Financial Disclosure**<br><br>Enter a financial disclosure statement that describes the sources of funding for the work included in this submission. Review the submission guidelines for detailed requirements. View published research articles from *PLOS ONE* for specific examples.<br><br>This statement is required for submission and **will appear in the published article** if the submission is accepted. Please make sure it is accurate.	GS received internal institutional funds. These were partially from the Government of Karnataka's Department of Information Technology, Biotechnology and Science & Technology (https://itbtst.karnataka.gov.in/english). There was no grant number.<br><br>The funders had no role in study design, data collection and analysis, decision to publish, or preparation of the manuscript.

**Unfunded studies**<br>Enter: *The author(s) received no specific funding for this work.*<br><br>**Funded studies**<br>Enter a statement with the following details:<br>• Initials of the authors who received each award<br>• Grant numbers awarded to each author<br>• The full name of each funder<br>• URL of each funder website<br>• Did the sponsors or funders play any role in the study design, data collection and analysis, decision to publish, or preparation of the manuscript?<br>• **NO** - Include this sentence at the end of your statement: *The funders had no role in study design, data collection and analysis, decision to publish, or preparation of the manuscript.*<br>• **YES** - Specify the role(s) played.<br><br>* typeset	
**Competing Interests**<br><br>Use the instructions below to enter a competing interest statement for this submission. On behalf of all authors, disclose any competing interests that could be perceived to bias this work—acknowledging all financial support and any other relevant financial or non-financial competing interests.<br><br>This statement **will appear in the published article** if the submission is accepted. Please make sure it is accurate. View published research articles from *PLOS ONE* for specific examples.	The authors have declared that no competing interests exist.

Powered by Editorial Manager® and ProduXion Manager® from Aries Systems Corporation

**NO authors have competing interests**<br><br>Enter: *The authors have declared that no competing interests exist.*<br><br>**Authors with competing interests**<br><br>Enter competing interest details beginning with this statement:<br><br>*I have read the journal's policy and the authors of this manuscript have the following competing interests: [insert competing interests here]*<br><br>* typeset	
**Ethics Statement**<br><br>Enter an ethics statement for this submission. This statement is required if the study involved:<br><br>• Human participants<br>• Human specimens or tissue<br>• Vertebrate animals or cephalopods<br>• Vertebrate embryos or tissues<br>• Field research<br><br>Write "N/A" if the submission does not require an ethics statement.<br><br>General guidance is provided below. Consult the submission guidelines for detailed instructions. **Make sure that all information entered here is included in the Methods section of the manuscript.**	Not applicable

**Format for specific study types**<br><br>**Human Subject Research (involving human participants and/or tissue)**<br>• Give the name of the institutional review board or ethics committee that approved the study<br>• Include the approval number and/or a statement indicating approval of this research<br>• Indicate the form of consent obtained (written/oral) or the reason that consent was not obtained (e.g. the data were analyzed anonymously)<br><br>**Animal Research (involving vertebrate animals, embryos or tissues)**<br>• Provide the name of the Institutional Animal Care and Use Committee (IACUC) or other relevant ethics board that reviewed the study protocol, and indicate whether they approved this research or granted a formal waiver of ethical approval<br>• Include an approval number if one was obtained<br>• If the study involved *non-human primates*, add *additional details* about animal welfare and steps taken to ameliorate suffering<br>• If anesthesia, euthanasia, or any kind of animal sacrifice is part of the study, include briefly which substances and/or methods were applied<br><br>**Field Research**<br><br>Include the following details if this study involves the collection of plant, animal, or other materials from a natural setting:<br>• Field permit number<br>• Name of the institution or relevant body that granted permission	
**Data Availability**<br><br>Authors are required to make all data underlying the findings described fully available, without restriction, and from the time of publication. PLOS allows rare exceptions to address legal and ethical concerns. See the PLOS Data Policy and FAQ for detailed information.	Yes - all data are fully available without restriction

A Data Availability Statement describing where the data can be found is required at submission. Your answers to this question constitute the Data Availability Statement and **will be published in the article**, if accepted.<br><br>**Important:** Stating 'data available on request from the author' is not sufficient. If your data are only available upon request, select 'No' for the first question and explain your exceptional situation in the text box.<br><br>Do the authors confirm that all data underlying the findings described in their manuscript are fully available without restriction?	
**Describe where the data may be found in full sentences. If you are copying our sample text, replace any instances of XXX with the appropriate details.**<br><br>• If the data are **held or will be held in a public repository**, include URLs, accession numbers or DOIs. If this information will only be available after acceptance, indicate this by ticking the box below. For example: *All XXX files are available from the XXX database (accession number(s) XXX, XXX.*).<br>• If the data are all contained **within the manuscript and/or Supporting Information files**, enter the following: *All relevant data are within the manuscript and its Supporting Information files.*<br>• If neither of these applies but you are able to provide **details of access elsewhere**, with or without limitations, please do so. For example:<br><br>*Data cannot be shared publicly because of [XXX]. Data are available from the XXX Institutional Data Access / Ethics Committee (contact via XXX) for researchers who meet the criteria for access to confidential data.*<br><br>*The data underlying the results presented in the study are available from (include the name of the third party*	Most relevant data are within the manuscript and its Supporting Information files. Some data is available on the websites of the registries, at URLs that are referenced in the various Supplementary Files, as relevant.

and contact information or URL).<br>• This text is appropriate if the data are owned by a third party and authors do not have permission to share the data.<br><br>* typeset	
Additional data availability information:	

Manuscript
Click here to access/download;Manuscript;Manuscript.docx

A comparative analysis of important public clinical trial registries, and a proposal for an interim ideal one [CLEAN COPY]

Nisha Venugopal and Gayatri Saberwal*

Emails: nisha.venugopal2015@gmail.com, gayatri@ibab.ac.in

Authors' affiliation:
Institute of Bioinformatics and Applied Biotechnology
Biotech Park, Electronics City Phase 1
Bengaluru 560100
Karnataka
India

*Corresponding author:
E-mail: gayatri@ibab.ac.in

Keywords: Clinical trial registry, Primary registry, ICTRP, ClinicalTrials.gov, Ethics, Regulatory issues

Abstract

Background

It is an ethical and scientific obligation to register each clinical trial, and report its results, accurately, comprehensively and on time. The WHO recognizes 17 public registries as Primary Registries, and has also introduced a set of minimal standards in the International Standards for Clinical Trial Registries (ISCTR) that primary registries need to implement. These standards are categorized into nine sections — Content, Quality and Validity, Accessibility, Unambiguous Identification, Technical Capacity, Administration and Governance, the Trial Registration Data Set (TRDS), Partner registries and Data Interchange Standards. This study compared the WHO's primary registries, and the US's ClinicalTrials.gov, to examine the implementation of ISCTR, with the aim of defining features of an interim ideal registry.

Methods and Findings

The websites of the 18 registries were evaluated for 14 features that map to one or more of the nine sections of ISCTR, and assigned scores for their variations of these features. The assessed features include the nature of the content; the number and nature of fields to conduct a search; data download formats; the nature of the audit trail; the health condition category; the documentation available on a registry website; and so on. Overall, the registries received between 27% and 80% of the maximum score of 94. The results from our analysis were used to define a set of features of an interim ideal registry.

Conclusions

To the best of our knowledge, this is the first study to quantify the widely divergent quality of the primary registries' compliance with the ISCTR. Even with this limited assessment, it is clear that some of the registries have much work to do, although a mere dozen improvements would significantly improve them.

Introduction

The first two calls for clinical trial registries were made in the 1970s [1]. One aimed to enhance the enrolment of patients in ongoing trials, and the other to reduce the possibility of bias in the subsequent reporting of trial results, caused by the selective publication of those with positive outcomes. Since the year 2000, trial registries have proliferated. Nevertheless, it has been a long and sustained battle by many stakeholders – activists, journals, researchers, funders, governments and the World Health Organization (WHO) – to ensure that large numbers of trials are registered [2–4]. Although the initial two aims for setting up registries continue to be among the most important uses of such databases, researchers have utilized the data in at least a dozen other ways, such as (i) analyzing the conditions, the medical interventions, the sponsors and so on of Expanded Access Studies registered in the United States (US) [5]; (ii) identifying the fraction of trials that have run in the country, that had industry involvement [6]; (iii) conducting a geo-temporal analysis of the trials of novel stem cell therapies [7]; (iv) obtaining information about a trial that was not reported in the subsequent publication [8]; and (v) identifying trials being run in contravention to the law [9]. Given these numerous and diverse purposes, not initially envisaged, it is even more important that all trials are registered and reported in a timely fashion, and that all the data in each record is complete, reliable and readily accessible. In view of this, the quality of data in the databases has long been the subject of analysis and comment. These include (a) analyses of

the quality of registration and missing information in trial records [10–13], (b) studies on the discrepancies in trial status for trials that are registered in more than one registry [14], and (c) reports on the phenomenon of hidden duplicates [15,16]. Other studies have looked into the challenges faced, and advances made by individual registries [17,18].

Certain high profile scandals [19,20] resulted in numerous calls to increase transparency in clinical trials and to improve the public's trust in the trials enterprise. Following the Ministerial Summit on Health Research that took place in Mexico City in November 2004, the WHO launched the International Clinical Trials Registry Platform (ICTRP) initiative in 2006 [21].

The ICTRP enables a single point of access to information regarding trials within its registry network [22], which hosts trial records from around the world. The network consists of (i) Primary Registries (PRs), (ii) Data providers, and (iii) Partner registries [23]. There are currently 17 PRs. The Data providers include the PRs and ClinicalTrials.gov (CTG), of the United States (US). All data providers need to fulfil the same criteria in terms of data collection and management. The two partner registries (i) are not required to fulfil the criteria that PRs need to; (ii) need to be affiliated with one of the PRs; and (iii) cannot directly feed data into the ICTRP [24]. Therefore, we have not included the these two registries in our study. We have analyzed the 17 PRs, and CTG, and refer to them, collectively, as Primary Registries Plus, or PR+**.**

The WHO also developed the International Standards for Clinical Trial Registries (ISCTR) [25], which lists the minimum, and sometimes ideal, standards that PRs should adopt to ensure a basic quality of data and accessibility. These standards are in nine sections — Content; Quality and Validity; Accessibility; Unambiguous Identification; Technical Capacity; Administration and Governance; the 24-field Trial Registration Data Set (TRDS);

Partner Registries; and Data Interchange Standards. Although, ICTRP stipulates that non-compliance with these standards can result in revoking of Primary Registry status, we are unaware of any case where this has happened.

It is known that users trust public registries more than those created by companies or patient groups [26]. Also, public registries are often the primary sources on which other databases are built [26]. It follows that the information in each one should be comprehensive, high quality and available in a user-friendly fashion. Accordingly, there have been calls for (i) a comparison of such registries, to help develop suitable standards [25], and (ii) ways to improve the accessibility and content of the PR+ [27]. However, several years ago it was shown that there had been non-compliance with the WHO minimal dataset [28], and non-optimal website functionality and user experience[10,11,29]. Since across-the-board improvements have not taken place, this issue needs to be reiterated. However instead of undertaking a purely qualitative assessment, we drew inspiration from other researchers' scorecards. These scorecards have either been developed [30–33] or proposed [34,35] to track whether trialists register their studies and report the results accurately, comprehensively and on time. Accordingly, we developed one to assess the PR+.

We have developed the Registries' Comparative Scorecard (the Scorecard) which rates the PR+ on certain features that map to different sections of the ISCTR (S1 Table). We then define an interim 'ideal registry' based on the best variations of each feature used by the PR+. Until such time as all the registries adopt all the standards recommended by ISCTR, the adoption of the recommended variations of each feature would be very helpful for users.

Methods

Data collection

We accessed the websites of the 18 PR+ between July 2019 and April 2020, inclusive. The registries were evaluated for 14 features that map to one or more of the nine sections of ISCTR mentioned above. The list of features was compiled by the authors based on literature regarding the necessity of higher quality trial registrations [36,37], focusing on the standards listed in ISCTR [25].

All information was obtained from one or more of the following resources within each PR+ website: (i) the general pages of the site; (ii) a randomly chosen, sample interventional trial that was registered after 1 January 2019; (iii) supporting documents, if available; and (iv) where necessary and possible, via a login to do a mock registration. All analyses were performed by one author (NV) and verified by the other (GS), with differences resolved by discussion.

The sections below provide further methodological details on the data collection for, and analysis of, each of the features analysed, which have been classified based on the ISCTR section they map to. Reference URLs are available in the Supplementary files, which are referenced in the Results as relevant.

I. Accessibility features

We first examined the accessibility of information in the PR+. For this, we assessed several features, as follows:

(i) The ease of obtaining the total number of trials hosted by the registry:

The method of obtaining the total number of trials hosted by each PR+ was determined. Specifically, we documented (a) whether the number was displayed on the home page, (b) if it was available after a search, or (c) whether it had to be calculated based on the number of pages of results. If there was discrepant information at different places on the site, this fact was captured.

(ii) The existence of a Basic search function:

We examined the presence of the search function using a basic search field.

(iii) and (iv) The number of TRDS fields, and extra fields, that can be used to conduct a search:

We documented the presence and number of (iii) TRDS fields; and (iv) Extra fields, beyond these 24 TRDS fields, that can be used to conduct a search.

(v) The data download options:

For each PR+, we documented the file formats that are available for data download. We also captured information on whether the data on (a) one, (b) a limited number, (c) multiple, or (d) all search results can be downloaded at a time.

II. Content or compliance with TRDS features

(i) TRDS fields and Extra fields:

Each registry provides information about a trial in two different 'views'. While conducting a search, the user first obtains a list of trials which contains the titles, and may also contain other information. This is called the Brief view. Each trial record is available as a Brief view, and a Detailed view. The fields available in these views in each registry were documented. This information was then mapped to the 24 fields of the WHO TRDS. All

additional fields were categorized as ‘Extra fields’.

Among the Extra fields, we looked into the following features in further detail.

(ii) Whether the Principal Investigator (PI) name is compulsory:

Even though the ISCTR states that the PI is the ‘Contact for Scientific Queries’, unless the PI delegates this task to somebody else, the PR+ have not uniformly adopted this definition, and it is not always clear if the ‘Contact for Scientific Queries’ reflects th the PI. Therefore, we have separately looked into whether the PI name specifically, is compulsory.

(iii) The audit trail of each record:

We wished to know whether, where relevant, a given trial

(a) has an audit trail, and if so, (b) whether the changes are clearly highlighted; and (c) whether two versions of the record can be readily compared. In some cases where the sample trial, used for most analyses, did not have a history of changes, we used another sample trial, whose URL is provided in S5 Table.

(iv) The flagging of retrospectively registered trials:

We documented whether each PR+ specifically mentions the registration status of the trial (prospective vs retrospective), or flags retrospectively registered trials.

(v) The reason for the termination of a trial, if applicable:

In this case, we first determined whether the PR+ have a category of terminated trials. For those that do, we captured whether or not a reason for trial termination is provided.

III. Quality and Validity, Technical Capacity, and Data Interchange Standards features

(i) Use of a controlled vocabulary for the health condition category:

We evaluated whether (a) there is a drop down menu for choosing a term from a controlled vocabulary, (b) the registry recommends a widely used controlled vocabulary, or (c) the trialist has to use a free text box.

(ii) The availability of documentation for the processes of the registry, or information on the site:

We evaluated the presence of three types of documents, that is (a) a glossary or the definition of each field of the record, (b) a list of frequently asked questions (FAQs) and (c) one or more user guides.

(iii) Security features of the registry website:

The websites were checked for the presence of a basic security feature, an SSL certificate, as reflected in an 'https' in the website URL, instead of an 'http'.

Box 1. Terminology used in the study

Here, we list a few terms that have been used throughout the study, along with a description of what these refer to:

(a) the word 'section' only refers to one or more of the nine sections of the ISCTR;

(b) the word 'feature' only refers to one or more of the 14 features of each registry that are the focus of this study; and

(c) since the different registries may have different variants of each 'feature', we use the word 'variation' in this context.

The Scorecard

Overall, 14 features of the PR+ were assessed. Each registry has a particular variant of a given feature, which may be more useful or less so. A scoring rationale was devised for every feature analysed, based on which each registry received a score for its variation of a given feature. The rationale is described in detail in Table 1, and further details are provided in the Results and Discussion section.

The following general rules were applied for the scoring system. These are illustrated by particular features in Table 1.

(i) If the feature is absent, the registry gets a score of 0. This is illustrated in features 1.2 and 1.5.

(ii) For features with multiple variations, the score ranges from 1 to 5 based on pre-set criteria, as defined in Table 1. This is illustrated in features 1.1 and 2.1.

(iii) For certain features, which involve counts of fields present, the score increases by 1 point per field. This is illustrated in features 1.3 and 2.2.

(iv) In case a registry has multiple possible scores for a particular feature, the highest one is awarded. This is illustrated in feature 1.5.

Table 1. Rationale for score given to each registry for features used to create the Scorecard.
The relevant Supplementary files with further details are also referenced.

Feature analyzed		Rating scale and rationale	Relevant Supplementary file
**1**	**Accessibility**		
1.1	Total number of trials in the registry	Number displayed on home page: 5<br>Number available after a search: 3<br>Number needs to be calculated: 2<br>Discrepant information at different places on the site: 1	S2 Table
1.2	Existence of Basic search function	Presence of a basic search function: 5<br>Absence of a basic search function: 0	S2 Table
1.3	Advanced search function – TRDS fields	Each TRDS field: 1	S2 Table
1.4	Advanced search function – Extra fields	Each extra field: 1, but with a cap of 5 overall, because of the idiosyncratic nature of some of the search possibilities.	S2 Table

1.5	Data download options	Excel/csv/tsv: 5<br>HTML/XML: 2<br>Word/txt/pdf: 1<br>No download options: 0<br>Since all the registries except NTR permit HTML downloads (even if it is not explicitly stated), no registry gets a rating of ‘1’.	S2 Table
2	**Content or TRDS sections**		
2.1	Brief view: TRDS fields	10 or more fields, which are customizable, and wrapping of text: 5<br>10 or more fields, which are customizable, but without wrapping of text: 4<br>A fixed number of fields, that are more than 3: 3<br>Up to 3 fields: 1	S3 Table
2.2	Brief view: Extra fields	Each field: 1 point	S4 Table
2.3	Detailed view: TRDS fields	The number of fields over 20	S3 Table
2.4	Detailed view: Extra fields	Each field: 1 point<br>In this case, the maximum score is dictated by the registry with the maximum number of fields.	S4 Table
2.5	Whether PI name is compulsory	PI name is compulsory: 5<br>It is not clear whether the scientific contact is the PI (regardless of whether or not this information is compulsory): 2<br>There is a field for the PI name, but it is not clear whether the information is compulsory: 2<br>The PI name is voluntary: 0	S5 Table
2.6	Audit trail	Each of the following aspects receives 1 point: (i) the existence of an audit trail; (ii) the changes made are clearly highlighted; and (iii) it is possible to compare any two versions of the record.	S5 Table
**3**	**Other Sections**		
3.1	Health condition	A drop-down menu for choosing a term from a controlled vocabulary: 5<br>A widely used controlled vocabulary is recommended: 3<br>Free text field: 1	S5 Table
3.2	SSL certificate	Website secured with SSL: 3<br>Website not secured with SSL: 0	S5 Table
3.3	Documentation	Provides (a) a glossary or the definition of each field of the record; (b) List of FAQs; (c) One or more user guides: 1 point each. No points are awarded for the quality of these documents.	S5 Table

Results and Discussion

We first documented basic information about each of the registries. The full name of each registry, its acronym, the country where it is based, and the year it was established are provided in Fig.1 and Table 2. Except CTG, the acronyms used for each registry are the official acronyms. All but one of the PR+ were established between 2000 and 2010, inclusive. LBCTR was established in 2019. Eight registries (ANZCTR, ChiCTR, CTG, DRKS, IRCT, ISRCTN, JPRN, and SLCTR) allow trial registrations from all countries, and the rest usually from the country where the registry is based, or from specific countries or

regions. For example, PACTR caters to clinical trials conducted in Africa. On 18 April 2020, the registries cumulatively held 572,901 records, with CTG accounting for 336,444 (59%).

Trials may be registered either prospectively or retrospectively, that is before the enrolment of the first participant or after. Six of the PR+ (CTRI, IRCT, LBCTR, REPEC, SLCTR and TCTR) only allow prospective registration, whereas the remaining accept retrospective as well. Five of the PR+ (EU-CTR, IRCT, PACTR, REPEC and SLCTR) accept only interventional clinical trials, while the remaining may accept others such as observational studies, post marketing surveys or expanded access programs. All the registries use English, and 11 of them display some or all information in another language as well.

Figure legend

Fig. 1:

A. The timeline of establishment of the PR+. Key events related to trial registration are also noted.

B. Number of records per registry as on 18 April 2020. The pie chart shows the distribution of the number of records in each registry. The actual number, and as a percentage of the total, are also provided.

Table 2. An overview of each registry, listing its acronym, full name, country where it is based, countries from where registration is accepted, type of registration allowed, type of study hosted, and language used.

Registry acronym	Registry full name	Country where registry is based	Countries from where registration is accepted	Type of registration allowed	Type of study	Additional language[1]
ANZCTR	Australian New Zealand Clinical Trials Registry	Australia	All countries. However, trials in Australia and New Zealand are prioritized	Prospective, Retrospective[2]	Interventional, Observational	–
ChiCTR	Chinese Clinical Trial Register	China	All countries	Prospective, Retrospective	Interventional, Observational, Others	Chinese
CRIS	Clinical Research Information Service	Republic of Korea	Republic of Korea	Prospective, Retrospective	Interventional, Observational	Korean

CTG[3]	ClinicalTrials.gov	USA	All countries	Prospective Retrospective	Interventional, Observational, Expanded Access	–
CTRI	Clinical Trials Registry - India	India	Other countries in the region which do not have a Primary Registry of their own[4]	Prospective	Interventional, Observational, PMS[5], BA/BE[5]	–
DRKS	German Clinical Trials Register	Germany	All countries	Prospective, Retrospective	Interventional, Observational, Epidemiological, Others	German
EU-CTR	EU Clinical Trials Register (EU-CTR)	The Netherlands	All interventional trials that have at least one centre in the EU and EEA. Certain trials conducted entirely outside these regions.	Prospective. Retrospective if permitted by National Competent Authority of the Member State	Interventional	Older trials may have content in the host country's language
IRCT	Iranian Registry of Clinical Trials	Iran	All countries	Prospective	Interventional	Persian
ISRCTN	International Standard Registered Clinical/soCial sTudy Number	UK	All countries	Prospective, Retrospective[2]	Interventional, Observational	–
JPRN	Japan Primary Registries Network[6]	Japan	All countries	Prospective, Retrospective	Interventional, Observational	Japanese
LBCTR	Lebanon Clinical Trials Registry	Lebanon	Lebanon	Prospective	Interventional, Observational	Brief summary of the study is also available in Arabic
NTR	Netherlands Trial Register	The Netherlands	Trials conducted in Netherlands or involving Dutch researchers.	Prospective, Ongoing studies	Interventional, Observational	Some information may be available in Dutch
PACTR	Pan African Clinical Trials Registry	South Africa	All countries in Africa	Prospective, Retrospective	Interventional	–
ReBEC	Brazilian Registry of Clinical Trials	Brazil	Brazil[4]	Prospective Retrospective	Interventional, Observational	Portugese and Spanish, for some records, and in a limited way
REPEC	Peruvian Clinical Trial Registry	Peru	Peru	Prospective	Interventional	Spanish
RPCEC	Cuban Public Registry of Clinical Trials	Cuba	Cuba[7]	Prospective, Retrospective	Interventional Observational	Spanish
SLCTR	Sri Lanka Clinical Trials Registry	Sri Lanka	All countries	Prospective	Interventional	–
TCTR	Thai Clinical Trials Registry	Thailand	Thailand	Prospective	Interventional, Observational	–

1. All registries are required to be in English. However, some provide content in additional language(s).
2. Retrospective registration is allowed but prospective registration is preferred and encouraged.
3. Except CTG, all the acronyms listed are the official acronyms.
4. For two registries (CTRI, REBEC) the information on the ICTRP portal and on their own websites is discrepant. Upon inspection, the latter sources appear to be correct, and we have described the registries accordingly.
5. PMS: post-marketing surveillance; BA/BE: Bioavailability/Bioequivalence.
6. Common forum for trials from three Japanese registries, that is (UMIN Clinical Trials Registry (UMIN-CTR), Japan Pharmaceutical Information Center Clinical Trials Information (JAPIC-CTI), and Japan Medical Association - Center for Clinical Trials (JMACCT)).
7. Trials are accepted from Cuban sponsors, conducting trials in Cuba or abroad, with Cuban or foreign products.

Notably, some registries (ChiCTR, EU-CTR, ISRCTN, PACTR, REPEC) were built on earlier versions.

We then analysed 14 features of the PR+, which have been grouped according to the sections of ISCTR that they map to (S1 Table). In Table 3, we list the score obtained by each PR+ per feature, and overall. We also provide the maximum score possible per feature. Further details are provided below, or are available in relevant Supplementary files, which are referenced in Table 1.

Table 3. The Scorecard.
The list of features used to create the Scorecard; the maximum score per feature; the score obtained by each registry per feature, and overall per section; the total score per registry; and the rank of each registry.

		Max score	ANZCTR	ChiCTR	CRIS	CTG	CTRI	DRKS	EU-CTR	IRCT	ISRCTN	JPRN	LBCTR	NTR	PACTR	ReBEC	REPEC	RPCEC	SLCTR	TCTR
**1**	**Accessibility section**																			
1.1	Total number of trials in the registry	**5**	3	3	5	5	3	3	5	5	3	3	1	3	3	5	3	2	2	3
1.2	Existence of Basic search function	**5**	5	0	5	5	5	5	5	5	5	5	0	5	5	5	5	5	0	5
1.3	Advanced search function – TRDS fields	**24**	11	17	14	15	10	8	7	17	13	0	9	0	12	4	1	5	2	7
1.4	Advanced search function – Extra fields	**5**	1	5	5	5	4	5	3	5	5	0	0	0	2	1	2	0	0	0
1.5	Data download options	**5**	5	2	2	5	2	5	2	2	5	5	2	0	2	2	2	2	2	2
	**SUB-TOTAL**	**44**	**25**	**27**	**31**	**35**	**24**	**26**	**22**	**34**	**31**	**13**	**12**	**8**	**24**	**17**	**13**	**14**	**6**	**17**
**2**	**Content or TRDS sections**																			
2.1	Brief view: TRDS fields	**5**	3	3	4	5	3	3	3	3	1	3	3	1	3	3	3	1	1	5
2.2	Brief view: Extra fields	**5**	3	1	2	2	0	3	1	3	1	1	1	0	3	0	0	1	5	1

2.3	Detailed view: TRDS fields	**4**	4	4	3	4	3	2	2	2	4	4	4	3	4	0	3	1	4	3
2.4	Detailed view: Extra fields	**15**	10	5	10	15	8	6	9	7	10	0	9	5	5	4	10	6	5	9
2.5	Whether PI name is compulsory	**5**	5	5	5	0	0	5	5	2	2	2	2	2	5	0	2	5	2	2
2.6	Audit trail	**3**	1	1	3	3	1	3	0	3	2	0	1	0	3	0	0	3	2	0
	**SUB-TOTAL**	**37**	**26**	**19**	**27**	**29**	**15**	**22**	**20**	**20**	**20**	**10**	**20**	**11**	**23**	**7**	**18**	**17**	**19**	**20**
**3**	**Other sections**																			
3.1	Health condition	**5**	5	3	5	3	5	5	5	3	1	5	5	1	5	5	3	1	1	3
3.2	SSL certificate	**5**	5	0	5	5	0	5	5	5	0	5	0	5	5	0	5	0	5	5
3.3	Documentation	**3**	3	2	2	3	3	3	3	1	3	0	3	0	2	3	2	2	2	3
	**SUB-TOTAL**	**13**	**13**	**5**	**12**	**11**	**8**	**13**	**13**	**9**	**4**	**10**	**8**	**6**	**12**	**8**	**10**	**3**	**8**	**11**
	**TOTAL**	**94**	**64**	**51**	**70**	**75**	**47**	**61**	**55**	**63**	**55**	**33**	**40**	**25**	**59**	**32**	**41**	**34**	**33**	**48**
	**% of TOTAL**		**68**	**54**	**74**	**80**	**50**	**65**	**59**	**67**	**59**	**35**	**43**	**27**	**63**	**34**	**44**	**36**	**35**	**51**
	**Rank of each registry**		**3**	**9**	**2**	**1**	**11**	**5**	**7**	**4**	**7**	**15**	**13**	**18**	**6**	**17**	**12**	**14**	**15**	**10**

1. Accessibility:

One of the principal reasons for the existence of clinical trial registries is to provide the public with information, and to thereby increase trust in the trial enterprise [28]. Therefore, we first examined the accessibility of information in the PR+. For this, we assessed several features, as described below:

(i) Ease of obtaining the total number of trials

As a first step, it is important to know how many records the database holds. This number should be readily available, and we have therefore analysed the ease of accessing it. The five registries (CRIS, CTG, EU-CTR, IRCT and ReBEC) that list it on the homepage were given the highest score of 5. Ten registries display this number after a search for all trials, and received a score of 3. Two registries (RPCEC, SLCTR), for which the number of records is available only by a manual calculation, received 2. LBCTR provides discrepant information at different places on the site, and thus received the lowest score of 1. The median score obtained was 3. It is a trivial task to put the figure for the total number of trials on the home page, and we encourage all registries to do so

For a significant fraction of users, the search functionsse are crucially important to access the information in a registry. ISCTR recommends that at the minimum, there must be a basic text search, as well asand it must be possible to searches within the interventions and conditions fields. Several PR+ go much further than this, and therefore we have conducted a detailed assessment of their search capabilities.

(ii) Basic Search function

We determined the presence of a basic search function and have awarded a score of 5 to the 15 registries that provide it. Only three (ChiCTR, LBCTR and SLCTR) do not have this feature, and received 0. The median score was 5.

Most PR+ have a basic search function that enables search by keywords. This is a crucial aspect of the functionality of the trial registry website, and significantly increases the ease of searching for information and improves user experience.

(iii) Advanced search function - TRDS fields

We then examined how many of the 24 TRDS fields could be used in the Advanced search function. Out of a possible score of 24, where the registries received 1 point per field, the maximum score of 17 was attained by ChiCTR and IRCT. JPRN and NTR do not allow a search by any TRDS field and received 0. The remaining registries received scores between 1 and 15. The median score was 8.5.

(iv) Advanced search function - Extra fields

A few registries list fields other than the TRDS fields as part of the search function. Six PR+ (ChiCTR, CRIS, CTG, DRKS, IRCT and ISRCTN) have five or more Extra fields, and therefore received a score of 5. Six registries (ANZCTR, CTRI, EU-CTR, PACTR, ReBEC and REPEC) received scores ranging from 1–4. Six registries do not allow a search using any Extra fields, and received 0. The median score was 2.

Overall, the PR+ provided more fields in the Advanced search function than the minimum recommended by the ISCTR. This becomes especially relevant for researchers conducting systematic reviews, work which requires extensive searches to gather information on clinical trials in specific areas.

(v) Data download options

Having conducted a search, users may wish to download many fields of data, for many records. We therefore gave the highest score of 5 to the five registries (ANZCTR, CTG, DRKS, ISRCTN and JPRN) that allow data downloads in a csv, excel or tsv format. 12 registries provide HTML and XML options, and received a score of 2. Only NTR lacks any options for data download, and received 0. The median score was 2.

All the available data download options are adequate for the inspection of a few records, but it is essential that the PR+ provide bulk data download options such as csv, especially as an increasing number of users are shifting towards automated systems of analysis.

2. Content and TRDS sections

Next, we examined multiple features that map to the Content or TRDS sections, which overlap since the TRDS fields are a form of content. Below, we describe our scoring of the Brief and Detailed views of the PR+.

(i) Brief View: TRDS fields

Since the Brief view is primarily designed to provide an overview of the trial, it can be very helpful for a user if the number of fields in the Brief view can be customized. Therefore, we have given higher scores to registries that provide this option. Two registries (CTG and TCTR), display more than 10 TRDS fields, and allow customization and text wrapping. They received the maximum score of 5. CRIS displays more than 10 TRDS fields, that are customizable but without text wrap, and received 4. Eleven registries display more than three fields, which are fixed, and got a score of 3. The four registries (ISRCTN, NTR, RPCEC and SLCTR) that display three fields or less received 1. The median score was 3.

A customizable brief view of search results is extremely useful in a trial registry, where different types of users such as patients, healthcare professionals or sponsors, may be interested in different fields.

(ii) Detailed View: TRDS fields

The Detailed view tends to have all 24 TRDS fields. However, we found that all the PR+ do not yet list the four fields that have been included in the latest version of TRDS [29]. Eight registries (ANZCTR, ChiCTR, CTG, ISRCTN, JPRN, LBCTR, PACTR and SLCTR) do so, and received the highest score of 4. Most of the remaining PR+ display between one and three of the new fields and were scored accordingly. Only one registry, ReBEC, has not been updated to display any of the new fields, and received a score of 0. The median score was 3. We hope that over time more registries will be in full compliance with the ISCTR-mandated fields.

(iii) Extra fields

Registries list Extra fields in both the Brief and Detailed views. In the Brief View, only SLCTR received the maximum score of 5. Four registries (CTRI, NTR, ReBEC and REPEC) have no Extra fields and received a score of 0. The remaining have between one and three of such fields and were scored accordingly. The median score was 1.

In the Detailed View, most registries have between five and 10 Extra fields. However CTG has 15, and JPRN has none. The median score was 7.5.

Some of the Extra fields, such as the date of last update, and whether registration was prospective or retrospective, are recommended by ISCTR. Interestingly, one-third or more of the registries list several fields that the ISCTR does not specifically recommend. This seems to reflect a certain level of agreement among the managers of registries that particular fields

are important. There may be a range of reasons for including these fields. For instance, India had been criticized for the lack of appropriate oversight to ensure the ethical conduct of trials, and therefore CTRI asked trialists for details of the ethics committee even before ISCTR required this information [38,39]. Also, there have been demands from the Cochrane collaboration, and many other individuals and groups, to include several additional items in the ISCTR list, which WHO has not agreed to. It is alleged that the recommended list is closer to what industry demanded [40]. As such, although ISCTR may not list every field that many people believed to be essential, managers of particular registries may have chosen to list some of them.

We explored some of these Extra fields in greater detail below.

(iv) Whether PI name is compulsory

For the sake of accountability it is important that the field 'PI name' is compulsory [25,41]. Although we have assessed Contact for Scientific Queries as a TRDS field, we have not assumed that this person is the PI, and therefore have separately looked into whether the PI name is compulsory. In seven registries (ANZCTR, ChiCTR, CRIS, DRKS, EU-CTR, PACTR and RPCEC) it is so, and they received the highest score of 5. Several have either not made it clear whether the scientific contact is the PI, or have a separate field for the PI name but have not stated whether it is compulsory. They each received a score of 2. Three registries (CTG, CTRI and ReBEC) have marked this field as voluntary, and received 0. The median score was 2.

WHO documents [25,41] have contradictory information on the issue of PI name and Contact for Scientific Queries. They require that the PI's name, title and email ID be provided, but state that this should be a functional name, not a personal one. ISCTR states that the PI is the Contact for Scientific Queries, unless the PI delegates this task to somebody else. If the PI

name is compulsory – and preferably recorded in a fixed format [42] – then this information will enable researchers to quantify the number of unique PIs in a country, ask whether a PI has been taking on too many trials, and perform other analyses. Therefore we commend the registries that have made this field compulsory.

(v) Audit trail

ISCTR requires that the audit trail of each record should be publicly available and so we have examined the presence and usefulness of this feature. Six registries (CRIS, CTG, DRKS, IRCT, PACTR and RPCEC) have the option of comparing two versions of the trial record and received the maximum score of 3; two (ISRCTN and SLCTR) have highlighted the changes made to a trial record, and got 2; four (ANZCTR, ChiCTR, CTRI and LBCTR) have a basic form of an audit trail and got 1; and six of the PR+ do not provide any audit trail and got 0. The median score was 1. It is clear that most registries do not have an ideal audit trail.

The information pertaining to the following two features is present in the Extra fields, either as a separate field in the Detailed view, or marked with a flag in the Brief View. Hence we have not scored these features separately.

(vi) Flagging retrospective or prospective registration status of a trial

Prospective registration is crucial to prevent unrecorded ‘outcome switching’, which creates a bias in the medical evidence base [28]. Nevertheless, it has been argued that (i) it is a duty to trial participants to register each trial, and subsequently publish the results, and (ii) not registering a trial could lead to its loss from the documented universe of trials [43].
As such, retrospective registration is better than non-registration, and therefore many PR+ permit it. We have documented this in Table 1.

Users may have more confidence in the results of a prospectively than a retrospectively registered trial. Further, flagging retrospectively registered ones may shame the registrants into registering prospectively in future [14]. Accordingly, we have analysed whether PR+ highlight the registration status of trials and flag retrospectively registered ones.

Over half of the PR+ do so.

(vii) The reason for the termination of a trial, if applicable

It is important to know why a study was terminated as it provides economic, ethical and scientific insights that can help improve ongoing or upcoming clinical trials [44]. Our analysis showed that only eight registries provide this information at all, and only three provide drop-down menus of reasons for termination (Table S5). Researchers who have studied the leading causes of trial termination have suggested that the cause should be selected from a fixed set of options [45].

3. Other sections:

Finally, we examined three features that map to other sections of the ISCTR, as follows: (a) Health condition, (b) the presence of a Secure Sockets Layer (SSL) certificate and (c) Documentation.

(i) Health condition

First, the issue of classifying health conditions, which maps to Data Interchange Standards. We find that only half the PR+ provide drop-down menus for this field, and they received the highest score of 5. Five registries (ChiCTR, CTG, IRCT, REPEC and TCTR) recommend the use of standardized vocabulary, and received 3. Four registries (ISRCTN, NTR, RPCEC and SLCTR) that do not provide such options, and have a free text field for health condition, received 1. The median score was 4.

Comparisons across registries are easier if each one uses a controlled vocabulary, and in particular one that maps to a widely-used metathesaurus [46] as recommended in ISCTR [25]. It is therefore preferable that the health condition be selected from a fixed set of options.

(ii) The presence of an SSL certificate

Second, the security of the website. In the Technical Capacity section, ISCTR requires that each registry have adequate protection against the corruption or loss of data. We have assessed something basic, that is whether the website is secured with an SSL certificate, as is evident when a website URL contains 'https'. We find that only 12 of the PR+ websites have this certification, and each received a score of 5. The remaining six registries (ChiCTR, CTRI, ISRCTN, LBCTR, ReBEC and RPCEC) have URLs with an 'http', and received 0. The median score was 5.

The SSL certificate is an important tool to safeguard data of the registry and that of its users, and Google currently marks all sites without it as insecure [47]. As such, it is also a sign of credibility for a user who may hesitate to access a site that lacks a security certificate.

(iii) Documentation

Third, the issue of documentation. Various documents help users to understand the processes of a registry, or the data it hosts. Only half the PR+ provide all the three types of documentation we have assessed, and received a score of 3. Six registries (ChiCTR, CRIS, PACTR, REPEC, RPCEC and SLCTR) have only two types of documents and received 2, and one registry (IRCT) displays only a user guide and received a score of 1. Two registries (JPRN and NTR) do not provide any documentation, and have received 0. The median score obtained was 2.5.

Although the three documents that we have scored are not explicit requirements of the ISCTR, they assist users in registering their trial correctly. As such, this feature maps to the Quality and Validity section.

In general, we have barely touched upon Quality and Validity, since investigating the completeness or quality of the records in the PR+ would be a large exercise in itself. The sponsor needs to ensure a high quality trial record, but this may not happen, and various studies have highlighted deficiencies in the records of different registries [10–13,48]. It is also the duty of the managers of the registry to facilitate better quality trial registration, as has been recommended by ISCTR. Additionally, for several of the minimum standards recommended by ISCTR, either it is not possible for us to assess compliance, or the requirements do not immediately impact use of the registry data. Therefore we have also barely touched upon Unambiguous Identification (although Secondary identifying numbers, a field in TRDS, also maps to this section), Technical Capacity, and Data Interchange Standards. Further, we have not touched upon the sections (i) Administration and Governance, and (ii) Partner Registries.

Overall, as derived from an assessment of 14 features described above, the maximum score that any registry obtained was 94 points (Table 3). The PR+ received scores ranging from 27% (NTR) to 80% (CTG) of the maximum, with an average of 52%. Despite the limited nature of our audit, the lowest- and highest-scoring registries received scores that differ by over 50%. To the best of our knowledge, this widely divergent quality of the PR+ has not been documented before.

An ideal registry

We found that the registries show a high degree of variability for a given feature, ranging from a sophisticated to a routine variation, or its complete absence. We have used the best variant of the features analyzed to define an interim ideal registry. In such a registry,

(i) the total number of trials is displayed on the home page;

(ii) a search is possible through (a) a basic search function, (b) each of the TRDS fields, and (c) a few extra fields;

(iii) the data download options include a csv, excel, or tsv format, and support automated bulk downloads;

(iv) the Brief view is customizable, with 10 or more fields, with text wrapping;

(v) the Detailed view includes all the TRDS fields;

(vi) there is clarity on whether or not the scientific contact is the PI;

(vii) the PI name is compulsory;

(viii) the reason for the termination of a trial is selected from a drop-down menu of possible reasons;

(ix) each trial has an audit trail that enables a comparison of any two versions;

(x) at the very least, the following documents are provided, in English: (a) a definition of each field of the record, (b) a list of FAQs, and (c) one or more user guides;

(xi) the website is secured with an SSL certificate; and

(xii) the health condition category is chosen from a drop-down menu with a controlled vocabulary, preferably a widely used one.

The ISCTR recommends several other standard including higher data quality, more complete records and the reporting of results. Although it is hoped that all registries will implement all of these standards in due course, in the interim, registries may wish to implement the list above if they have not already done so.

Registries have many users. The scorecard above analyses features that are of interest to the authors and, by extension, possibly to other researchers concerned with the health of the trial ecosystem overall. Other categories of users, such as medical professionals, patients, trial sponsors, policy makers, data scientists and so on, may wish to alter the assessed features, or the scoring, in order to rank the registries according to their priorities. For instance, a data scientist would be very appreciative of ANZCTR, which specifically enables web crawling of its records [49]. Furthermore, the managers of other registries, either public or private, and either based on the data in the PR+ or not, may be interested in the results of this study.

The ongoing Covid-19 pandemic has forcefully brought home the need for high quality trial registries with information that is consistent, comprehensive and available in a user-friendly fashion. Billions of people need to be immediately protected from the virus, and large numbers of drugs and vaccines are in trials. There is world-wide interest in these trials, and information that is being tracked includes what is being trialled; where these trials are taking place; and the results of these trials. Each country needs to take public health decisions, which will evolve as the evidence from trials running in different parts of the world yield results. Public trial registries are one of the fastest ways of communicating these results.

Further, the publicly available, freely accessible information in such registries helps to build trust with the public [26,44]. Covid-19 trials have been among the fastest recruiting ones in history [50,51], and it is possible that the publicly available information in trial registries has helped many of the potential trial participants decide to enrol.

It is not just that everyone is interested in the positive outcomes of trials. For example, an inspection of the CTRI records of hundreds of Covid-19 trials being run in India has thrown up quality issues in almost all of them. Based on negative publicity, the government has taken action in some cases [52].

There is a long history of various stakeholders arguing for the need to improve registries and the quality of trial registration. Examples include academics and health activists [53–55], journals (ICMJE) [56], WHO [41], registry managers [57], funders [58,59] and governments [60]. Each of these efforts has led to some improvements in the number and quality of trial records hosted by registries. However none of them has led to a perfect set of records. It is likely that the only way this will be achieved is if all stakeholders continue to apply pressure on the registries. Studies such as this one help to highlight deficiencies, which adds to the other efforts aimed at improving registries. The authors would welcome other researchers' efforts to create and update a website that lists the scorecard, with periodic updates. Should such a website not be created by any other group, the authors intend to re-evaluate the registries' performance on the scorecard every few years.

In summary, to the best of our knowledge, this is the first study undertaking a comparative analysis of WHO-recognized registries to assess compliance to ISCTR. Our use of a scorecard, based on preset criteria, ensured an impartial quantification of the quality of the features analyzed across the PR+. As such, even though our study analyzed a limited set of features, it clearly shows the substantial variation in compliance with the recommended minimal standards. Our study would be helpful to researchers who may wish to extend this audit and evaluate the completeness of the records or the quality of their data, two other major issues, in all 18 registries.

This study has a few limitations, as follows: (i) It assesses only some of the many features in each registry. In particular, it does not evaluate any aspect of trial methodology or results, which are crucial portions of such registries. As such, otherwise outstanding registries may have fared less well than expected. (ii) We have not evaluated the completeness of any records or the quality of their data. (iii) Each registry has been evaluated with respect to the

list of fields in a recently registered trial. Earlier records in the same registry may have different content if the required details have changed over time. (iv) We have primarily focused on information that is available in English and may have missed important content in other languages. (v) Although applied systematically, the absolute values of the scores are arbitrary

Conclusions

Over the years, CTG has received most of the attention of those interested in the accessibility and integrity of the data in public trial registries. As noted above, 41% of the records are held in the other PR+, and need to be examined as well. We have identified the best variations of several features that have already been implemented by one or more of these registries, and which serve as pointers on how the others may improve. Running a registry is not merely a bureaucratic task, but is part of a mission to safeguard patients' lives, and the ethics and science of medicine. We hope that our analysis is of some assistance in this.

References

1. Dickersin K, Rennie D. Registering clinical trials. JAMA. 2003 Jul 23;290(4):516-23. doi: 10.1001/jama.290.4.516. PMID: 12876095.

2. Krleza-Jerić K. Clinical trial registration: the differing views of industry, the WHO, and the Ottawa Group. PLoS Med. 2005 Nov;2(11):e378. doi: 10.1371/journal.pmed.0020378. Epub 2005 Oct 18. PMID: 16221000; PMCID: PMC1255765.

3. Lundh A, Krogsbøll LT, Gøtzsche PC. Access to data in industry-sponsored trials. Lancet. 2011 Dec 10;378(9808):1995-1996. doi: 10.1016/S0140-6736(11)61871-0. PMID: 22153200.

4. Lemmens T and Bouchard R, Mandatory Clinical Trial Registration: Rebuilding Public Trust in Medical Research (2007). GLOBAL FORUM UPDATE ON RESEARCH FOR HEALTH, Vol. 4: Equitable Access: Research Challenges for Health in Developing Countries, pp. 40-46, London: Pro-Book Publishing, 2007. Available at SSRN: https://ssrn.com/abstract=1083565.

5. Miller JE, Ross JS, Moch KI, Caplan AL. Characterizing expanded access and compassionate use programs for experimental drugs. BMC Res Notes. 2017 Jul

28;10(1):350. doi: 10.1186/s13104-017-2687-5. PMID: 28754150; PMCID: PMC5534121.

6. Askie LM, Hunter KE, Berber S, Langford A, Tan-Koay AG, Vu T et al. The clinical trials landscape in Australia 2006–2015. Sydney: Australian New Zealand Clinical Trials Registry 2017.

7. Li MD, Atkins H, Bubela T. The global landscape of stem cell clinical trials. Regen Med. 2014 Jan;9(1):27-39. doi: 10.2217/rme.13.80. Epub 2013 Nov 18. PMID: 24236476.

8. Tharyan P, George AT, Kirubakaran R, Barnabas JP. Reporting of methods was better in the Clinical Trials Registry-India than in Indian journal publications. J Clin Epidemiol. 2013 Jan;66(1):10-22. doi: 10.1016/j.jclinepi.2011.11.011. Epub 2012 Mar 27. PMID: 22459428.

9. Yasmeen A. Infant milk: Nestle's clinical trial under scanner. The Hindu. 3 Aug 2019. Available: https://www.thehindu.com/news/national/infant-milk-nestles-clinical-trial-under-scanner/article28809296.ece. Accessed 25 Sep 2020.

10. Viergever RF, Ghersi D. The quality of registration of clinical trials. PLoS One. 2011 Feb 24;6(2):e14701. doi: 10.1371/journal.pone.0014701. PMID: 21383991; PMCID: PMC3044717.

11. Viergever RF, Karam G, Reis A, Ghersi D. The quality of registration of clinical trials: still a problem. PLoS One. 2014 Jan 10;9(1):e84727. doi: 10.1371/journal.pone.0084727. PMID: 24427293; PMCID: PMC3888400.

12. Chaturvedi N, Mehrotra B, Kumari S, Gupta S, Subramanya HS, Saberwal G. Some data quality issues at ClinicalTrials.gov. Trials. 2019 Jun 24;20(1):378. doi: 10.1186/s13063-019-3408-2. PMID: 31234923; PMCID: PMC6591874.

13. Pillamarapu M, Mohan A, Saberwal G. An analysis of deficiencies in the data of interventional drug trials registered with Clinical Trials Registry - India. Trials. 2019 Aug 28;20(1):535. doi: 10.1186/s13063-019-3592-0. PMID: 31455366; PMCID: PMC6712861.

14. Fleminger J, Goldacre B. Prevalence of clinical trial status discrepancies: A cross-sectional study of 10,492 trials registered on both ClinicalTrials.gov and the European Union Clinical Trials Register. PLoS One. 2018 Mar 7;13(3):e0193088. doi: 10.1371/journal.pone.0193088. PMID: 29513684; PMCID: PMC5841737.

15. Zarin DA, Tse T, Williams RJ, Carr S. Trial Reporting in ClinicalTrials.gov - The Final Rule. N Engl J Med. 2016 Nov 17;375(20):1998-2004. doi: 10.1056/NEJMsr1611785. Epub 2016 Sep 16. PMID: 27635471; PMCID: PMC5225905.

16. Kumari S, Mohan A, Saberwal G. Hidden duplicates: 10s or 100s of Indian trials, registered with ClinicalTrials.gov, have not been registered in India, as required by law. PLoS One. 2020 Jun 19;15(6):e0234925. doi: 10.1371/journal.pone.0234925. PMID: 32559240; PMCID: PMC7304601.

17. Alarcon-Ruiz CA, Roque-Roque JS, Heredia P, Gómez-Briceño AR, Quispe AM. Twenty-two years' experience registering trials in a low-middle income country: The Peruvian Clinical Trial Registry. J Evid Based Med. 2019 Aug;12(3):187-193. doi: 10.1111/jebm.12354. Epub 2019 Jun 18. PMID: 31215157.

18. Ranawaka UK, de Abrew A, Wimalachandra M, Samaranayake N, Goonaratna C. Ten years of clinical trial registration in a resource-limited setting: Experience of the Sri Lanka clinical trials registry. J Evid Based Med. 2018 Feb;11(1):46-50. doi: 10.1111/jebm.12284. Epub 2018 Jan 11. PMID: 29322622.

19. Krumholz HM, Ross JS, Presler AH, Egilman DS. What have we learnt from Vioxx? BMJ. 2007 Jan 20;334(7585):120-3. doi: 10.1136/bmj.39024.487720.68. PMID: 17235089; PMCID: PMC1779871.

20. Jureidini JN, McHenry LB, Mansfield PR. Clinical trials and drug promotion: Selective reporting of study 329. International Journal of Risk & Safety in Medicine. 2008;20: 73–81. doi:10.3233/JRS-2008-0426

21. WHO Technical Consultation on Clinical Trials Registration Standards. Geneva: World Health organization. 25-27 April 2005; Available: https://www.who.int/ictrp/news/ictrp_meeting_april2005_conclusions.pdf

22. ICTRP Search Portal [Internet]. [cited 21 Mar 2020]. Available: http://apps.who.int/trialsearch/

23. WHO | The WHO Registry Network [Internet]. [cited 1 Oct 2020]. Available: https://www.who.int/ictrp/network/en/

24. WHO | Partner Registries [Internet]. [cited 1 Oct 2020]. Available: https://www.who.int/ictrp/network/partner/en/

25. International Standards for Clinical Trial Registries – Version 3.0. Geneva: World Health Organization; 2018. License: CC BY-NC-SA 3.0 IGO

26. The-Clinical-Trial-Registry-of-the-Future-Proposal.pdf [Internet]. [cited 2020 Mar 22]. Available from: http://www.transceleratebiopharmainc.com/wp-content/uploads/2017/11/The-Clinical-Trial-Registry-of-the-Future-Proposal.pdf

27. WHO ICTRP Registry Network Meeting Summary Report. Geneva: World Health Organization; 2015. Available: https://www.who.int/ictrp/news/ICTRP_registry_network_meeting_report_2015_web.pdf?ua=1

28. Moja LP, Moschetti I, Nurbhai M, Compagnoni A, Liberati A, Grimshaw JM, Chan AW, Dickersin K, Krleza-Jeric K, Moher D, Sim I, Volmink J. Compliance of clinical trial registries with the World Health Organization minimum data set: a survey. Trials. 2009 Jul 22;10:56. doi: 10.1186/1745-6215-10-56. PMID: 19624821; PMCID: PMC2734552.

29. Ogino D, Takahashi K, Sato H. Characteristics of clinical trial websites: information distribution between ClinicalTrials.gov and 13 primary registries in the WHO registry network. Trials. 2014 Nov 5;15:428. doi: 10.1186/1745-6215-15-428. PMID: 25373358; PMCID: PMC4234832.

30. Miller JE, Wilenzick M, Ritcey N, Ross JS, Mello MM. Measuring clinical trial transparency: an empirical analysis of newly approved drugs and large pharmaceutical companies. BMJ Open. 2017 Dec 5;7(12):e017917. doi: 10.1136/bmjopen-2017-017917. PMID: 29208616; PMCID: PMC5728266.

31. Goldacre B. How to get all trials reported: audit, better data, and individual accountability. PLoS Med. 2015 Apr 14;12(4):e1001821. doi: 10.1371/journal.pmed.1001821. PMID: 25874719; PMCID: PMC4396123.

32. Our scoring method. [Internet]. [cited 21 Mar 2020]. Available: http://policyaudit.alltrials.net/our-scoring-method/

33. Goldacre B, Gray J. OpenTrials: towards a collaborative open database of all available information on all clinical trials. Trials. 2016 Apr 8;17:164. doi: 10.1186/s13063-016-1290-8. PMID: 27056367; PMCID: PMC4825083.

34. Azar M, Riehm KE, Saadat N, Sanchez T, Chiovitti M, Qi L, Rice DB, Levis B, Fedoruk C, Levis AW, Kloda LA, Kimmelman J, Benedetti A, Thombs BD. Evaluation of Journal Registration Policies and Prospective Registration of Randomized Clinical Trials of Nonregulated Health Care Interventions. JAMA Intern Med. 2019 May 1;179(5):624-632. doi: 10.1001/jamainternmed.2018.8009. PMID: 30855655; PMCID: PMC6503638.

35. Miller J, Ross JS, Wilenzick M, Mello MM. Sharing of clinical trial data and results reporting practices among large pharmaceutical companies: cross sectional descriptive study and pilot of a tool to improve company practices. BMJ. 2019 Jul 10;366:l4217. doi: 10.1136/bmj.l4217. PMID: 31292127; PMCID: PMC6614834.

36. Jones CW, Safferman MR, Adams AC, Platts-Mills TF. Discrepancies between ClinicalTrials.gov recruitment status and actual trial status: a cross-sectional analysis. BMJ Open. 2017 Oct 11;7(10):e017719. doi: 10.1136/bmjopen-2017-017719. PMID: 29025842; PMCID: PMC5652524.

37. Chan AW, Song F, Vickers A, Jefferson T, Dickersin K, Gøtzsche PC, Krumholz HM, Ghersi D, van der Worp HB. Increasing value and reducing waste: addressing inaccessible research. Lancet. 2014 Jan 18;383(9913):257-66. doi: 10.1016/S0140-6736(13)62296-5. Epub 2014 Jan 8. PMID: 24411650; PMCID: PMC4533904.

38. Tharyan P. Ethics committees and clinical trials registration in India: opportunities, obligations, challenges and solutions. Indian J Med Ethics. 2007 Oct-Dec;4(4):168-9. doi: 10.20529/IJME.2007.066. PMID: 18630235.

39. Pandey A, Aggarwal A, Seth S, Maulik M, Bano R, Juneja A. Clinical Trials Registry--India: redefining the conduct of clinical trials. Indian J Cancer. 2008 Jul-Sep;45(3):79-82. doi: 10.4103/0019-509x.44060. PMID: 19018108.

40. Krleza-Jerić K. Clinical trial registration: the differing views of industry, the WHO, and the Ottawa Group. PLoS Med. 2005 Nov;2(11):e378. doi: 10.1371/journal.pmed.0020378. Epub 2005 Oct 18. PMID: 16221000; PMCID: PMC1255765.

41. WHO | WHO Data Set. In: WHO [Internet]. [cited 16 May 2019]. Available: http://www.who.int/ictrp/network/trds/en/

42. Potthast R, Vervölgyi V, McGauran N, Kerekes MF, Wieseler B, Kaiser T. Impact of inclusion of industry trial results registries as an information source for systematic reviews. PLoS One. 2014 Apr 17;9(4):e92067. doi: 10.1371/journal.pone.0092067. PMID: 24743113; PMCID: PMC3990559.

43. Harriman SL, Patel J. When are clinical trials registered? An analysis of prospective versus retrospective registration. Trials. 2016 Apr 15;17:187. doi: 10.1186/s13063-016-1310-8. PMID: 27079379; PMCID: PMC4832501.

44. Committee on Clinical Trial Registries; Board on Health Sciences Policy; Institute of Medicine. Developing a National Registry of Pharmacologic and Biologic Clinical Trials: Workshop Report. Washington (DC): National Academies Press (US); 2006. PMID: 26740992.

45. Williams RJ, Tse T, DiPiazza K, Zarin DA. Terminated Trials in the ClinicalTrials.gov Results Database: Evaluation of Availability of Primary Outcome Data and Reasons for Termination. PLoS One. 2015 May 26;10(5):e0127242. doi: 10.1371/journal.pone.0127242. PMID: 26011295; PMCID: PMC4444136.

46. Metathesaurus. [cited 22 Mar 2020]. Available: https://www.nlm.nih.gov/research/umls/knowledge_sources/metathesaurus/index.html

47. Council Post: Why An SSL Certificate Is Important For Your Company Website. [Internet]. [cited 25 Sep 2020]. Available: https://www.forbes.com/sites/forbestechcouncil/2018/05/18/why-an-ssl-certificate-is-important-for-your-company-website/#2181c3fb1dc3

48. Yadav P, Jaykaran, Chaudhari M, Saxena D, Kantharia ND. Clinical trials registered in clinical trial registry of India: A survey. J Pharmacol Pharmacother. 2011 Oct;2(4):289-92. doi: 10.4103/0976-500X.85953. PMID: 22025862; PMCID: PMC3198529.

49. ANZCTR [Internet]. [cited 25 Sep 2020]. Available: https://www.anzctr.org.au/crawl.aspx

50. Oxford recruits 2,700 virus patients for fast-growing drugs trial. Financial Times. [cited 25 Sep 2020]. Available: https://www.ft.com/content/f4e3055e-72e2-459d-ba66-05af7d2c8915

51. Clinical trials for a Covid-19 vaccine to begin in Rochester. WHAM. [cited 25 Sep 2020]. Available: https://13wham.com/news/local/clinical-trials-for-a-covid-19-vaccine-to-begin-in-rochester

52. COVID-19: Hundreds Of Clinical Trials Under Way In India, Many Lack Rigour, Say Experts. [cited 25 Sep 2020]. Available: https://www.indiaspend.com/covid-19-hundreds-of-clinical-trials-under-way-in-india-many-lack-rigour-say-experts/

53. Krleza-Jerić K, Chan AW, Dickersin K, Sim I, Grimshaw J, Gluud C. Principles for international registration of protocol information and results from human trials of health related interventions: Ottawa statement (part 1). BMJ. 2005 Apr 23;330(7497):956-8. doi: 10.1136/bmj.330.7497.956. Erratum in: BMJ. 2005 May 28;330(7502):1258. PMID: 15845980; PMCID: PMC556346.

54. AllTrials. All Trials Registered. All Results Reported. In: AllTrials [Internet]. [cited 16 May 2019]. Available: http://www.alltrials.net/

55. Miller JE, Korn D, Ross JS. Clinical trial registration, reporting, publication and FDAAA compliance: a cross-sectional analysis and ranking of new drugs approved by the FDA in 2012. BMJ Open. 2015 Nov 12;5(11):e009758. doi: 10.1136/bmjopen-2015-009758. PMID: 26563214; PMCID: PMC4654354.

56. ICMJE | Recommendations | Clinical Trials. [cited 10 Jun 2019]. Available: http://www.icmje.org/recommendations/browse/publishing-and-editorial-issues/clinical-trial-registration.html

57. Zarin DA, Tse T, Williams RJ, Rajakannan T. Update on Trial Registration 11 Years after the ICMJE Policy Was Established. N Engl J Med. 2017 Jan 26;376(4):383-391. doi: 10.1056/NEJMsr1601330. PMID: 28121511; PMCID: PMC5813248.

58. Clinical trials policy – Grant funding | Wellcome. [cited 30 Sep 2020]. Available: https://wellcome.org/grant-funding/guidance/clinical-trials-policy

59. Publishing Policies | Gates Open Research. [cited 30 Sep 2020]. Available: https://gatesopenresearch.org/about/policies#trialreg

60. FDAAA 801 and the Final Rule - ClinicalTrials.gov. [cited 25 Sep 2020]. Available: https://clinicaltrials.gov/ct2/manage-recs/fdaaa

Supporting information

S1 Table. Mapping to ISCTR. The 14 features analyzed in this study map to the following nine sections of ISCTR: (i) Content, (ii) Quality and Validity, (iii) Accessibility, (iv) Unambiguous Identification, (v) Technical Capacity, (vi) Administration and Governance, (vii) The 24-field TRDS, (viii) Partner Registries, and (ix) Data Interchange Standards.

S2 Table. Data on six aspects of each registry. (a) Total number of trials in the registry, (b) Existence of a basic search function, (c and d) Advanced search function – TRDS fields and Extra fields, and (e) Data download options.

S3 Table. The list of the TRDS fields that are present in the Brief view and the Detailed view. The presence or absence of the field is indicated by a 1 or 0, respectively. The number of the sample trial used for each registry is also provided.

S4 Table. For each registry, a listing of the Extra fields in the Brief and Detailed views.

S5 Table. Data on multiple aspects of each registry. (a) whether the PI name is compulsory; (b) reason for the termination of a trial, and whether there is a drop-down menu of reasons; (c) audit trail; (d) health condition (e) SSL certificate, and (f) documentation.

Figure
Click here to access/download;Figure;Fig 1.tif

A

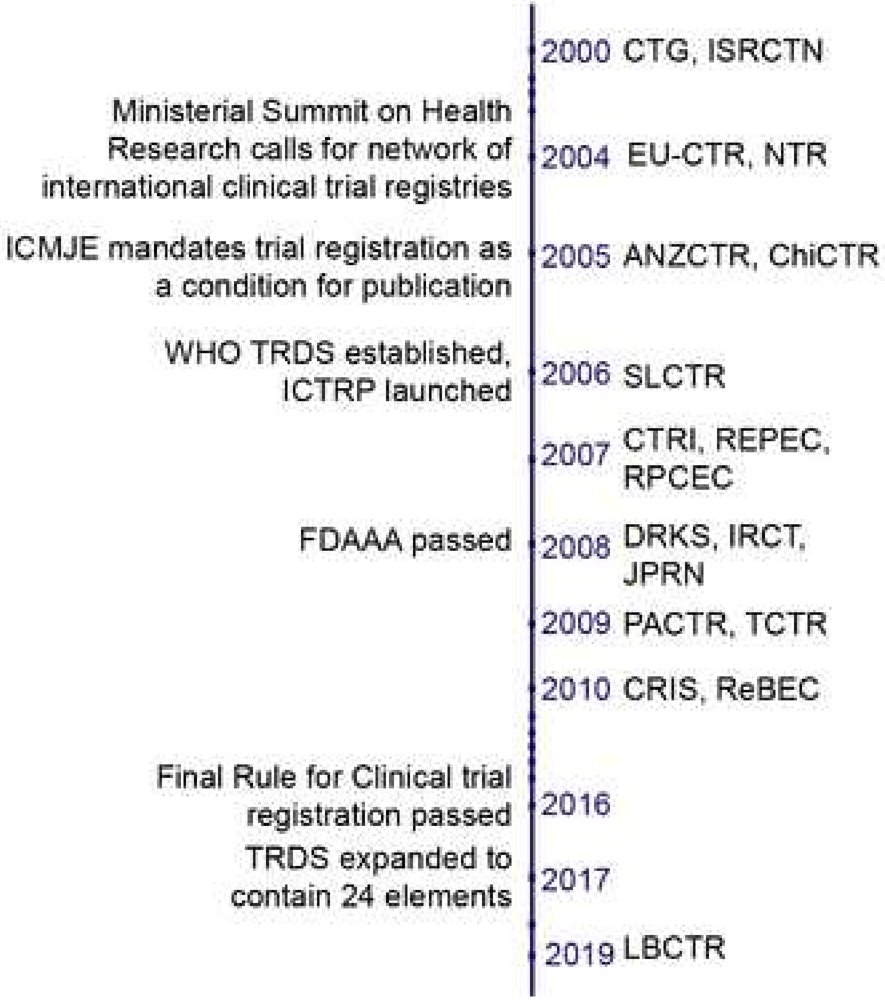

B

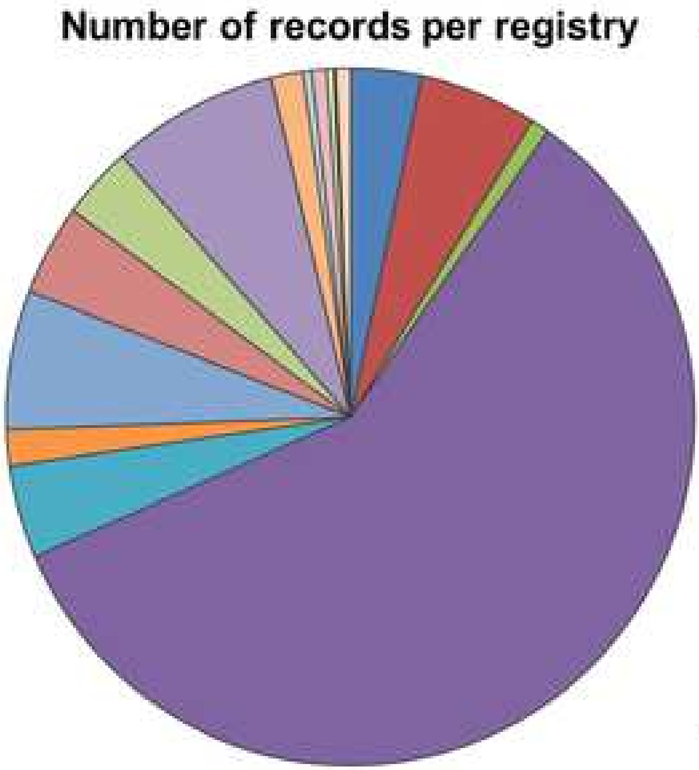

Registry	Number of Records	% of Total
ANZCTR	19150	3.3
ChiCTR	31578	5.5
CRIS	4916	0.9
CTG	336444	58.7
CTRI	24718	4.3
DRKS	9581	1.7
EU-CTR	36915	6.4
IRCT	24117	4.2
ISRCTN	19329	3.4
JPRN	44873	7.8
LBCTR	69	0.0
NTR	8521	1.5
PACTR	2318	0.4
ReBEC	3992	0.7
REPEC	1845	0.3
RPCEC	307	0.1
SLCTR	345	0.1
TCTR	3883	0.7
**Total**	**572901**	**100**

Click here to access/download
Supporting Information
S1 Table.xlsx

Click here to access/download
Supporting Information
S2 Table.xlsx

Click here to access/download

Supporting Information

S3 Table.xls

Click here to access/download
Supporting Information
S4 Table.xlsx

Click here to access/download
Supporting Information
S5 Table.xlsx

A comparative analysis of important public clinical trial registries, and a proposal for an interim ideal one [TRACK CHANGES]

Nisha Venugopal and Gayatri Saberwal*

Emails: nisha.venugopal2015@gmail.com, gayatri@ibab.ac.in

Authors' affiliation:
Institute of Bioinformatics and Applied Biotechnology
Biotech Park, Electronics City Phase 1
Bengaluru 560100
Karnataka
India

*Corresponding author:
E-mail: gayatri@ibab.ac.in

Keywords: Clinical trial registry, Primary registry, ICTRP, ClinicalTrials.gov, Ethics, Regulatory issues

Abstract

Background

It is an ethical and scientific obligation to register each clinical trial, and report its results, accurately, comprehensively and on time. The WHO recognizes 17 public registries as Primary Registries~~.~~, and ~~It~~ has also introduced a set of minimal standards in the ~~(~~International Standards for Clinical Trial Registries ~~, or~~ (ISCTR) that ~~these~~ primary registries need to implement. These standards are categorized into nine sections — Content, Quality and Validity, Accessibility, Unambiguous Identification, Technical Capacity, Administration and Governance, the Trial Registration Data Set (TRDS), Partner registries and Data Interchange Standards. This study compared ~~these~~the WHO's primary registries, and the US's ClinicalTrials.gov, to examine the implementation of ISCTR, with the aim of defining features of an interim ideal registry.

~~Methods and Findings~~

Methods and Findings

The websites of the 18 registries were evaluated for 14~~7~~ features that map to one or more of the nine sections of ISCTR, and assigned scores for their ~~version~~variations of ~~14 of~~ these features. The assessed features include the nature of the content; the number and nature of fields to conduct a search; data download formats; the nature of the audit trail; the health condition category; the documentation available on a registry website; and so on. Overall, the

registries received between 27% and 80% of the maximum score of 94. The results from our analysis were used to define a set of features of an interim ideal registry. ~~These include the number and nature of fields to conduct a search; data download formats; the nature of the audit trail; and the health condition category. The main limitations of the study are that (i) it does not assess all of the recommendations of the ISCTR, and (ii) although applied systematically, the absolute values of the scores are arbitrary.~~

Conclusions

To the best of our knowledge, this is the first study to quantify the widely divergent quality of the primary registries' compliance with the ISCTR. Even with this limited assessment, it is clear that some of the registries have much work to do, although a mere dozen improvements would significantly improve them. ~~Our study would be helpful to researchers who may wish to extend this audit and evaluate the completeness of the records or the quality of their data, two other major issues, in all 18 registries.~~

Introduction

The first two calls for clinical trial registries were made in the 1970s [1]. One aimed to enhance the enro~~l~~lment of patients in ongoing trials, and the other to reduce the possibility of bias in the subsequent reporting of trial results, caused by the selective publication of those with positive outcomes. Since the year 2000, trial registries have proliferated. Nevertheless, it has been a long and sustained battle by many stakeholders – activists, journals, researchers, funders, governments and the World Health Organization (WHO) – to ensure that large numbers of trials are registered [2–4]. Although the initial two aims for setting up registries continue to be among the most important uses of such databases, researchers have utilized the data in at least a dozen other ways, such as (i) analyzing the conditions, the medical interventions, the sponsors and so on of Expanded Access Studies registered in the United

States (US) [5]; (ii) identifying the fraction of trials that have run in the country, that had industry involvement [6]; (iii) conducting a geo-temporal analysis of the trials of novel stem cell therapies [7]; (iv) obtaining information about a trial that was not reported in the subsequent publication [8]; and (v) identifying trials being run in contravention to the law [9].. Given these numerous and diverse purposes, not initially envisaged, it is even more important that all trials are registered and reported in a timely fashion, and that all the data in each record is complete, reliable and readily accessible. In view of this, the quality of data in the databases has long been the subject of analysis and comment. T[12,19–25]. hese include (a) analyses of the quality of registration and missing information in trial records [10–13], (b) studies on the discrepancies in trial status for trials that are registered in more than one registry [14], and (c) reports on the phenomenon of hidden duplicates [15,16]. Other studies have looked into the challenges faced, and advances made by individual registries [17,18]. The WHO's International Clinical Trials Registry Platform (ICTRP) initiative was designed Set up to facilitate access to clinical trial information around the world(19), the WHO's International Clinical Trials Registry Platform (ICTRP) initiative recognizes 17 registries as Primary Registries (PRs). Although ClinicalTrials.gov (CTG), of the United States (US), is not one of them, it is the oldest, and by far the largest, public registry, and is considered a data provider to ICTRP. The platform enables a search for trials in all 18 registries [26]. In this study, we have analyzed all of them, and refer to them, collectively, as Primary Registries(+), or PR+.

WHO's International Standards for Clinical Trial Registries (ISCTR) (20) lists the minimum, and sometimes ideal, standards that PRs should adopt to ensure a basic quality of data and accessibility. These standards are in nine sections.

Certain high profile scandals [19,20] resulted in numerous calls to increase transparency in clinical trials and to improve the public's trust in the trials enterprise. Following the Ministerial Summit on Health Research that took place in Mexico City in November 2004,

the WHO launched the International Clinical Trials Registry Platform (ICTRP) initiative in 2006 [21].

The ICTRP enables a single point of access to information regarding trials within its registry network [22], which hosts trial records from around the world. The network consists of (i) Primary Registries (PRs), (ii) Data providers, and (iii) Partner registries [23]. There are currently 17 PRs. The Data providers include the PRs and ClinicalTrials.gov (CTG), of the United States (US). All data providers need to fulfil the same criteria in terms of data collection and management. The two partner registries (i) are not required to fulfil the criteria that PRs need to; (ii) need to be affiliated with one of the PRs; and (iii) cannot directly feed data into the ICTRP [24]. Therefore, we have not included the these two registries in our study. We have analyzed the 17 PRs, and CTG, and refer to them, collectively, as Primary Registries Plus, or PR+.

The WHO also developed the International Standards for Clinical Trial Registries (ISCTR) [25], which lists the minimum, and sometimes ideal, standards that PRs should adopt to ensure a basic quality of data and accessibility. These standards are in nine sections — Content; Quality and Validity; Accessibility; Unambiguous Identification; Technical Capacity; Administration and Governance; the 24-field Trial Registration Data Set (TRDS); Partner Registries; and Data Interchange Standards. Although, ICTRP stipulates that non-compliance with these standards can result in revoking of Primary Registry status, we are unaware of any case where this has happened.

It is known that users trust public registries more than those created by companies or patient groups [26]. Also, public registries are often the primary sources on which other databases are built [26]. It follows that the information in each one should be comprehensive, high quality and available in a user-friendly fashion. Accordingly, there have been calls for (i) a

comparison of such registries, to help develop suitable standards [25], and (ii) ways to improve the accessibility and content of the PR+ [27]. However, several years ago it was shown that there had been non-compliance with the WHO minimal dataset [28], and non-optimal website functionality and user experience[10,11,29]. Since across-the-board improvements have not taken place, this issue needs to be reiterated. However instead of undertaking a purely qualitative assessment, we drew inspiration from other researchers' scorecards. These scorecards have either been developed [30–33] or proposed [34,35] to track whether trialists register their studies and report the results accurately, comprehensively and on time. Accordingly, we developed one to assess the PR+.

~~We wished to undertake a comparative assessment of the PR+, to assess their implementation of ISCTR. In order to do so, ~~W~~w~~e have developed the Registries' Comparative Scorecard (the Scorecard) which rates the PR+ on certain features that map to ~~various ~~different sections of the ISCTR (S1 Table). We ~~end by defining ~~then define an interim 'ideal registry' based on the best ~~version~~variations of each feature used by the PR+. Until such time as all the registries adopt all the standards recommended by ISCTR, the adoption of the recommended ~~version~~variations of each feature would be very helpful for users.

Methods

Data collection

We accessed the websites of the 18 PR+ between July 2019 and April 2020, inclusive. The registries were evaluated for 14~~17~~ features that map to one or more of the nine sections of ISCTR mentioned above.~~, that is (i) Content; (ii) Quality and Validity; (iii) Accessibility; (iv) Unambiguous Identification; (v) Technical Capacity; (vi) Administration and Governance; (vii) The WHO's mandated 24-field Trial Registration Data Set (TRDS); (viii) Partner~~

~~Registries; and (ix) Data Interchange Standards.~~ The list of features was compiled by the authors based on literature regarding the necessity of higher quality trial registrations [36,37], focusing on the standards listed in ISCTR [25].

All information was obtained from one or more of the following resources within each PR+ website: (i) the general pages of the site; (ii) a randomly chosen, sample interventional trial that was registered after 1 January 2019; (iii) supporting documents, if available; and (iv) where necessary and possible, via a login to do a mock registration. All analyses were performed by one author (NV) and verified by the other (GS), with differences resolved by discussion.~~. Further methodological details and reference URLs are available in the Supplementary files, which are referenced in the Results as relevant.~~

The sections below provide further methodological details on the data collection for, and analysis of, each of the features analysed, which have been classified based on the ISCTR section they map to. Reference URLs are available in the Supplementary files, which are referenced in the Results as relevant.

I. Accessibility features

We first examined the accessibility of information in the PR+. For this, we assessed several features, as follows:

(i) The ease of obtaining the total number of trials hosted by the registry:

The method of obtaining the total number of trials hosted by each PR+ was determined. Specifically, we documented (a) whether the number was displayed on the home page, (b) if it was available after a search, or (c) whether it had to be calculated based on the number of pages of results. If there was discrepant information at different places on the site, this fact was captured.

(ii) The existence of a Basic search function:

We examined the presence of the search function using a basic search field.

(iii) and (iv) The number of TRDS fields, and extra fields, that can be used to conduct a search:

We documented the presence and number of (iii) TRDS fields; and (iv) Extra fields, beyond these 24 TRDS fields, that can be used to conduct a search.

(v) The data download options:

For each PR+, we documented the file formats that are available for data download. We also captured information on whether the data on (a) one, (b) a limited number, (c) multiple, or (d) all search results can be downloaded at a time.

II. Content or compliance with TRDS features

(i) TRDS fields and Extra fields:

Each registry provides information about a trial in two different 'views'. While conducting a search, the user first obtains a list of trials which contains the titles, and may also contain other information. This is called the Brief view. Each trial record is available as a Brief view, and a Detailed view. The fields available in these views in each registry were documented. This information was then mapped to the 24 fields of the WHO TRDS. All additional fields were categorized as 'Extra fields'.

Among the Extra fields, we looked into the following features in further detail.

(ii) Whether the Principal Investigator (PI) name is compulsory:

Even though the ISCTR states that the PI is the ‘Contact for Scientific Queries’, unless the PI delegates this task to somebody else, the PR+ have not ~~adopted a~~ uniformly adopted this ~~definitio~~definition, and it is not always clear if the ‘Contact for Scientific Queries’ reflects th~~n~~ ~~for the~~ the PI. Therefore, we have separately looked into whether the PI name specifically, is compulsory.

(iii) The audit trail of each record:

We wished to know whether, where relevant, a given trial (a) has an audit trail, and if so, (b) whether the changes are clearly highlighted; and (c) whether two versions of the record can be readily compared. In some cases where the sample trial, used for most analyses, did not have a history of changes, we used another sample trial, whose URL is provided in S5 Table.

(iv) The flagging of retrospectively registered trials:

We documented whether each PR+ specifically mentions the registration status of the trial (prospective vs retrospective), or flags retrospectively registered trials.

(v) The reason for the termination of a trial, if applicable:

In this case, we first determined whether the PR+ have a category of terminated trials. For those that do, we captured whether or not a reason for trial termination is provided.

III. Quality and Validity, Technical Capacity, and Data Interchange Standards features

(i) Use of a controlled vocabulary for the health condition category:

We evaluated whether (a) there is a drop down menu for choosing a term from a controlled vocabulary, (b) the registry recommends a widely used controlled vocabulary, or (c) the trialist has to use a free text box.

(ii) The availability of documentation for the processes of the registry, or information on the site:

We evaluated the presence of three types of documents, that is (a) a glossary or the definition of each field of the record, (b) a list of frequently asked questions (FAQs) and (c) one or more user guides.

(iii) Security features of the registry website:

The websites were checked for the presence of a basic security feature, an SSL certificate, as reflected in an 'https' in the website URL, instead of an 'http'.

Box 1. Terminology used in the study

Here, we list a few terms that have been used throughout the study, along with a description of what these refer to:

(a) the word 'section' only refers to one or more of the nine sections of the ISCTR;

(b) the word 'feature' only refers to one or more of the 14 features of each registry that are the focus of this study; and

(c) since the different registries may have different variants of each 'feature', we use the word 'variation' in this context.

The ~~Registries' Comparative~~ Scorecard ~~(the Scorecard)~~

Overall, 14 features of the PR+ were assessed. Each registry has a particular variant of a given feature, which~~. This variant~~ may be more useful or less so. A scoring rationale was devised for every feature analysed, based on which each registry received a score for its variation of a given feature. The rationale is described in detail in Table 1, and further details are provided in the Results and Discussion section.

The following general rules were applied for the scoring system. These are illustrated by particular features in Table 1.

(i) I~~i~~f the feature is absent, the registry gets a score of 0. This is illustrated ~~by~~in features 1.2 and 1.5~~ in~~.

~~-~~(ii) F~~f~~or features with multiple ~~variants~~variations, the score ranges from 1 to 5 based on pre-set criteria, as defined in Table 1. This is illustrated ~~by~~in features 1.1 and 2.1~~ in Table 1~~.

(iii) F~~f~~or certain features, which involve counts of fields present, the score increases by ~~one~~ 1 point per field. This is illustrated ~~by~~in features 1.3 and 2.2~~ in Table 1~~.

(iv) In case a registry has multiple possible scores for a particular feature, the highest one is awarded. This is illustrated ~~by~~in feature~~s~~ 1.5 ~~in Table 1~~.

Table 1. Rationale for score given to each registry for features used to create the Scorecard.
The relevant Supplementary files with further details are also referenced.

	**Feature analyzed**	**Rating scale and rationale**	**Relevant Supplementary file**
**1**	**Accessibility**		
1.1	Total number of trials in the registry	Number displayed on home page: 5<br>Number available after a search: 3<br>Number needs to be calculated: 2<br>Discrepant information at different places on the site: 1	S2 Table
1.2	Existence of Basic search function	Presence of a basic search function: 5<br>Absence of a basic search function: 0	S2 Table
1.3	Advanced search function – TRDS fields	Each TRDS field: 1	S2 Table
1.4	Advanced search function – Extra fields	Each extra field: 1, but with a cap of 5 overall, because of the idiosyncratic nature of some of the search possibilities.	S2 Table
1.5	Data download options	Excel/csv/tsv: 5<br>HTML/XML: 2<br>Word/txt/pdf: 1<br>No download options: 0<br>Since all the registries except NTR permit HTML downloads (even if it is not explicitly stated), no registry gets a rating of '1'.	S2 Table
2	**Content or TRDS sections**		
2.1	Brief view: TRDS fields	10 or more fields, which are customizable, and wrapping of text: 5<br>10 or more fields, which are customizable, but without wrapping of text: 4<br>A fixed number of fields, that are more than 3: 3<br>Up to 3 fields: 1	S3 Table
2.2	Brief view: Extra fields	Each field: 1 point	S4 Table
2.3	Detailed view: TRDS fields	The number of fields over 20	S3 Table
2.4	Detailed view: Extra fields	Each field: 1 point<br>In this case, the maximum score is dictated by the registry with the maximum number of fields.	S4 Table
2.5	Whether PI name is compulsory	PI name is compulsory: 5<br>It is not clear whether the scientific contact is the PI (regardless of whether or not this information is compulsory): 2<br>There is a field for the PI name, but it is not clear whether the information is compulsory: 2<br>The PI name is voluntary: 0	S5 Table
2.6	Audit trail	Each of the following aspects receives 1 point: (i) the existence of an audit trail; (ii) the changes made are clearly highlighted; and (iii) it is possible to compare any two versions of the record.	S5 Table
**3**	**Other Sections**		
3.1	Health condition	A drop-down menu for choosing a term from a controlled vocabulary: 5<br>A widely used controlled vocabulary is recommended: 3<br>Free text field: 1	S5 Table
3.2	SSL certificate	Website secured with SSL: 3<br>Website not secured with SSL: 0	S5 Table
3.3	Documentation	Provides (a) a glossary or the definition of each field of the record; (b) List of FAQs; (c) One or more user guides: 1 point each. No points are awarded for the quality of these documents.	S5 Table

Results and Discussion

We first documented basic information about each of the registries., The full name of each registry, its acronym, the country where it is based, and the year it was established are provided in Fig.1 and Table 2. Except CTG, the acronyms used for each registry were are the official acronyms. All but one of the PR+ were established between 2000 and 2010, inclusive. LBCTR was established in 2019. Eight registries (ANZCTR, ChiCTR, CTG, DRKS, IRCT, ISRCTN, JPRN, and SLCTR) allow trial registrations from all countries, and the rest usually from the country where the registry is based, or from specific countries or regions. For example, PACTR caters to clinical trials conducted in Africa. -On 18 April 2020, the registries cumulatively held 5,72,901 records, with CTG accounting for 336,444 (59-%)of them.

Trials may be registered either prospectively or retrospectively, that is before the enrolment of the first participant or after. Six of the PR+ (CTRI, IRCT, LBCTR, REPEC, SLCTR and TCTR) only allow prospective registration, whereas the remaining accept retrospective as well. Five of the PR+ (EU-CTR, IRCT, PACTR, REPEC and SLCTR) accept only interventional clinical trials, while the remaining may accept others such as observational studies, post marketing surveys or expanded access programs. All the registries use English, and 11 of them display some or all information in another language as well.

Figure legend

Fig. 1:

A. The timeline of establishment of the PR+. Key events related to trial registration are also noted.

B. Number of records per registry as on 18 April 2020. The pie chart shows the distribution of the number of records in each registry. The actual number, and as a percentage of the total, are also provided.

Table 2~~1~~. An overview of each registry, listing its acronym, full name, country where it is based,~~ year established, number of trial records held,~~ countries from where registration is accepted, type of registration allowed, type of study hosted, and language used.

Registry acronym	Registry full name	Country where registry is based	Countries from where registration is accepted	Type of registration allowed	Type of study	Additional language[1~~2~~]
ANZCTR	Australian New Zealand Clinical Trials Registry	Australia	All countries. However, trials in Australia and New Zealand are prioritized	Prospective, Retrospective[2~~3~~]	Interventional, Observational	–
ChiCTR	Chinese Clinical Trial Register	China	All countries	Prospective, Retrospective	Interventional, Observational, Others	Chinese
CRIS	Clinical Research Information Service	Republic of Korea	Republic of Korea	Prospective, Retrospective	Interventional, Observational	Korean
CTG[3~~4~~]	ClinicalTrials.gov	USA	All countries	Prospective[~~5~~] Retrospective	Interventional, Observational, Expanded Access	–
CTRI	Clinical Trials Registry - India	India	Other countries in the region which do not have a Primary Registry of their own[4~~6~~]	Prospective	Interventional, Observational, PMS[5~~7~~], BA/BE[5~~7~~]	–
DRKS	German Clinical Trials Register	Germany	All countries	Prospective, Retrospective	Interventional, Observational, Epidemiological, Others	German
EU-CTR	EU Clinical Trials Register (EU-CTR)	The Netherlands	All interventional trials that have at least one centre in the EU and EEA. Certain trials conducted entirely outside these regions.	Prospective. Retrospective if permitted by National Competent Authority of the Member State	Interventional	Older trials may have content in the host country's language
IRCT	Iranian Registry of Clinical Trials	Iran	All countries	Prospective	Interventional	Persian
ISRCTN	International Standard Registered Clinical/soCial sTudy Number	UK	All countries	Prospective, Retrospective[2~~3~~]	Interventional, Observational	–
JPRN	Japan Primary Registries Network[6~~9~~]	Japan	All countries	Prospective, Retrospective	Interventional, Observational	Japanese
LBCTR	Lebanon Clinical Trials Registry	Lebanon	Lebanon	Prospective	Interventional, Observational	Brief summary of the study is also available in Arabic

NTR	Netherlands Trial Register	The Netherlands	Trials conducted in Netherlands or involving Dutch researchers.	Prospective, Ongoing studies	Interventional, Observational	Some information may be available in Dutch
PACTR	Pan African Clinical Trials Registry	South Africa	All countries in Africa	Prospective, Retrospective	Interventional	–
ReBEC	Brazilian Registry of Clinical Trials	Brazil	Brazil[4][6]	Prospective Retrospective	Interventional, Observational	Portugese and Spanish, for some records, and in a limited way
REPEC	Peruvian Clinical Trial Registry	Peru	Peru	Prospective	Interventional	Spanish
RPCEC	Cuban Public Registry of Clinical Trials	Cuba	Cuba[7,10]	Prospective, Retrospective	Interventional Observational	Spanish
SLCTR	Sri Lanka Clinical Trials Registry	Sri Lanka	All countries	Prospective	Interventional	–
TCTR	Thai Clinical Trials Registry	Thailand	Thailand	Prospective	Interventional, Observational	–

1.
All registries are required to be in English. However, some provide content in additional language(s).
2. Retrospective registration is allowed but prospective registration is preferred and encouraged.
3. Except CTG, all the acronyms listed are the official acronyms.
4. For two registries (CTRI, REBEC) the information on the ICTRP portal and on their own websites is discrepant. Upon inspection, the latter sources appear to be correct, and we have described the registries accordingly.
5. PMS: post-marketing surveillance; BA/BE: Bioavailability/Bioequivalence.

6. Common forum for trials from three Japanese registries, that is (UMIN Clinical Trials Registry (UMIN-CTR), Japan Pharmaceutical Information Center Clinical Trials Information (JAPIC-CTI), and Japan Medical Association - Center for Clinical Trials (JMACCT)).
7. Trials are accepted from Cuban sponsors, conducting trials in Cuba or abroad, with Cuban or foreign products.

Notably, some registries (ChiCTR, EU-CTR, ISRCTN, PACTR, REPEC) were built on earlier versions.

We then analysed 14 features of the PR+, which have been grouped according to the sections of ISCTR that they map to (S1 Table). In Table 3, we list the score obtained by each PR+ per feature, and overall. We also provide the maximum score possible per feature. Further details are provided below, or are available in relevant Supplementary files, which are referenced in Table 1. As mentioned, we analyzed 17 features of the PR+. However, as explained below, we evaluated only 14 of them to develop the Scorecard, details of which are provided in Table 2a. Here, we list the various features analyzed, and the maximum score possible for the feature, We also provide the score obtained by each PR+ per feature, and overall. To be noted, ISCTR recommends 24 Trial Registration Data Set (TRDS) fields. Any other field was referred to as an Extra field. In Table 2b , we describe the rationale for the scores, and the

~~weightage given to each variant of each feature. Further details are provided below, or are available in relevant Supplementary files, which are referenced in Table 2b. We have grouped the features according to the sections of ISCTR that they mapped to.~~

Table 3~~2a~~. The Scorecard. ~~[We would like Table 2b to be part of 2a, on the right, but the uploading system didn't permit it.]~~

The list of features used to create the Scorecard; the maximum score per feature; the score obtained by each registry per feature, and overall per section; the total score per registry; and the rank of each registry.

		Max score	ANZCTR	ChiCTR	CRIS	CTG	CTRI	DRKS	EU-CTR	IRCT	ISRCTN	JPRN	LBCTR	NTR	PACTR	ReBEC	REPEC	RPCEC	SLCTR	TCTR
**1**	**Accessibility section**																			
1.1	Total number of trials in the registry	**5**	3	3	5	5	3	3	5	5	3	3	1	3	3	5	3	2	2	3
1.2	Existence of Basic search function	**5**	5	0	5	5	5	5	5	5	5	5	0	5	5	5	5	5	0	5
1.3	Advanced search function – TRDS fields	**24**	11	17	14	15	10	8	7	17	13	0	9	0	12	4	1	5	2	7
1.4	Advanced search function – Extra fields	**5**	1	5	5	5	4	5	3	5	5	0	0	0	2	1	2	0	0	0
1.5	Data download options	**5**	5	2	2	5	2	5	2	2	5	5	2	0	2	2	2	2	2	2
	**SUB-TOTAL**	**44**	**25**	**27**	**31**	**35**	**24**	**26**	**22**	**34**	**31**	**13**	**12**	**8**	**24**	**17**	**13**	**14**	**6**	**17**
**2**	**Content or TRDS sections**																			
2.1	Brief view: TRDS fields	**5**	3	3	4	5	3	3	3	3	1	3	3	1	3	3	3	1	1	5
2.2	Brief view: Extra fields	**5**	3	1	2	2	0	3	1	3	1	1	1	0	3	0	0	1	5	1
2.3	Detailed view: TRDS fields	**4**	4	4	3	4	3	2	2	2	4	4	4	3	4	0	3	1	4	3
2.4	Detailed view: Extra fields	**15**	10	5	10	15	8	6	9	7	10	0	9	5	5	4	10	6	5	9
2.5	Whether PI name is compulsory	**5**	5	5	5	0	0	5	5	2	2	2	2	2	5	0	2	5	2	2
2.6	Audit trail	**3**	1	1	3	3	1	3	0	3	2	0	1	0	3	0	0	3	2	0
	**SUB-TOTAL**	**37**	**26**	**19**	**27**	**29**	**15**	**22**	**20**	**20**	**20**	**10**	**20**	**11**	**23**	**7**	**18**	**17**	**19**	**20**
**3**	**Other sections**																			
3.1	Health condition	**5**	5	3	5	3	5	5	5	3	1	5	5	1	5	5	3	1	1	3
3.2	SSL certificate	**5**	5	0	5	5	0	5	5	5	0	5	0	5	5	0	5	0	5	5

3.3	Documentation	**3**	3	2	2	3	3	3	3	1	3	0	3	0	2	3	2	2	2	3
	**SUB-TOTAL**	**13**	**13**	**5**	**12**	**11**	**8**	**13**	**13**	**9**	**4**	**10**	**8**	**6**	**12**	**8**	**10**	**3**	**8**	**11**
	**TOTAL**	**94**	**64**	**51**	**70**	**75**	**47**	**61**	**55**	**63**	**55**	**33**	**40**	**25**	**59**	**32**	**41**	**34**	**33**	**48**
	**% of TOTAL**		**68**	**54**	**74**	**80**	**50**	**65**	**59**	**67**	**59**	**35**	**43**	**27**	**63**	**34**	**44**	**36**	**35**	**51**
	**Rank of each registry**		**3**	**9**	**2**	**1**	**11**	**5**	**7**	**4**	**7**	**15**	**13**	**18**	**6**	**17**	**12**	**14**	**15**	**10**

Table 2b. Rationale for weightage given to each registry for features used to create the Scorecard.

The relevant Supplementary files with further details are also referenced.

	Feature analyzed	Rating scale and rationale	Relevant Supplementary file
**1**	**Accessibility**		
1.1	Total number of trials in the registry	Number displayed on home page: 5<br>Number available after a search: 3<br>Number needs to be calculated: 2<br>Discrepant information at different places on the site: 1	S2 Table
1.2	Existence of Basic search function	Presence of a basic search function: 5<br>Absence of a basic search function: 0	S2 Table
1.3	Advanced search function – TRDS fields	Each TRDS field: 1	S2 Table
1.4	Advanced search function – Extra fields	Each extra field: 1, but with a cap of 5 overall, because of the idiosyncratic nature of some of the search possibilities.	S2 Table
1.5	Data download options	Excel/csv/tsv: 5<br>HTML/XML: 2<br>Word/txt/pdf: 1<br>No download options: 0<br>Since all the registries except NTR permit HTML downloads (even if it is not explicitly stated), no registry gets a rating of '1'.	S2 Table
**2**	**Content or TRDS sections**		
2.1	Brief view: TRDS fields	10 or more fields, which are customizable, and wrapping of text: 5<br>10 or more fields, which are customizable, but without wrapping of text: 4<br>A fixed number of fields, that are more than 3: 3<br>Upto 3 fields: 1	S3 Table
2.2	Brief view: Extra fields	Each field: 1 point	S4 Table
2.3	Detailed view: TRDS fields	The number of fields over 20	S3 Table
2.4	Detailed view: Extra fields	Each field: 1 point<br>In this case, the maximum score is dictated by the registry with the maximum number of fields.	S4 Table
2.5	Whether PI name is compulsory	PI name is compulsory: 5<br>It is not clear whether the scientific contact is the PI (regardless of whether or not this information is compulsory): 2<br>There is a field for the PI name, but it is not clear whether the information is compulsory: 2<br>The PI name is voluntary: 0	S5 Table
2.6	Audit trail	Each of the following aspects receives 1 point: (i) the existence of an audit trail; (ii) the changes made are clearly highlighted; and (iii) it is possible to compare any two versions of the record.	S5 Table
**3**	**Other Sections**		
3.1	Health condition	A drop-down menu for choosing a term from a controlled vocabulary: 5<br>A widely used controlled vocabulary is recommended: 3<br>Free text field: 1	S5 Table
3.2	SSL certificate	Website secured with SSL: 3<br>Website not secured with SSL: 0	S5 Table
3.3	Documentation	Provides (a) a glossary or the definition of each field of the record; (b) List of FAQs; (c) One or more user guides: 1 point each. No points are awarded for the quality of these documents.	S5 Table

1. Accessibility:

One of the principal reasons for the existence of clinical trial registries is to provide the public with information, and to thereby increase trust in the trial enterprise [28]. Therefore, we first examined the accessibility of information in the PR+. For this, we assessed several features, as described below:

(ia) Ease of obtaining the total number of trials

As a first step, it is important to know how many records the database holds. This number should be readily available, and we have therefore analysed the ease of accessing it. The five registries (CRIS, CTG, EU-CTR, IRCT and ReBEC) that list it on the homepage were given the highest score of 5. Ten registries display this number after a search for all trials, and received a score of 3. Two registries (RPCEC, SLCTR), for which the number of records is available only by a manual calculation, received 2. LBCTR provides discrepant information at different places on the site, and thus received the lowest score of 1. The median score obtained was 3. It is a trivial task to put the figure for the total number of trials on the home page, and we encourage all registries to do so

Coming to the search functions, for nearly allFor a significant fraction of users, the search functionsse are crucially important to access the information in a registry. ISCTR recommends that at the minimum, there must be a basic text search, as well asand it must be possible to searches within the interventions and conditions fields. Several PR+ go much further than this, and therefore we have conducted a detailed assessment of their search capabilities.

(ii) Basic Search function

We determined the presence of a basic search function and have awarded a score of 5 to the 15 registries that provide it. Only three (ChiCTR, LBCTR and SLCTR) do not have this feature, and received 0. The median score was 5.

Most PR+– have a basic search function that enables search by keywords. This is a crucial aspect of the functionality of the trial registry website, and significantly increases the ease of searching for information and improves user experience.

(iiic) Advanced search function - TRDS fields

We then examined how many of the 24 TRDS fields could be used in the Advanced search function. Out of a possible score of 24, where the registries received 1 point per field, the maximum score of 17 was attained by ChiCTR and IRCT. JPRN and NTR do not allow a search by any TRDS field and received 0. The remaining registries received scores between 1 and 15. The median score was 8.5.

(ivd) Advanced search function - Extra fields

A few registries list fields other than the TRDS fields as part of the search function. Six PR+ (ChiCTR, CRIS, CTG, DRKS, IRCT and ISRCTN) have five or more Extra fields, and therefore received a score of 5. Six registries (ANZCTR, CTRI, EU-CTR, PACTR, ReBEC and REPEC) received scores ranging from 1–4. Six registries do not allow a search using any Extra fields, and received 0. The median score was 2.

Overall, the PR+ provided more fields in the Advanced search function than the minimum recommended by the ISCTR. This becomes especially relevant for researchers conducting

systematic reviews, work which requires extensive searches to gather information on clinical trials in specific areas.

(v) Data download options

Having conducted a search, users may wish to download many fields of data, for many records. We therefore gave the highest score of 5 to the five registries (ANZCTR, CTG, DRKS, ISRCTN and JPRN) that allow data downloads in a csv, excel or tsv format. 12 registries provide HTML and XML options, and received a score of 2. Only NTR lacks any options for data download, and received 0. The median score was 2.

All the available data download options are adequate for the inspection of a few records, but it is essential that the PR+ provide bulk data download options such as csv, especially as an increasing number of users are shifting towards automated systems of analysis.

2. Content and TRDS sections

Next, we examined multiple features that map to the Content or TRDS sections, which overlap since the TRDS fields are a form of content. Below, we describe our scoring of the Brief and Detailed views of the PR+.

(i) Brief View: TRDS fields

Since the Brief view is primarily designed to provide an overview of the trial, it can be very helpful for a user if the number of fields in the Brief view can be customized. Therefore, we have given higher scores to registries that provide this option. Two registries (CTG and TCTR), display more than 10 TRDS fields, and allow customization and text wrapping. They received the maximum score of 5. CRIS displays more than 10 TRDS fields, that are customizable but without text wrap, and received 4. Eleven registries display more than three

fields, which are fixed, and got a score of 3. The four registries (ISRCTN, NTR, RPCEC and SLCTR) that display three fields or less received 1. The median score was 3.

A customizable brief view of search results is extremely useful in a trial registry, where different types of users such as patients, healthcare professionals or sponsors, may be interested in different fields.

(ii) Detailed View: TRDS fields

The Detailed view tends to have all 24 TRDS fields. However, we found that all the PR+ do not yet list the four fields that have been included in the latest version of TRDS [29]. Eight registries (ANZCTR, ChiCTR, CTG, ISRCTN, JPRN, LBCTR, PACTR and SLCTR) do so, and received the highest score of 4. Most of the remaining PR+ display between one and three of the new fields and were scored accordingly. Only one registry, ReBEC, has not been updated to display any of the new fields, and received a score of 0. The median score was 3. We hope that over time more registries will be in full compliance with the ISCTR-mandated fields.

(iii) Extra fields

Registries list Extra fields in both the Brief and Detailed views. In the Brief View, only SLCTR received the maximum score of 5. Four registries (CTRI, NTR, ReBEC and REPEC) have no Extra fields and received a score of 0. The remaining have between one and three of such fields and were scored accordingly. The median score was 1.

In the Detailed View, most registries have between five and 10 Extra fields. However CTG has 15, and JPRN has none. The median score was 7.5.

Some of the Extra fields, such as the date of last update, and whether registration was prospective or retrospective, are recommended by ISCTR. Interestingly, one-third or more of

the registries list several fields that the ISCTR does not specifically recommend. This seems to reflect a certain level of agreement among the managers of registries that particular fields are important. There may be a range of reasons for including these ~~Extra~~ fields. For instance, India had been criticized for the lack of appropriate oversight to ensure the ethical conduct of trials, and therefore CTRI asked trialists for details of the ethics committee even before ISCTR required this information~~did~~ [38,39]. Also, there have been demands from the Cochrane collaboration, and many other individuals and groups, to include several additional items in the ISCTR list, which WHO has not agreed to. It is alleged that the recommended list is closer to what industry demanded [40]. As such, although ISCTR may not list every field that many people believed to be essential, managers of particular registries may have chosen to list some of them.

We explored some of these Extra fields in greater detail below.

(iv) Whether PI name is compulsory

For the sake of accountability it is important that the field ‘PI name’ is compulsory [25,41]. Although we have assessed Contact for Scientific Queries as a TRDS field, we have not assumed that this person is the PI, and therefore have separately looked into whether the PI name is compulsory. In s~~S~~even registries (ANZCTR, ChiCTR, CRIS, DRKS, EU-CTR, PACTR and RPCEC) it is so, and they received the highest score of 5. Several have either not made it clear whether the scientific contact is the PI, or have a separate field for the PI name but have not stated whether it is compulsory. They each received a score of 2. Three registries (CTG, CTRI and ReBEC) have marked this field as voluntary, and received 0. The median score was 2.

WHO documents [25,41] have contradictory information on the issue of PI name and Contact for Scientific Queries. They require that the PI’s name, title and email ID be provided, but

state that this should be a functional name, not a personal one. ISCTR states that the PI is the Contact for Scientific Queries, unless the PI delegates this task to somebody else. If the PI name is compulsory – and preferably recorded in a fixed format [42] – then this information will enable researchers to quantify the number of unique PIs in a country, ask whether a PI has been taking on too many trials, and perform other analyses. Therefore we commend the registries that have made this field compulsory.

(v) Audit trail

ISCTR requires that the audit trail of each record should be publicly available and so we have examined the presence and usefulness of this feature. Six registries (CRIS, CTG, DRKS, IRCT, PACTR and RPCEC) have the option of comparing two versions of the trial record and received the maximum score of 3; two (ISRCTN and SLCTR) have highlighted the changes made to a trial record, and got 2; four (ANZCTR, ChiCTR, CTRI and LBCTR) have a basic form of an audit trail and got 1; and six of the PR+ do not provide any audit trail and got 0. The median score was 1. It is clear that most registries do not have an ideal audit trail.

The information pertaining to the following two features is present in the Extra fields, either as a separate field in the Detailed view, or marked with a flag in the Brief View. Hence we have not scored these features separately.

(vif) Flagging retrospective or prospective registration status of a trial

Prospective registration is crucial to prevent unrecorded ‘outcome switching’, which creates a bias in the medical evidence base [28]. Nevertheless, it has been argued that (i) it is a duty to trial participants to register each trial, and subsequently publish the results, and (ii) not registering a trial could lead to its loss from the documented universe of trials- [43].

As such, retrospective registration is better than non-registration, and therefore many PR+ permit it. We have documented this in Table 1.

Users may have more confidence in the results of a prospectively than a retrospectively registered trial. Further, flagging retrospectively registered ones may shame the registrants into registering prospectively in future [14]. Accordingly, we have analysed whether PR+ highlight the registration status of trials and flag retrospectively registered ones. Over half of the PR+ do so.

(viig) The reason for the termination of a trial, if applicable

It is important to know why a study was terminated as it provides economic, ethical and scientific insights that can help improve ongoing or upcoming clinical trials [44]. Our analysis showed that only eight registries provide this information at all, and only three provide drop-down menus of reasons for termination (Table S5). Researchers who have studied the leading causes of trial termination have suggested that the cause should be selected from a fixed set of options [45].

3. Other sections:

Finally, we examined three features that map to other sections of the ISCTR, as follows: (a) Health condition, (b) the presence of a Secure Sockets Layer (SSL) certificate and (c) Documentation.

(i) Health condition

First, the issue of classifying health conditions, which maps to Data Interchange Standards. We find that only half the PR+ provide drop-down menus for this field, and they received the highest score of 5. Five registries (ChiCTR, CTG, IRCT, REPEC and TCTR) recommend the use of standardized vocabulary, and received 3. Four registries (ISRCTN, NTR, RPCEC and

SLCTR) that do not provide such options, and have a free text field for health condition, received 1. The median score was 4.

Comparisons across registries are easier if each one uses a controlled vocabulary, and in particular one that maps to a widely-used metathesaurus [46] as recommended in ISCTR [25]. It is therefore preferable that the health condition be selected from a fixed set of options.

(ii~~b~~) The presence of an ~~Secure Sockets Layer~~ ~~(~~SSL~~)~~ certificate

Second, the security of the website. In the Technical Capacity section, ISCTR requires that each registry have adequate protection against the corruption or loss of data. We have assessed something basic, that is whether the website is secured with an SSL certificate, as is evident when a website URL contains ‘https’. We find that only 12 of the PR+ websites have this certification, and ~~have been given~~each received a score of 5. The remaining six registries (ChiCTR, CTRI, ISRCTN, LBCTR, ReBEC and RPCEC) have URLs with an ‘http’, and received 0. The median score was 5.

The SSL certificate is an important tool to safeguard data of the registry and that of its users, and Google currently marks all sites without it as insecure [47]. As such, it is also a sign of credibility for a user who may hesitate to access a site ~~without~~that lacks a security certificate.

(iii) Documentation

Third, the issue of documentation. Various documents help users to understand the processes of a registry, or the data it hosts. Only half the PR+ ~~(9 registries)~~ provide all the three types of documentation we have assessed, and received a score of 3. Six registries (ChiCTR, CRIS, PACTR, REPEC, RPCEC and SLCTR) have only two types of documents and received 2, and one registry (IRCT) displays only a user guide and received a score of 1. Two registries

(JPRN and NTR) do not provide any documentation, and have received 0. The median score obtained was 2.5.

Although the three documents that we have scored are not explicit requirements of the ISCTR, they assist users in registering their trial correctly. As such, this feature maps to the Quality and Validity section.

In general, w~~W~~e have barely touched upon Quality and Validity, since investigating the completeness or quality of the records in the PR+ would be a large exercise in itself. The sponsor needs to ensure a high quality trial record, but this may not happen, and ~~.~~ v~~V~~arious studies have highlighted ~~such~~ deficiencies in the records of different registries [10–13,48] ~~[eg. Mounika, Viergever papers, Ogino, Sangeeta paper, Yadav - Clinical trials registered in clinical trial registry of India: A survey]~~. It is also the duty of the managers of the registry to facilitate better quality trial registration, as has been recommended by ISCTR. Additionally, for several of the minimum standards recommended by ISCTR, either it is not possible for us to assess compliance, or the requirements do not immediately impact use of the registry data. Therefore we have also barely touched upon Unambiguous Identification (although Secondary identifying numbers, a field in TRDS, also maps to this section), Technical Capacity, and Data Interchange Standards. Further, we have not touched upon the sections (i) Administration and Governance, and (ii) Partner Registries.

Overall, as derived from an assessment of 14~~5~~ features described above, the maximum score that any registry obtained was 94 points (Table 3~~2~~). The PR+ received scores ranging from 27% (NTR) to 80% (CTG) of the maximum, with an average of 52%. Despite the limited nature of our audit, the lowest- and highest-scoring registries received scores that differ by over 50%. To the best of our knowledge, this widely divergent quality of the PR+ has not

been documented before. The maximum score that any registry could obtain is 94 points (Table 2). The PR+ received scores ranging from 27% (NTR) to 80% (CTG) of the maximum, with an average of 52%.

Discussion

As mentioned, the PR+ received scores ranging from 27% to 80% of the maximum score. This derives from an assessment of 14 features, many of which we discuss in Box 1, that largely map to (i) Accessibility, or (ii) Content or TRDS. We have barely touched upon Quality and Validity, since investigating the completeness or quality of the records in the PR+ would be a large exercise in itself. For several of the minimum standards recommended by ISCTR, either it is not possible for us to assess compliance, or the requirements do not immediately impact use of the registry data. Therefore we have also barely touched upon Unambiguous Identification (which Secondary identifying numbers, a field in TRDS, also maps to), Technical Capacity, and Data Interchange Standards. Further, we have not touched upon the sections (i) Administration and Governance, and (ii) Partner Registries. Despite the limited nature of our audit, the lowest- and highest-scoring registries receive scores that differ by over 50%. To the best of our knowledge, this widely divergent quality of the PR+ has not been documented before.

An ideal registry

We found that the registries show a high degree of variability for a given feature, ranging from a sophisticated ~~version~~ to a routine ~~variant~~ variation, or its complete absence. We have used the best ~~version~~variant~~s~~ of the features analyzed to define an interim ideal registry. In ~~this~~such a registry,

(i) the total number of trials is displayed on the home page;

(ii) a search is possible through (a) a basic search function, (b) each of the TRDS fields, and (c) a few extra fields;

(iii) the data download options include a csv, excel, or tsv format, and support automated bulk downloads;

(iv) the Brief view is customizable, with 10 or more fields, ~~which can be~~with text wrappinge~~d~~;

(v) the Detailed view includes all the TRDS fields;

(vi) there is clarity on whether or not the scientific contact is the PI;

(vii) the PI name is compulsory;

(viii) the reason for the termination of a trial is ~~is provided, after being~~ selected from a drop-down menu of possible reasons;

(ix) each trial has an audit trail that enables a comparison of any two versions;

(x) at the very least, the following documents are provided, in English: (a) a definition of each field of the record, (b) a list of FAQs, and (c) one or more user guides;

(xi) the website is secured with an SSL certificate; and

(xii) the health condition category ~~has~~ is chosen from a drop-down menu with ~~to enable trialists to choose a term from~~ a controlled vocabulary, preferably a widely used one.

Formatted: Heading 2

~~Although~~ The ISCTR recommends several other standard including higher data quality, more complete records and the reporting of results~~s~~.~~,~~ ~~and~~ Although it is hoped that all registries will implement all of ~~them~~ these standards in due course, in the interim, ~~all the~~ registries may wish to implement the list above if they have not already done so.

Registries have many users. The scorecard above analyses features that are of interest to the authors and, by extension, possibly to other researchers concerned with the health of the trial ecosystem overall. Other categories of users, such as medical professionals, patients, trial sponsors, policy makers, data scientists and so on, may wish to alter the assessed features, or the scoring, in order to rank the registries according to their priorities. For instance, a data scientist would be very appreciative of ANZCTR, which– specifically enables web crawling of its records [49]. Furthermore, the managers of other registries, either public or private, and either based on the data in the PR+ or not, may be interested in the results of this study.

The ongoing Covid-19 pandemic has forcefully brought home the need for high quality trial registries with information that is consistent, comprehensive and available in a user-friendly fashion. Billions of people need to be immediately protected from the virus, and large numbers of drugs and vaccines are in trials. There is world-wide interest in these trials, and information that is being tracked includes what is being trialled; where these trials are taking place; and the results of these trials. Each country needs to take public health decisions, which will evolve as the evidence from trials running in different parts of the world yield results. Public trial registries are one of the fastest ways of communicating these results.

Further, the publicly available, freely accessible information in such registries helps to build trust with the public [~~45,46~~26,44]. Covid-19 trials have been among the fastest recruiting ones in history ~~[45] [NV: These two links don't work for me.one shows error, the other requires subscription~~[50,51]~~]~~, and it is possible that the publicly available information in trial

registries has helped many of the potential trial participants decide to enrol.

It is not just that everyone is interested in the positive outcomes of trials. For example, an inspection of the CTRI records of hundreds of cCovid-19 trials being run in India has thrown up quality issues in almost all of them. Based on negative publicity, the government has taken action in some cases [52].

There is a long history of various stakeholders arguing for the need to improve registries and the quality of trial registration. Examples include academics and health activists [53–55], journals (ICMJE) [56], WHO ([41], registry managers [57], funders [58,59] and governments- [60]. Each of these efforts has led to some improvements in the number and quality of trial records hosted by registries. However none of them has led to a perfect set of records. It is likely that the only way this will be achieved is if all stakeholders continue to apply pressure on the registries. Studies such as this one help to highlight deficiencies, which adds to the other efforts aimed at improving registries. The authors would welcome other researchers' efforts to create and update a website that lists the scorecard, with periodic updates. Should such a website not be created by any other group, the authors intend to re-evaluate the registries' performance on the scorecard every few years.

In summary, to the best of our knowledge, this is the first study undertaking a comparative analysis of WHO--recognized registries to assess compliance to ISCTR. Our use of a scorecard, based on preset criteria, ensured an impartial quantification of the quality of the features analyzed across the PR+. .As such, even though our study analyzed a limited set of features, it clearly shows the substantial variation in compliance with the recommended minimal standards. Our study would be helpful to researchers who may wish to extend this audit and evaluate the completeness of the records or the quality of their data, two other major issues, in all 18 registries.

~~Registries have many users, such as patients and their families, clinicians, researchers, trial sponsors, policy makers and so on. It is the perspective of researchers such as the authors, concerned with the health of the trial ecosystem overall, that has guided the current analysis. Other users may have a different focus, and may wish to alter the assessed fields or the scores. Further, there are other registries, either public or private, and either based on the data in the PR+ or not, which may be interested in the results of this analysis.~~

This study has a few limitations, as follows: (i) It assesses only some of the many features in each registry. In particular, it does not evaluate any aspect of trial methodology or results, which are crucial ~~sections~~ portions of such registries. As such, otherwise outstanding registries may have fared less well than expected. (ii) We have not evaluated the completeness of any records or the quality of their data. (iii) Each registry has been evaluated with respect to the list of fields in a recently registered trial. Earlier records in the same registry may have different content if the required details have changed over time. (iv) We have primarily focused on information that is available in English and may have missed important content in other languages. (v) Although applied systematically, the absolute values of the scores are arbitrary~~.~~

Conclusions

Over the years, CTG has received most of the attention of those interested in the accessibility and integrity of the data in public trial registries. As noted above, 41% of the records are held in the other PR+, and ~~a searchlight needs to be turned on them~~need to be examined as well. We have identified the best ~~version~~variations of several features that have already been implemented by one or more of these registries, and which serve as pointers on how the others may improve. Running a registry is not merely a bureaucratic task, but is part of a

mission to safeguard patients’ lives, and the ethics and science of medicine. We hope that our analysis is of some assistance in this. ~~We also believe that our study would be helpful to researchers who may wish to extend this audit and evaluate the completeness of the records or the quality of their data, two other major issues, in all 18 registries.~~

References

1. Dickersin K, Rennie D. Registering clinical trials. JAMA. 2003 Jul 23;290(4):516-23. doi: 10.1001/jama.290.4.516. PMID: 12876095.

2. Krleza-Jerić K. Clinical trial registration: the differing views of industry, the WHO, and the Ottawa Group. PLoS Med. 2005 Nov;2(11):e378. doi: 10.1371/journal.pmed.0020378. Epub 2005 Oct 18. PMID: 16221000; PMCID: PMC1255765.

3. Lundh A, Krogsbøll LT, Gøtzsche PC. Access to data in industry-sponsored trials. Lancet. 2011 Dec 10;378(9808):1995-1996. doi: 10.1016/S0140-6736(11)61871-0. PMID: 22153200.

4. Lemmens T and Bouchard R, Mandatory Clinical Trial Registration: Rebuilding Public Trust in Medical Research (2007). GLOBAL FORUM UPDATE ON RESEARCH FOR HEALTH, Vol. 4: Equitable Access: Research Challenges for Health in Developing Countries, pp. 40-46, London: Pro-Book Publishing, 2007. Available at SSRN: https://ssrn.com/abstract=1083565.

5. Miller JE, Ross JS, Moch KI, Caplan AL. Characterizing expanded access and compassionate use programs for experimental drugs. BMC Res Notes. 2017 Jul 28;10(1):350. doi: 10.1186/s13104-017-2687-5. PMID: 28754150; PMCID: PMC5534121.

6. Askie LM, Hunter KE, Berber S, Langford A, Tan-Koay AG, Vu T et al. The clinical trials landscape in Australia 2006–2015. Sydney: Australian New Zealand Clinical Trials Registry 2017.

7. Li MD, Atkins H, Bubela T. The global landscape of stem cell clinical trials. Regen Med. 2014 Jan;9(1):27-39. doi: 10.2217/rme.13.80. Epub 2013 Nov 18. PMID: 24236476.

8. Tharyan P, George AT, Kirubakaran R, Barnabas JP. Reporting of methods was better in the Clinical Trials Registry-India than in Indian journal publications. J Clin Epidemiol. 2013 Jan;66(1):10-22. doi: 10.1016/j.jclinepi.2011.11.011. Epub 2012 Mar 27. PMID: 22459428.

9. Yasmeen A. Infant milk: Nestle’s clinical trial under scanner. The Hindu. 3 Aug 2019. Available: https://www.thehindu.com/news/national/infant-milk-nestles-clinical-trial-under-scanner/article28809296.ece. Accessed 25 Sep 2020.

10. Viergever RF, Ghersi D. The quality of registration of clinical trials. PLoS One. 2011 Feb 24;6(2):e14701. doi: 10.1371/journal.pone.0014701. PMID: 21383991; PMCID: PMC3044717.

11. Viergever RF, Karam G, Reis A, Ghersi D. The quality of registration of clinical trials: still a problem. PLoS One. 2014 Jan 10;9(1):e84727. doi: 10.1371/journal.pone.0084727. PMID: 24427293; PMCID: PMC3888400.

12. Chaturvedi N, Mehrotra B, Kumari S, Gupta S, Subramanya HS, Saberwal G. Some data quality issues at ClinicalTrials.gov. Trials. 2019 Jun 24;20(1):378. doi: 10.1186/s13063-019-3408-2. PMID: 31234923; PMCID: PMC6591874.

13. Pillamarapu M, Mohan A, Saberwal G. An analysis of deficiencies in the data of interventional drug trials registered with Clinical Trials Registry - India. Trials. 2019 Aug 28;20(1):535. doi: 10.1186/s13063-019-3592-0. PMID: 31455366; PMCID: PMC6712861.

14. Fleminger J, Goldacre B. Prevalence of clinical trial status discrepancies: A cross-sectional study of 10,492 trials registered on both ClinicalTrials.gov and the European Union Clinical Trials Register. PLoS One. 2018 Mar 7;13(3):e0193088. doi: 10.1371/journal.pone.0193088. PMID: 29513684; PMCID: PMC5841737.

15. Zarin DA, Tse T, Williams RJ, Carr S. Trial Reporting in ClinicalTrials.gov - The Final Rule. N Engl J Med. 2016 Nov 17;375(20):1998-2004. doi: 10.1056/NEJMsr1611785. Epub 2016 Sep 16. PMID: 27635471; PMCID: PMC5225905.

16. Kumari S, Mohan A, Saberwal G. Hidden duplicates: 10s or 100s of Indian trials, registered with ClinicalTrials.gov, have not been registered in India, as required by law. PLoS One. 2020 Jun 19;15(6):e0234925. doi: 10.1371/journal.pone.0234925. PMID: 32559240; PMCID: PMC7304601.

17. Alarcon-Ruiz CA, Roque-Roque JS, Heredia P, Gómez-Briceño AR, Quispe AM. Twenty-two years' experience registering trials in a low-middle income country: The Peruvian Clinical Trial Registry. J Evid Based Med. 2019 Aug;12(3):187-193. doi: 10.1111/jebm.12354. Epub 2019 Jun 18. PMID: 31215157.

18. Ranawaka UK, de Abrew A, Wimalachandra M, Samaranayake N, Goonaratna C. Ten years of clinical trial registration in a resource-limited setting: Experience of the Sri Lanka clinical trials registry. J Evid Based Med. 2018 Feb;11(1):46-50. doi: 10.1111/jebm.12284. Epub 2018 Jan 11. PMID: 29322622.

19. Krumholz HM, Ross JS, Presler AH, Egilman DS. What have we learnt from Vioxx? BMJ. 2007 Jan 20;334(7585):120-3. doi: 10.1136/bmj.39024.487720.68. PMID: 17235089; PMCID: PMC1779871.

20. Jureidini JN, McHenry LB, Mansfield PR. Clinical trials and drug promotion: Selective reporting of study 329. International Journal of Risk & Safety in Medicine. 2008;20: 73–81. doi:10.3233/JRS-2008-0426

21. WHO Technical Consultation on Clinical Trials Registration Standards. Geneva: World Health organization. 25-27 April 2005; Available: https://www.who.int/ictrp/news/ictrp_meeting_april2005_conclusions.pdf

22. ICTRP Search Portal [Internet]. [cited 21 Mar 2020]. Available: http://apps.who.int/trialsearch/

23. WHO | The WHO Registry Network [Internet]. [cited 1 Oct 2020]. Available: https://www.who.int/ictrp/network/en/

24. WHO | Partner Registries [Internet]. [cited 1 Oct 2020]. Available: https://www.who.int/ictrp/network/partner/en/

25. International Standards for Clinical Trial Registries – Version 3.0. Geneva: World Health Organization; 2018. License: CC BY-NC-SA 3.0 IGO

26. The-Clinical-Trial-Registry-of-the-Future-Proposal.pdf [Internet]. [cited 2020 Mar 22]. Available from: http://www.transceleratebiopharmainc.com/wp-content/uploads/2017/11/The-Clinical-Trial-Registry-of-the-Future-Proposal.pdf

27. WHO ICTRP Registry Network Meeting Summary Report. Geneva: World Health Organization; 2015. Available: https://www.who.int/ictrp/news/ICTRP_registry_network_meeting_report_2015_web.pdf?ua=1

28. Moja LP, Moschetti I, Nurbhai M, Compagnoni A, Liberati A, Grimshaw JM, Chan AW, Dickersin K, Krleza-Jeric K, Moher D, Sim I, Volmink J. Compliance of clinical trial registries with the World Health Organization minimum data set: a survey. Trials. 2009 Jul 22;10:56. doi: 10.1186/1745-6215-10-56. PMID: 19624821; PMCID: PMC2734552.

29. Ogino D, Takahashi K, Sato H. Characteristics of clinical trial websites: information distribution between ClinicalTrials.gov and 13 primary registries in the WHO registry network. Trials. 2014 Nov 5;15:428. doi: 10.1186/1745-6215-15-428. PMID: 25373358; PMCID: PMC4234832.

30. Miller JE, Wilenzick M, Ritcey N, Ross JS, Mello MM. Measuring clinical trial transparency: an empirical analysis of newly approved drugs and large pharmaceutical companies. BMJ Open. 2017 Dec 5;7(12):e017917. doi: 10.1136/bmjopen-2017-017917. PMID: 29208616; PMCID: PMC5728266.

31. Goldacre B. How to get all trials reported: audit, better data, and individual accountability. PLoS Med. 2015 Apr 14;12(4):e1001821. doi: 10.1371/journal.pmed.1001821. PMID: 25874719; PMCID: PMC4396123.

32. Our scoring method. [Internet]. [cited 21 Mar 2020]. Available: http://policyaudit.alltrials.net/our-scoring-method/

33. Goldacre B, Gray J. OpenTrials: towards a collaborative open database of all available information on all clinical trials. Trials. 2016 Apr 8;17:164. doi: 10.1186/s13063-016-1290-8. PMID: 27056367; PMCID: PMC4825083.

34. Azar M, Riehm KE, Saadat N, Sanchez T, Chiovitti M, Qi L, Rice DB, Levis B, Fedoruk C, Levis AW, Kloda LA, Kimmelman J, Benedetti A, Thombs BD. Evaluation of Journal Registration Policies and Prospective Registration of Randomized Clinical Trials of Nonregulated Health Care Interventions. JAMA Intern

Med. 2019 May 1;179(5):624-632. doi: 10.1001/jamainternmed.2018.8009. PMID: 30855655; PMCID: PMC6503638.

35. Miller J, Ross JS, Wilenzick M, Mello MM. Sharing of clinical trial data and results reporting practices among large pharmaceutical companies: cross sectional descriptive study and pilot of a tool to improve company practices. BMJ. 2019 Jul 10;366:l4217. doi: 10.1136/bmj.l4217. PMID: 31292127; PMCID: PMC6614834.

36. Jones CW, Safferman MR, Adams AC, Platts-Mills TF. Discrepancies between ClinicalTrials.gov recruitment status and actual trial status: a cross-sectional analysis. BMJ Open. 2017 Oct 11;7(10):e017719. doi: 10.1136/bmjopen-2017-017719. PMID: 29025842; PMCID: PMC5652524.

37. Chan AW, Song F, Vickers A, Jefferson T, Dickersin K, Gøtzsche PC, Krumholz HM, Ghersi D, van der Worp HB. Increasing value and reducing waste: addressing inaccessible research. Lancet. 2014 Jan 18;383(9913):257-66. doi: 10.1016/S0140-6736(13)62296-5. Epub 2014 Jan 8. PMID: 24411650; PMCID: PMC4533904.

38. Tharyan P. Ethics committees and clinical trials registration in India: opportunities, obligations, challenges and solutions. Indian J Med Ethics. 2007 Oct-Dec;4(4):168-9. doi: 10.20529/IJME.2007.066. PMID: 18630235.

39. Pandey A, Aggarwal A, Seth S, Maulik M, Bano R, Juneja A. Clinical Trials Registry--India: redefining the conduct of clinical trials. Indian J Cancer. 2008 Jul-Sep;45(3):79-82. doi: 10.4103/0019-509x.44060. PMID: 19018108.

40. Krleza-Jerić K. Clinical trial registration: the differing views of industry, the WHO, and the Ottawa Group. PLoS Med. 2005 Nov;2(11):e378. doi: 10.1371/journal.pmed.0020378. Epub 2005 Oct 18. PMID: 16221000; PMCID: PMC1255765.

41. WHO | WHO Data Set. In: WHO [Internet]. [cited 16 May 2019]. Available: http://www.who.int/ictrp/network/trds/en/

42. Potthast R, Vervölgyi V, McGauran N, Kerekes MF, Wieseler B, Kaiser T. Impact of inclusion of industry trial results registries as an information source for systematic reviews. PLoS One. 2014 Apr 17;9(4):e92067. doi: 10.1371/journal.pone.0092067. PMID: 24743113; PMCID: PMC3990559.

43. Harriman SL, Patel J. When are clinical trials registered? An analysis of prospective versus retrospective registration. Trials. 2016 Apr 15;17:187. doi: 10.1186/s13063-016-1310-8. PMID: 27079379; PMCID: PMC4832501.

44. Committee on Clinical Trial Registries; Board on Health Sciences Policy; Institute of Medicine. Developing a National Registry of Pharmacologic and Biologic Clinical Trials: Workshop Report. Washington (DC): National Academies Press (US); 2006. PMID: 26740992.

45. Williams RJ, Tse T, DiPiazza K, Zarin DA. Terminated Trials in the ClinicalTrials.gov Results Database: Evaluation of Availability of Primary Outcome Data and Reasons for Termination. PLoS One. 2015 May 26;10(5):e0127242. doi: 10.1371/journal.pone.0127242. PMID: 26011295; PMCID: PMC4444136.

46. Metathesaurus. [cited 22 Mar 2020]. Available: https://www.nlm.nih.gov/research/umls/knowledge_sources/metathesaurus/index.html

47. Council Post: Why An SSL Certificate Is Important For Your Company Website. [Internet]. [cited 25 Sep 2020]. Available: https://www.forbes.com/sites/forbestechcouncil/2018/05/18/why-an-ssl-certificate-is-important-for-your-company-website/#2181c3fb1dc3

48. Yadav P, Jaykaran, Chaudhari M, Saxena D, Kantharia ND. Clinical trials registered in clinical trial registry of India: A survey. J Pharmacol Pharmacother. 2011 Oct;2(4):289-92. doi: 10.4103/0976-500X.85953. PMID: 22025862; PMCID: PMC3198529.

49. ANZCTR [Internet]. [cited 25 Sep 2020]. Available: https://www.anzctr.org.au/crawl.aspx

50. Oxford recruits 2,700 virus patients for fast-growing drugs trial. Financial Times. [cited 25 Sep 2020]. Available: https://www.ft.com/content/f4e3055e-72e2-459d-ba66-05af7d2c8915

51. Clinical trials for a Covid-19 vaccine to begin in Rochester. WHAM. [cited 25 Sep 2020]. Available: https://13wham.com/news/local/clinical-trials-for-a-covid-19-vaccine-to-begin-in-rochester

52. COVID-19: Hundreds Of Clinical Trials Under Way In India, Many Lack Rigour, Say Experts. [cited 25 Sep 2020]. Available: https://www.indiaspend.com/covid-19-hundreds-of-clinical-trials-under-way-in-india-many-lack-rigour-say-experts/

53. Krleza-Jerić K, Chan AW, Dickersin K, Sim I, Grimshaw J, Gluud C. Principles for international registration of protocol information and results from human trials of health related interventions: Ottawa statement (part 1). BMJ. 2005 Apr 23;330(7497):956-8. doi: 10.1136/bmj.330.7497.956. Erratum in: BMJ. 2005 May 28;330(7502):1258. PMID: 15845980; PMCID: PMC556346.

54. AllTrials. All Trials Registered. All Results Reported. In: AllTrials [Internet]. [cited 16 May 2019]. Available: http://www.alltrials.net/

55. Miller JE, Korn D, Ross JS. Clinical trial registration, reporting, publication and FDAAA compliance: a cross-sectional analysis and ranking of new drugs approved by the FDA in 2012. BMJ Open. 2015 Nov 12;5(11):e009758. doi: 10.1136/bmjopen-2015-009758. PMID: 26563214; PMCID: PMC4654354.

56. ICMJE | Recommendations | Clinical Trials. [cited 10 Jun 2019]. Available: http://www.icmje.org/recommendations/browse/publishing-and-editorial-issues/clinical-trial-registration.html

57. Zarin DA, Tse T, Williams RJ, Rajakannan T. Update on Trial Registration 11 Years after the ICMJE Policy Was Established. N Engl J Med. 2017 Jan 26;376(4):383-391. doi: 10.1056/NEJMsr1601330. PMID: 28121511; PMCID: PMC5813248.

58. Clinical trials policy – Grant funding | Wellcome. [cited 30 Sep 2020]. Available: https://wellcome.org/grant-funding/guidance/clinical-trials-policy

59. Publishing Policies | Gates Open Research. [cited 30 Sep 2020]. Available: https://gatesopenresearch.org/about/policies#trialreg

60. FDAAA 801 and the Final Rule - ClinicalTrials.gov. [cited 25 Sep 2020]. Available: https://clinicaltrials.gov/ct2/manage-recs/fdaaa

Supporting information

S1 Table. Mapping to ISCTR. The ~~17~~ 14 features analyzed in ~~-~~this study map to the following nine sections of ISCTR: (i) Content, (ii) Quality and Validity, (iii) Accessibility, (iv) Unambiguous Identification, (v) Technical Capacity, (vi) Administration and Governance, (vii) The 24-field TRDS, (viii) Partner Registries, and (ix) Data Interchange Standards.

S2 Table. Data on six aspects of each registry. (a) ~~Time taken to obtain the results of a search, (b)~~ Total number of trials in the registry, (~~c~~b)~~)~~ Existence of a basic search function, (c~~d~~ and d~~e~~) Advanced search function – TRDS fields and Extra fields, and (e~~f~~) Data download options.

S3 Table. The list of the TRDS fields that are present in the Brief view and the Detailed view. The presence or absence of the field is indicated by a 1 or 0, respectively. The number of the sample trial used for each registry is also provided.

S4 Table. For each registry, a listing of the Extra fields in the Brief and Detailed views.

S5 Table. Data on multiple aspects of each registry. (a) whether the PI name is compulsory; (b) reason for the termination of a trial, and whether there is a drop-down menu of reasons; (c) audit trail; (d) health condition (e) SSL certificate, and (f) documentation.

Dear Editor,

Please find below out itemized responses to the reviewers' comments. We trust that they are satisfactory.

Sincerely,
Gayatri Saberwal

Response to Reviewers

REVIEWER #1: Overall this is a useful and very timely piece of work which, as the authors say, could trigger further work on registry assessment and a wider debate on how trial registries can both improve the features they offer and become more consistent. In general it is well written and well referenced, and is supported by a comprehensive set of detailed data as supplementary files. The authors acknowledge the limitations of their study and include a useful set of suggestions for an 'ideal registry' as an aspiration to work towards.

I have some reservations about some aspects of the paper, however, which I think detract from its overall quality – but which I hope can be easily rectified:
Authors: We thank the reviewer for the appreciative comments.

\- - - - - - - - - - - - - - - - -
1) I found the organisation of some of the material confused. In particular the very short methods section provides little detail about the 17 features selected as the basis of assessment, how and why they were selected, and by whom, and how decisions on weighting were made, and why 3 were not assessed. Later on, in table 2b and as a large part of the 'Discussion of specific features…' in Box 1, much of this material is covered, but I think it would have been simpler and more logical to bring these explanations together as part of an expanded methods section. Box 1 is embedded in the discussion but its content seems largely a justification of the scorecard's construction. The result is that the reader has to work harder than they should to understand how and why the scoring system was constructed.
Authors: We have reorganized the Methods section, and added further details. This includes portions from Box 1. We have also moved Table 2b to the methods section (where it is now Table 1) to make the rationale of the scorecard available upfront. Additionally, we wish to highlight that lines 128–130 describe how the authors selected the criteria for the scorecard, based on a review of the literature, but mainly focussing on the ISCTR guidelines.

\- - - - - - - - - - - - - - - - -
2) Similarly I think the results section could be better organised. Why not simply go through the results for each of the 14 areas assessed, noting at that point the median and the range of scores, techniques and difficulties in assessment, and possible caveats around the scores obtained? The current section provides useful tables and a brief summary, but much of the text is simply restating what was accessed. Would a simple pie chart be a useful way of summarising the total numbers data in table 1, to show the proportion of total registry entries included in each?
Authors: We have rewritten and reorganized the Results and Discussion. We have created the suggested pie chart, and have also presented other data from the erstwhile Table 1 as a figure.

\- - - - - - - - - - - - - - - - -
3) A minor point, but there 10 superscript references in Table 1, presumably to some explanatory notes about the data point presented, but I could not find any explanation for them, either in the main text or the supplementary material. They should either be removed or (better) the explanatory notes should be provided.

Authors: These notes were inadvertently left out due to the complications of submitting large tables in a particular format. They are visible in the revised Table.

- - - - - - - - - - - - - - - - -

4) I thought the discussion was a little timid. The work was done in early 2020, in the context of a pandemic that has dramatically underscored the need for good quality, consistent and easily available information from trial registries, partly to be able to track the numbers, types and results of trials relating to COVID-19, partly because public health decisions require a network of data sources at a global level and registries should be a key part of this. That point might have been worth including – improving trial registry systems has become more urgent!

Authors: We have rewritten the discussion, which include the following lines.

"The ongoing Covid-19 pandemic has forcefully brought home the need for high quality trial registries with information that is consistent, comprehensive and available in a user-friendly fashion. Billions of people need to be immediately protected from the virus, and large numbers of drugs and vaccines are in trials. There is world-wide interest in these trials, and information that is being tracked includes what is being trialled; where are these trials taking place; and what are the results of these trials? Each country needs to take public health decisions, which will evolve as trials running in different parts of the world yield results. Public trial registries are one of the fastest ways of communicating these results.

Further, the publicly available, freely accessible information in trial registries helps to build trust with the public [26,44]. Covid-19 trials have been among the fastest recruiting trials in history [50,51], and it is possible that the publicly available information in trial registries has helped many of the potential trial participants decide to enrol.

It is not just that everyone is interested in the positive outcomes of trials. For example, an inspection of the CTRI records of hundreds of covid-19 trials being run in India has thrown up quality issues in almost all of them. Based on negative publicity, the government has taken action in some cases [52]."

- - - - - - - - - - - - - - - - -

5) Similarly, although there is a general sentiment expressed that registries should improve, there were no concrete suggestions as to how this might be achieved or who needs to be involved, e.g. by greater collaboration between registries, perhaps orchestrated by the WHO, or by using the influence of funders and publishers to re-iterate the need for greater consistency. Are some of the aspects that were assessed easier to improve than others? If so how could they be progressed? Should there be a web page with a regularly updated 'score card' for the trial registries? I appreciate this was an initial survey but I think it might have been useful to venture, if only briefly, into this area in the discussion.

Authors: We have added the following lines to the Results and Discussion:

There is a long history of various stakeholders arguing for the need to improve registries and the quality of trial registration. Examples include academics and health activists [53–55], journals (ICMJE) [56], WHO [41], registry managers [57], funders [58,59] and governments [60]. Each of these efforts has led to some improvements in the number and quality of trial records hosted by registries. However none of them has led to a perfect set of records. It is likely that the only way this will be achieved is if all stakeholders continue to apply pressure on the registries. Studies such as this one help to highlight deficiencies, which adds to the other efforts aimed at improving registries. Further, the authors would welcome other researchers' efforts to create and update a website that lists the scorecard, with periodic updates. Should such a website not be created by any other group, the authors intend to re-evaluate the registries' performance on the scorecard every few years.

- - - - - - - - - - - - - - - - - -

6) Another issue largely missing from the discussion: the authors mention that registries have many different types of users – researchers, clinicians, members of the public, data scientists, etc. I wonder if this should therefore lead to different scoring systems – perhaps with different weightings and / or items – for each of those major user groups. Those could provide additional insight into the strengths and weakness of different repositories, and thus more clearly identify areas of improvement, but could also be consolidated into an overall score if desired. For example, although the authors state their assumption is that most users would not have the technical expertise to use APIs, and / or scraping and crawling systems to retrieve data, the integration of trial registries with other data systems, and thus the ability to support bulk download by machines, is becoming increasingly important. I would have liked to have seen this aspect more explicitly included in any 'to do list' of possible future assessments, along with considerations of data quality, completeness, and the support for reporting results

Authors: In the Discussion, while enumerating the various kinds of users of registry data, we have stated that "Other categories of users, such as medical professionals, patients, trial sponsors, policy makers, data scientists and so on, may wish to alter the assessed features, or the scoring, in order to rank the registries according to their priorities. For instance, a data scientist would be very appreciative of ANZCTR, which specifically enables web crawling of its records [49]. Furthermore, the managers of other registries, either public or private, and either based on the data in the PR+ or not, may be interested in the results of this study.". We do not feel confident of creating different scoring systems. Ideally, this should be done by polling at least a few individuals in each category of users, and we would find it extremely challenging to do this in India. As such, any additional scoring system that we developed would be based on unvalidated assumptions, and would be unconvincing, even to us.

- - - - - - - - - - - - - - - - - -

7) There is a minor but distracting typo in the first paragraph of the Results section (5,72,901)

Authors: We had used the Indian system. We have corrected this to 572,901.

- - - - - - - - - - - - - - - - - -

Having listed all of the points above I would re-iterate that overall I think the paper is useful and should be published. The points are offered as suggestions for possible improvement.

REVIEWER #2:

The concept is interesting but needs to be re-written.

1. The paper should first start with a good explanation of the origins of the ISCTR. For example, "following the Ministerial Summit on Health Research that took place in Mexico City, Mexico, in November 2004, participants called for the WHO to facilitate the establishment of: "a network of international clinical trials registers to ensure a single point of access and the unambiguous identification of trials".

   Authors: We have rewritten the Introduction to include these events and further details of the ICTRP.

   - - - - - - - - - - - - - - - - - -

2. https://www.who.int/ictrp/about/en/ The authors need to be more complete in explaining the WHO registry network including primary vs partner registries as well as data providers; the differences of each. Then as it relates to registries what kind of papers have been published; findings; some of this is introduced at a high level in the discussion section which belongs in the introduction.

   Authors: We have include these points in the Introduction.

- - - - - - - - - - - - - - - - - -

3. The authors are not clear in their terminology (for example, versions vs features). There is reference to WHO's 24-field Trial Registration Data Set vs 17 features vs 14 features selected by the authors; there is reference to the 9 standards; hence it is not clear how these "concepts" inter-relate (24 vs 17 vs 9; data set vs standards) and why the authors selected 14 features (which is perhaps more attributes than features).
   Authors: We have now ensured the following:
   (a) the word 'section' only refers to one or more of the nine sections of the ISCTR;
   (b) the word 'feature' only refers to one or more of the 14 features of each registry that are the focus of this study;
   (c) since the different registries may have different variants of each 'feature', we use the word 'variation' in this context.
   We have also included this list as a Box within Methods so that readers have no confusion regarding the terminology used.

- - - - - - - - - - - - - - - - - -

4. How does WHO refer to author defined "features".
   Authors: As described in Methods, the 'features' defined in this study have been compiled by the authors from different sources, including ISCTR. Therefore ISCTR discusses some, but not all, of these features directly. However, each feature maps to one or more standards set forth in ISCTR.

- - - - - - - - - - - - - - - - - -

5. The score card is hard to follow; the scoring is not understandable (for example, "for features with multiple variants, the score ranges from 1 to 5" is not clear and then how scores were calculated: as in the case of chCTR for advanced search fields TRDS a score of 17 was assigned).
   Authors: We have reorganized the Methods section, and added further details. This includes portions from Box 1. We have also moved Table 2b to the methods section (where it is now Table 1) to make the rationale of the scorecard available upfront. Further, we have rewritten the Results and Discussion to include a more detailed analysis of the findings. We hope this alleviates the confusion around the scorecard.

- - - - - - - - - - - - - - - - - -

6. The meaning of the scorecard is not clear; the interpretation of the findings are inconsistent and leaves the reader bewildered.
   Authors: We regret that the first version of the manuscript was so confusing. Please refer to our response to the comment before this (Comment 5).

- - - - - - - - - - - - - - - - - -

7. Please see specific comments in the attached word document.

Authors: Please find below a response to each of the comments in the manuscript file, which we have numbered from 7.1 to 7.44). In each case we have referenced the line in the original pdf, where the reviewer's comment has been taken from.

- - - - - - - - - - - - - - - - - -

7.1 Perhaps to elaborate on the 14 features briefly: what do they cover off on. (Line 31)
Authors: This has been done. **[Lines 32–35, and 41–44 of the revised manuscript]**

- - - - - - - - - - - - - - - - - -

7.2 Would not include limitations here. Only in the body of the paper under the proper section. (Line 42)
Authors: This has been done.

- - - - - - - - - - - - - - - - - -
7.3 New information should not be introduced in the conclusion; rather include in the results section. (Line 51)
Authors: This has been done. [Lines 587–589 of the revised manuscript]

- - - - - - - - - - - - - - - - - -
7.4 such as? Elaborate (Line 53)
Authors: This has been done. [Lines 63–68 of the revised manuscript]

- - - - - - - - - - - - - - - - - -
7.5 elaborate on types of comments and analyses. (Line 67)
Authors: This has been done. **[Lines 72–76 of the revised manuscript]**

- - - - - - - - - - - - - - - - - -
7.6 awkwardly written. "Set up to facilitate"? The ICTRP was designed to help facilitate. (Line 68)
Authors: This has been reworded. [Lines 81–85 of the revised manuscript]

- - - - - - - - - - - - - - - - - -
7.7 replace wording with "not an ICTRP recognized registry"... (Line 71)
Authors: This has been reworded. [Lines 85–88 of the revised manuscript]

- - - - - - - - - - - - - - - - - -
7.8 why were the other partner registries not included? or at least some of the other partner registries? (Line 74)
Authors: We have not included *any* partner registry in our analysis. This issue has been covered in more detailed now. [Lines 88–93 of the revised manuscript]

- - - - - - - - - - - - - - - - - -
7.9 Write as Primary Registries Plus (PR+) (Line 74)
Authors: This has been done. [Lines 92–93 of the revised manuscript]

- - - - - - - - - - - - - - - - - -
7.10 Define the nine sections... (Line 77)
Authors: This has been done. [Lines 97–100 of the revised manuscript]

- - - - - - - - - - - - - - - - - -
7.11 Recommend using 3rd person objective. Not "we" (Line 77)
Authors: It would be extremely challenging to rewrite the Methodology in 3rd person. However, we have ascertained that 'we' is used in the Methods' section, in articles that have appeared in well-known journals including PLOS ONE such as:
https://www.bmj.com/content/362/bmj.k3218
https://trialsjournal.biomedcentral.com/articles/10.1186/1745-6215-15-428
https://journals.plos.org/plosone/article?id=10.1371/journal.pone.0193088#sec006
We hope that it is alright if we leave the construction as it is.

- - - - - - - - - - - - - - - - - -
7.12 not clear: using terms like versions, features, sections - difficult for the reader to follow (Line 83)

Authors: We have addressed this in our response to this Reviewer's point 3.

- - - - - - - - - - - - - - - - - -
7.13 important to note that not all fields in clinicaltrial.gov are mandatory. is this the case in other registries as well? (Line 96)
Authors: Yes, it is true that all fields are not mandatory in any registry. However we are only examining the *presence* of certain fields, not whether trialists have filled each of them.

- - - - - - - - - - - - - - - - - -
7.14 Authors? (Line 98)
Authors: We have rewritten this sentence and it now reads, 'All analyses were performed by one author (NV) and verified by the other (GS)."

- - - - - - - - - - - - - - - - - -
7.15 Is this part of Methods (Line 102)
Authors: We have formatted the manuscript to more clearly demarcate the different levels of headings.

- - - - - - - - - - - - - - - - -
7.16 needs to be defined "multiple variants, score ranges" (Line 106)
Authors: We have defined the variants and the score ranges in Table 1.

- - - - - - - - - - - - - - - - - -
7.17 difficult to follow; confusing (Line 107)
Authors: We have moved Table 2b to the methods section (where it is now Table 1) to make the rationale of the scorecard available upfront. We have also illustrated our scoring system with examples early in the Methods section. Additionally, we wish to highlight that lines 128–130 describe how the authors selected the criteria for the scorecard, based on literature review but mainly focussing on the ISCTR guidelines.

- - - - - - - - - - - - - - - - - -
7.18 perhaps to refer to as "attributes"? (Line 117)
Authors: We have determined that 'feature' and 'attribute' are synonyms. Since we have used 'features' throughout the manuscript, we preferred to stick to it. Also, as detailed in our response to the comment 7.12, above, we have rationalized our use of the word 'features' so that there is no confusion over its usage.

- - - - - - - - - - - - - - - - - -
7.19 why 14 of the 17? (Line 118)
Authors: While revising the manuscript, we have removed the three features that were not assessed quantitatively. This leaves 14 features, all of which are in the scorecard. We believe that these changes have removed room for confusion on this point, and improved the readability of the paper.

- - - - - - - - - - - - - - - - - -
7.20 we went from 14 of 17 to 24. Not sure how this ties back to the 9 standards introduced under Methods (Line 120)
Authors: As mentioned in point 7.19, above, we have removed the three features that were assessed quantitatively, and the final number is 14.
Regarding the number 24: As described in the manuscript, one of the 14 features is the WHO's Trial Registration Data Set, or TRDS, which in turn is composed of 24 distinct fields (as defined by ISCTR).

- - - - - - - - - - - - - - - - -
7.21 weight not weightage (Line 123)
Authors: This has been rewritten. [Lines 229–233 of the revised manuscript]

- - - - - - - - - - - - - - - - -
7.22 why is this a feature of the "registry" vs what is required by government in the local country. (Line 133)
Authors: In order to avoid possible confusion, we have rephrased the sentence to: "As a first step, it is important to know how many records the database holds. This number should be readily available, and we have therefore analysed the ease of accessing it."

- - - - - - - - - - - - - - - - -
7.23 why were these two subjects highlighted especially as they were not noteworthy or substantive. (Line 136)
Authors: We have deleted these two features now.

- - - - - - - - - - - - - - - - -
7.24 is this a server issue; connectivity; (Line 140)
Authors: We do not know the reason for the lag in loading RPCEC results. It seems to be at the RPCEC end, since other registries gave us no problem. In any case, we have deleted the entire feature now.

- - - - - - - - - - - - - - - - -
7.25 would think this is an important aspect to score; the logic to not score this as half of the registries did not contain is a shortcoming of the analysis as registries that do not have this attribute should receive a lower rating. Completion of variables is less about the registry vs the owner of the "data" (sponsor). (Line 140)
Authors: We have now included in the Results and Discussion our analysis of whether the registry provides the reason for trial termination. However, the information pertaining to this feature is already present in the Extra fields. Hence we have not scored this features separately, since that would result in it being double counted.

- - - - - - - - - - - - - - - - -
7.26 why these attributes? Why is SSL important? (Line 154)
Authors: We have now included a more detailed description of why SSL is important and why we have included this feature. [Lines 471–481 of the revised manuscript]

- - - - - - - - - - - - - - - - -
7.27 this is the sponsor's responsibility: QC (Line 166)
Authors: In the Results and Discussion section we have now described why even though QC is the sponsor's responsibility it does not always do this, and how it is the duty of the registry to facilitate higher quality registrations. [Lines 496–501 of the revised manuscript]

- - - - - - - - - - - - - - - - -
7.28 these points should have been provided in the introduction. (Line 181)
Authors: We have now included these points in the Introduction.

- - - - - - - - - - - - - - - - -
7.29 there is no enough discussion on what has been done, why there is a gap and how this fills the gap in a meaningful way. (Line 189)

Authors: We have rewritten the Introduction and have discussed the work done so far, and how our study fills a lacuna in the analysis and reporting of registries' performance.

- - - - - - - - - - - - - - - - -

7.30 why is this ideal? (Line 195)

Authors: As described in the manuscript, we propose an 'interim ideal' registry based on the features that we have assessed. That it is a limited goal on the way to achieving everything that ISCTR requires. And it is the 'ideal' from amongst the various options that one or more of the registries are already using.

- - - - - - - - - - - - - - - - -

7.31 seems per line 180 to 189 there have been other studies. not clear. (Line 215)

Authors: We believe that the revised manuscript addresses this concern.

- - - - - - - - - - - - - - - - -

7.32 how was this impartial? (Line 216)

Authors: Primarily based on the recommendations of ISCTR, we determined which features of the registries to assess. All scoring rationales were also based on the minimum standards outlined in the ISCTR, and recommendations from earlier studies in our literature survey. We believe that our scoring is impartial since this protocol rules out scoring that may be biased in favour of, or against, any particular registry.

- - - - - - - - - - - - - - - - -

7.33 over reach as this is a subjective statement; only state represents the authors' perspective not that of others. (Line 220)

Authors: We have changed this sentence, which now reads "The scorecard above analyses features that are of interest to the authors and, by extension, possibly to other researchers concerned with the health of the trial ecosystem overall."

- - - - - - - - - - - - - - - - -

7.34 not clear (Line 222)

Authors: We have rephrased this to the following: "Other categories of users, such as medical professionals, patients, trial sponsors, policy makers, data scientists and so on, may wish to alter the assessed features, or the scoring, in order to rank the registries according to their priorities."

- - - - - - - - - - - - - - - - -

7.35 registries cannot be interested in something; only those who work on registries (Line 224)

Authors: We have rephrased the sentence to the following: "Further, the managers of other registries, either public or private, and either based on the data in the PR+ or not, may be interested in the results of this analysis."

- - - - - - - - - - - - - - - - -

7.36 less about the registry and more about how users utilize unless all fields are required by the registry. (Line 230)

Authors: We are not sure that we have understood this question. It is true that in no registry are all fields mandatory. Thus it would not be advisable to evaluate the quality of registration, and of information in a particular field, by comparisons across registries. However, as we have described in our response to Reviewer comment 7.13, we are only examining the *presence* of certain fields, not whether trialists have filled each of them.

- - - - - - - - - - - - - - - - -

7.37 need to write using English proper language fit for scientific publication (Line 238)
Authors: We have rephrased the sentence to the following: "As noted above, 41% of the records are held in the other PR+, and they need to be examined as well."

- - - - - - - - - - - - - - - - -
7.38 Define (Page 19, Box 1)
Authors: We have described our assessment of this feature in greater detail in the revised manuscript.

- - - - - - - - - - - - - - - - -
7.39 more importantly why do registries include something not recommended by ISCTR? (Page 20, Box 1)
Authors: We have added the following lines to the manuscript: "There may be a range of reasons for including these Extra fields. For instance, India had been criticized for the lack of appropriate oversight to ensure the ethical conduct of trials, and therefor CTRI asked trialists for details of the ethics committee even before ISCTR required this information [38,39]. Also, there have been demands from the Cochrane collaboration, and many other individuals and groups, to include several additional items in the ISCTR list, which WHO has not agreed to. It is alleged that the recommended list is closer to what industry demanded [40]. As such, although ISCTR may not list every field that many people believed to be essential, managers of particular registries may have chosen to list some of them."

- - - - - - - - - - - - - - - - -
7.40 Restructure: what does WHO require; what do registries do; where is the difference and impact on value of registries. (Page 20, Box1)
Authors: In each section of the Results and Discussion, we provide an introductory line, then the results, then the discussion. We have done this, incorporating the Reviewer's points in this section.

- - - - - - - - - - - - - - - - -
7.41 is this an author term? It is a prospective trial even if data is only entered after the study starts. (Page 21, Box1)
Authors: 'Retrospective trials' is used by other researchers – it is not our term. Nevertheless, earlier in the manuscript, we have added an explanatory line "Trials may be registered either prospectively or retrospectively, that is before the enrolment of the first participant or after." Also, in order to avoid ambiguity, we have rephrased this sentence as follows: "Further, flagging retrospectively registered ones may shame the registrants into registering prospectively in future".

- - - - - - - - - - - - - - - - -
7.42 what were the findings? (Page 21, Box1)
Authors: We have revised the text to include the findings of the analysis.

- - - - - - - - - - - - - - - - -
7.43 how was 3 assigned out of 5: what does a value of 1 or 2 or 3 or 4 or 5 represent? (Page 8, Table 2a)
Authors: As explained in the response to the Reviewer's comment 5, we have moved Table 2b to the methods section (where it is now Table 1) to make the rationale of the scorecard available upfront. We hope this will alleviate the confusion regarding scoring.

- - - - - - - - - - - - - - - - -
7.44 11? 17? thought scores were 1 to 5. (Page 8, Table 2a)

Authors: Please refer our response to the previous point (Reviewer's comment 7.43)

- - - - - - - - - - - - - - - - - -

8. The topic is interesting but the paper needs to be rewritten and the concept of a scorecard has to be rethought to ensure it has logical relevance to the reader, that the scoring is understandable and can be interpreted thereby leading to actionable insights.

Authors: We have rewritten and reorganized the manuscript, and we hope that these changes address the reviewer's concerns.

- - - - - - - - - - - - - - - - - -

9. The paper has to be placed in context of other relevant studies completed to date.

Authors: We have rewritten the Introduction to address this concern.
