# Supplementary material for: A comparative analysis of important public clinical trial registries, and a proposal for an interim ideal one
Source: PLoS One. 2021 May 11;16(5):e0251191. doi: 10.1371/journal.pone.0251191 (PMC8112656; doi:10.1371/journal.pone.0251191)
Supplement: Attachment — Submitted filename: Response to reviewers.docx [file pone.0251191.s007.docx]

Dear Editor,

Please find below out itemized responses to the reviewers’ comments. We trust that they are satisfactory.

Sincerely,

Gayatri Saberwal

**Response to Reviewers**

**REVIEWER #1**: Overall this is a useful and very timely piece of work which, as the authors say, could trigger further work on registry assessment and a wider debate on how trial registries can both improve the features they offer and become more consistent. In general it is well written and well referenced, and is supported by a comprehensive set of detailed data as supplementary files. The authors acknowledge the limitations of their study and include a useful set of suggestions for an 'ideal registry' as an aspiration to work towards.

I have some reservations about some aspects of the paper, however, which I think detract from its overall quality – but which I hope can be easily rectified:
**Authors: We thank the reviewer for the appreciative comments.**

- - - - - - - - - - - - - - - - - -
1) I found the organisation of some of the material confused. In particular the very short methods section provides little detail about the 17 features selected as the basis of assessment, how and why they were selected, and by whom, and how decisions on weighting were made, and why 3 were not assessed. Later on, in table 2b and as a large part of the 'Discussion of specific features…' in Box 1, much of this material is covered, but I think it would have been simpler and more logical to bring these explanations together as part of an expanded methods section. Box 1 is embedded in the discussion but its content seems largely a justification of the scorecard's construction. The result is that the reader has to work harder than they should to understand how and why the scoring system was constructed.
**Authors: We have reorganized the Methods section, and added further details. This includes portions from Box 1. We have also moved Table 2b to the methods section (where it is now Table 1) to make the rationale of the scorecard available upfront. Additionally, we wish to highlight that lines 128–130 describe how the authors selected the criteria for the scorecard, based on a review of the literature, but mainly focussing on the ISCTR guidelines.**

- - - - - - - - - - - - - - - - - -
2) Similarly I think the results section could be better organised. Why not simply go through the results for each of the 14 areas assessed, noting at that point the median and the range of scores, techniques and difficulties in assessment, and possible caveats around the scores obtained? The current section provides useful tables and a brief summary, but much of the text is simply restating what was accessed. Would a simple pie chart be a useful way of summarising the total numbers data in table 1, to show the proportion of total registry entries included in each?
**Authors: We have rewritten and reorganized the Results and Discussion. We have created the suggested pie chart, and have also presented other data from the erstwhile Table 1 as a figure.**

- - - - - - - - - - - - - - - - - -
3) A minor point, but there 10 superscript references in Table 1, presumably to some explanatory notes about the data point presented, but I could not find any explanation for them, either in the main text or the supplementary material. They should either be removed or (better) the explanatory notes should be provided.
**Authors: These notes were inadvertently left out due to the complications of submitting large tables in a particular format. They are visible in the revised Table.**

- - - - - - - - - - - - - - - - - -
4) I thought the discussion was a little timid. The work was done in early 2020, in the context of a pandemic that has dramatically underscored the need for good quality, consistent and easily available information from trial registries, partly to be able to track the numbers, types and results of trials relating to COVID-19, partly because public health decisions require a network of data sources at a global level and registries should be a key part of this. That point might have been worth including – improving trial registry systems has become more urgent!
**Authors: We have rewritten the discussion, which include the following lines.**

**“The ongoing Covid-19 pandemic has forcefully brought home the need for high quality trial registries with information that is consistent, comprehensive and available in a user-friendly fashion. Billions of people need to be immediately protected from the virus, and large numbers of drugs and vaccines are in trials. There is world-wide interest in these trials, and information that is being tracked includes what is being trialled; where are these trials taking place; and what are the results of these trials? Each country needs to take public health decisions, which will evolve as trials running in different parts of the world yield results. Public trial registries are one of the fastest ways of communicating these results.

Further, the publicly available, freely accessible information in trial registries helps to build trust with the public [26,44]. Covid-19 trials have been among the fastest recruiting trials in history [50,51], and it is possible that the publicly available information in trial registries has helped many of the potential trial participants decide to enrol.

It is not just that everyone is interested in the positive outcomes of trials. For example, an inspection of the CTRI records of hundreds of covid-19 trials being run in India has thrown up quality issues in almost all of them. Based on negative publicity, the government has taken action in some cases [52].”**

- - - - - - - - - - - - - - - - - -
5) Similarly, although there is a general sentiment expressed that registries should improve, there were no concrete suggestions as to how this might be achieved or who needs to be involved, e.g. by greater collaboration between registries, perhaps orchestrated by the WHO, or by using the influence of funders and publishers to re-iterate the need for greater consistency. Are some of the aspects that were assessed easier to improve than others? If so how could they be progressed? Should there be a web page with a regularly updated 'score card' for the trial registries? I appreciate this was an initial survey but I think it might have been useful to venture, if only briefly, into this area in the discussion.
**Authors: We have added the following lines to the Results and Discussion:**

**There is a long history of various stakeholders arguing for the need to improve registries and the quality of trial registration. Examples include academics and health activists [53–55], journals (ICMJE) [56], WHO [41], registry managers [57], funders [58,59] and governments [60]. Each of these efforts has led to some improvements in the number and quality of trial records hosted by registries. However none of them has led to a perfect set of records. It is likely that the only way this will be achieved is if all stakeholders continue to apply pressure on the registries. Studies such as this one help to highlight deficiencies, which adds to the other efforts aimed at improving registries. Further, the authors would welcome other researchers’ efforts to create and update a website that lists the scorecard, with periodic updates. Should such a website not be created by any other group, the authors intend to re-evaluate the registries’ performance on the scorecard every few years.**

- - - - - - - - - - - - - - - - - -
6) Another issue largely missing from the discussion: the authors mention that registries have many different types of users – researchers, clinicians, members of the public, data scientists, etc. I wonder if this should therefore lead to different scoring systems – perhaps with different weightings and / or items – for each of those major user groups. Those could provide additional insight into the strengths and weakness of different repositories, and thus more clearly identify areas of improvement, but could also be consolidated into an overall score if desired. For example, although the authors state their assumption is that most users would not have the technical expertise to use APIs, and / or scraping and crawling systems to retrieve data, the integration of trial registries with other data systems, and thus the ability to support bulk download by machines, is becoming increasingly important. I would have liked to have seen this aspect more explicitly included in any 'to do list' of possible future assessments, along with considerations of data quality, completeness, and the support for reporting results
**Authors: In the Discussion, while enumerating the various kinds of users of registry data, we have stated that “Other categories of users, such as medical professionals, patients, trial sponsors, policy makers, data scientists and so on, may wish to alter the assessed features, or the scoring, in order to rank the registries according to their priorities. For instance, a data scientist would be very appreciative of ANZCTR, which specifically enables web crawling of its records [49]. Furthermore, the managers of other registries, either public or private, and either based on the data in the PR+ or not, may be interested in the results of this study.”. We do not feel confident of creating different scoring systems. Ideally, this should be done by polling at least a few individuals in each category of users, and we would find it extremely challenging to do this in India. As such, any additional scoring system that we developed would be based on unvalidated assumptions, and would be unconvincing, even to us.**

- - - - - - - - - - - - - - - - - -
7) There is a minor but distracting typo in the first paragraph of the Results section (5,72,901)
**Authors: We had used the Indian system. We have corrected this to 572,901**.

- - - - - - - - - - - - - - - - - -

Having listed all of the points above I would re-iterate that overall I think the paper is useful and should be published. The points are offered as suggestions for possible improvement.

**REVIEWER #2:**

The concept is interesting but needs to be re-written.

1. The paper should first start with a good explanation of the origins of the ISCTR. For example, "following the Ministerial Summit on Health Research that took place in Mexico City, Mexico, in November 2004, participants called for the WHO to facilitate the establishment of: "a network of international clinical trials registers to ensure a single point of access and the unambiguous identification of trials".

**Authors: We have rewritten the Introduction to include these events and further details of the ICTRP.**

- - - - - - - - - - - - - - - - - -

2. <https://www.who.int/ictrp/about/en/> The authors need to be more complete in explaining the WHO registry network including primary vs partner registries as well as data providers; the differences of each. Then as it relates to registries what kind of papers have been published; findings; some of this is introduced at a high level in the discussion section which belongs in the introduction.

**Authors: We have include these points in the Introduction.**

- - - - - - - - - - - - - - - - - -

3. The authors are not clear in their terminology (for example, versions vs features). There is reference to WHO's 24-field Trial Registration Data Set vs 17 features vs 14 features selected by the authors; there is reference to the 9 standards; hence it is not clear how these "concepts" inter-relate (24 vs 17 vs 9; data set vs standards) and why the authors selected 14 features (which is perhaps more attributes than features).

**Authors: We have now ensured the following:**

**(a) the word ‘section’ only refers to one or more of the nine sections of the ISCTR;**

**(b) the word ‘feature’ only refers to one or more of the 14 features of each registry that are the focus of this study;**

**(c) since the different registries may have different variants of each ‘feature’, we use the word ‘variation’ in this context.**

**We have also included this list as a Box within Methods so that readers have no confusion regarding the terminology used.**

- - - - - - - - - - - - - - - - - -

4. How does WHO refer to author defined "features".

**Authors: As described in Methods, the ‘features’ defined in this study have been compiled by the authors from different sources, including ISCTR. Therefore ISCTR discusses some, but not all, of these features directly. However, each feature maps to one or more standards set forth in ISCTR.**

- - - - - - - - - - - - - - - - - -

5. The score card is hard to follow; the scoring is not understandable (for example, "for features with multiple variants, the score ranges from 1 to 5" is not clear and then how scores were calculated: as in the case of chCTR for advanced search fields TRDS a score of 17 was assigned).

**Authors: We have reorganized the Methods section, and added further details. This includes portions from Box 1. We have also moved Table 2b to the methods section (where it is now Table 1) to make the rationale of the scorecard available upfront. Further, we have rewritten the Results and Discussion to include a more detailed analysis of the findings. We hope this alleviates the confusion around the scorecard.**

- - - - - - - - - - - - - - - - - -

6. The meaning of the scorecard is not clear; the interpretation of the findings are inconsistent and leaves the reader bewildered.

**Authors: We regret that the first version of the manuscript was so confusing. Please refer to our response to the comment before this (Comment 5).**

- - - - - - - - - - - - - - - - - -

7. Please see specific comments in the attached word document.

**Authors: Please find below a response to each of the comments in the manuscript file, which we have numbered from 7.1 to 7.44). In each case we have referenced the line in the original pdf, where the reviewer’s comment has been taken from.**

- - - - - - - - - - - - - - - - - -

7.1 Perhaps to elaborate on the 14 features briefly: what do they cover off on. (Line 31)

**Authors: This has been done**. **[Lines 32–35, and 41–44 of the revised manuscript]**

- - - - - - - - - - - - - - - - - -

7.2 Would not include limitations here. Only in the body of the paper under the proper section. (Line 42)

**Authors: This has been done.**

- - - - - - - - - - - - - - - - - -

7.3 New information should not be introduced in the conclusion; rather include in the results section. (Line 51)

**Authors: This has been done. [Lines 587–589 of the revised manuscript]**

- - - - - - - - - - - - - - - - - -

7.4 such as? Elaborate (Line 53)

**Authors: This has been done. [Lines 63–68 of the revised manuscript]**

- - - - - - - - - - - - - - - - - -

7.5 elaborate on types of comments and analyses. (Line 67)

**Authors: This has been done**. **[Lines 72–76 of the revised manuscript]**

- - - - - - - - - - - - - - - - - -

7.6 awkwardly written. "Set up to facilitate"? The ICTRP was designed to help facilitate. (Line 68)

**Authors: This has been reworded. [Lines 81–85 of the revised manuscript]**

- - - - - - - - - - - - - - - - - -

7.7 replace wording with "not an ICTRP recognized registry"... (Line 71)

**Authors: This has been reworded. [Lines 85–88 of the revised manuscript]**

- - - - - - - - - - - - - - - - - -

7.8 why were the other partner registries not included? or at least some of the other partner registries? (Line 74)

**Authors: We have not included *any* partner registry in our analysis. This issue has been covered in more detailed now****. [Lines 88–93 of the revised manuscript]**

- - - - - - - - - - - - - - - - - -

7.9 Write as Primary Registries Plus (PR+) (Line 74)

**Authors: This has been done. [Lines 92–93 of the revised manuscript]**

- - - - - - - - - - - - - - - - - -

7.10 Define the nine sections... (Line 77)

**Authors: This has been done. [Lines 97–100 of the revised manuscript]**

- - - - - - - - - - - - - - - - - -

7.11 Recommend using 3rd person objective. Not "we" (Line 77)

**Authors:** **It would be extremely challenging to rewrite the Methodology in 3rd person. However, we have ascertained that 'we' is used in the Methods' section, in articles that have appeared in well-known journals including PLOS ONE such as:**

**https://www.bmj.com/content/362/bmj.k3218**

**https://trialsjournal.biomedcentral.com/articles/10.1186/1745-6215-15-428 https://journals.plos.org/plosone/article?id=10.1371/journal.pone.0193088#sec006**

**We hope that it is alright if we leave the construction as it is.**

- - - - - - - - - - - - - - - - - -

7.12 not clear: using terms like versions, features, sections - difficult for the reader to follow (Line 83)

**Authors: We have addressed this in our response to this Reviewer’s point 3.**

- - - - - - - - - - - - - - - - - -

7.13 important to note that not all fields in clinicaltrial.gov are mandatory. is this the case in other registries as well? (Line 96)

**Authors: Yes, it is true that all fields are not mandatory in any registry. However we are only examining the *presence* of certain fields, not whether trialists have filled each of them.**

- - - - - - - - - - - - - - - - - -

7.14 Authors? (Line 98)

**Authors: We have rewritten this sentence and it now reads, ‘All analyses were performed by one author (NV) and verified by the other (GS).”**

- - - - - - - - - - - - - - - - - -

7.15 Is this part of Methods (Line 102)

**Authors: We have formatted the manuscript to more clearly demarcate the different levels of headings.**

- - - - - - - - - - - - - - - - -

7.16 needs to be defined "multiple variants, score ranges" (Line 106)

**Authors: We have defined the variants and the score ranges in Table 1.**

- - - - - - - - - - - - - - - - - -

7.17 difficult to follow; confusing (Line 107)

**Authors: We have moved Table 2b to the methods section (where it is now Table 1) to make the rationale of the scorecard available upfront. We have also illustrated our scoring system with examples early in the Methods section. Additionally, we wish to highlight that lines 128–130 describe how the authors selected the criteria for the scorecard, based on literature review but mainly focussing on the ISCTR guidelines.**

- - - - - - - - - - - - - - - - - -

7.18 perhaps to refer to as "attributes"? (Line 117)

**Authors: We have determined that ‘feature’ and ‘attribute’ are synonyms. Since we have used ‘features’ throughout the manuscript, we preferred to stick to it. Also, as detailed in our response to the comment 7.12, above, we have rationalized our use of the word ‘features’ so that there is no confusion over its usage.**

- - - - - - - - - - - - - - - - - -

7.19 why 14 of the 17? (Line 118)

**Authors: While revising the manuscript, we have removed the three features that were not assessed quantitatively. This leaves 14 features, all of which are in the scorecard. We believe that these changes have removed room for confusion on this point, and improved the readability of the paper.**

- - - - - - - - - - - - - - - - - -

7.20 we went from 14 of 17 to 24. Not sure how this ties back to the 9 standards introduced under Methods (Line 120)

**Authors: As mentioned in point 7.19, above, we have removed the three features that were assessed quantitatively, and the final number is 14.**

**Regarding the number 24: As described in the manuscript, one of the 14 features is the WHO’s Trial Registration Data Set, or TRDS, which in turn is composed of 24 distinct fields (as defined by ISCTR).**

- - - - - - - - - - - - - - - - - -

7.21 weight not weightage (Line 123)

**Authors: This has been rewritten. [Lines 229–233 of the revised manuscript]**

- - - - - - - - - - - - - - - - - -

7.22 why is this a feature of the "registry" vs what is required by government in the local country. (Line 133)

**Authors: In order to avoid possible confusion, we have rephrased the sentence to: “As a first step, it is important to know how many records the database holds. This number should be readily available, and we have therefore analysed the ease of accessing it.”**

- - - - - - - - - - - - - - - - - -

7.23 why were these two subjects highlighted especially as they were not noteworthy or substantive. (Line 136)

**Authors: We have deleted these two features now.**

- - - - - - - - - - - - - - - - - -

7.24 is this a server issue; connectivity; (Line 140)

**Authors: We do not know the reason for the lag in loading RPCEC results. It seems to be at the RPCEC end, since other registries gave us no problem. In any case, we have deleted the entire feature now.**

- - - - - - - - - - - - - - - - - -

7.25 would think this is an important aspect to score; the logic to not score this as half of the registries did not contain is a shortcoming of the analysis as registries that do not have this attribute should receive a lower rating. Completion of variables is less about the registry vs the owner of the "data" (sponsor). (Line 140)

**Authors: We have now included in the Results and Discussion our analysis of whether the registry provides the reason for trial termination. However, the information pertaining to this feature is already present in the Extra fields. Hence we have not scored this features separately, since that would result in it being double counted.**

- - - - - - - - - - - - - - - - - -

7.26 why these attributes? Why is SSL important? (Line 154)

**Authors: We have now included a more detailed description of why SSL is important and why we have included this feature. [Lines 471–481 of the revised manuscript]**

- - - - - - - - - - - - - - - - - -

7.27 this is the sponsor's responsibility: QC (Line 166)

**Authors: In the Results and Discussion section we have now described why even though QC is the sponsor’s responsibility it does not always do this, and how it is the duty of the registry to facilitate higher quality registrations. [Lines 496–501 of the revised manuscript]**

- - - - - - - - - - - - - - - - - -

7.28 these points should have been provided in the introduction. (Line 181)

**Authors: We have now included these points in the Introduction.**

- - - - - - - - - - - - - - - - - -

7.29 there is no enough discussion on what has been done, why there is a gap and how this fills the gap in a meaningful way. (Line 189)

**Authors: We have rewritten the Introduction and have discussed the work done so far, and how our study fills a lacuna in the analysis and reporting of registries’ performance.**

- - - - - - - - - - - - - - - - - -

7.30 why is this ideal? (Line 195)

**Authors: As described in the manuscript, we propose an ‘interim ideal’ registry based on the features that we have assessed. That it is a limited goal on the way to achieving everything that ISCTR requires. And it is the ‘ideal’ from amongst the various options that one or more of the registries are already using.**

- - - - - - - - - - - - - - - - - -

7.31 seems per line 180 to 189 there have been other studies. not clear. (Line 215)

**Authors: We believe that the revised manuscript addresses this concern.**

- - - - - - - - - - - - - - - - - -

7.32 how was this impartial? (Line 216)

**Authors: Primarily based on the recommendations of ISCTR, we determined which features of the registries to assess. All scoring rationales were also based on the minimum standards outlined in the ISCTR, and recommendations from earlier studies in our literature survey. We believe that our scoring is impartial since this protocol rules out scoring that may be biased in favour of, or against, any particular registry.**

- - - - - - - - - - - - - - - - - -

7.33 over reach as this is a subjective statement; only state represents the authors' perspective not that of others. (Line 220)

**Authors: We have changed this sentence, which now reads “The scorecard above analyses features that are of interest to the authors and, by extension, possibly to other researchers concerned with the health of the trial ecosystem overall.”**

- - - - - - - - - - - - - - - - - -

7.34 not clear (Line 222)

**Authors: We have rephrased this to the following: “Other categories of users, such as medical professionals, patients, trial sponsors, policy makers, data scientists and so on, may wish to alter the assessed features, or the scoring, in order to rank the registries according to their priorities.”**

- - - - - - - - - - - - - - - - - -

7.35 registries cannot be interested in something; only those who work on registries (Line 224)

**Authors: We have rephrased the sentence to the following: “Further, the managers of other registries, either public or private, and either based on the data in the PR+ or not, may be interested in the results of this analysis.”**

- - - - - - - - - - - - - - - - - -

7.36 less about the registry and more about how users utilize unless all fields are required by the registry. (Line 230)

**Authors: We are not sure that we have understood this question. It is true that in no registry are all fields mandatory. Thus it would not be advisable to evaluate the quality of registration, and of information in a particular field, by comparisons across registries. However, as we have described in our response to Reviewer comment 7.13, we are only examining the *presence* of certain fields, not whether trialists have filled each of them.**

- - - - - - - - - - - - - - - - - -

7.37 need to write using English proper language fit for scientific publication (Line 238)

**Authors: We have rephrased the sentence to the following: “As noted above, 41% of the records are held in the other PR+, and they need to be examined as well.”**

- - - - - - - - - - - - - - - - - -

7.38 Define (Page 19, Box 1)

**Authors: We have described our assessment of this feature in greater detail in the revised manuscript.**

- - - - - - - - - - - - - - - - - -

7.39 more importantly why do registries include something not recommended by ISCTR? (Page 20, Box 1)

**Authors: We have added the following lines to the manuscript: “There may be a range of reasons for including these Extra fields. For instance, India had been criticized for the lack of appropriate oversight to ensure the ethical conduct of trials, and therefor CTRI asked trialists for details of the ethics committee even before ISCTR required this information [38,39]. Also, there have been demands from the Cochrane collaboration, and many other individuals and groups, to include several additional items in the ISCTR list, which WHO has not agreed to. It is alleged that the recommended list is closer to what industry demanded [40]. As such, although ISCTR may not list every field that many people believed to be essential, managers of particular registries may have chosen to list some of them.”**

- - - - - - - - - - - - - - - - - -

7.40 Restructure: what does WHO require; what do registries do; where is the difference and impact on value of registries. (Page 20, Box1)

**Authors: In each section of the Results and Discussion, we provide an introductory line, then the results, then the discussion. We have done this, incorporating the Reviewer’s points in this section.**

- - - - - - - - - - - - - - - - - -

7.41 is this an author term? It is a prospective trial even if data is only entered after the study starts. (Page 21, Box1)

**Authors: ‘Retrospective trials’ is used by other researchers – it is not our term. Nevertheless, earlier in the manuscript, we have added an explanatory line “Trials may be registered either prospectively or retrospectively, that is before the enrolment of the first participant or after.” Also, in order to avoid ambiguity, we have rephrased this sentence as follows: “Further, flagging retrospectively registered ones may shame the registrants into registering prospectively in future”.**

- - - - - - - - - - - - - - - - - -

7.42 what were the findings? (Page 21, Box1)

**Authors: We have revised the text to include the findings of the analysis.**

- - - - - - - - - - - - - - - - - -

7.43 how was 3 assigned out of 5: what does a value of 1 or 2 or 3 or 4 or 5 represent? (Page 8, Table 2a)

**Authors: As explained in the response to the Reviewer’s comment 5, we have moved Table 2b to the methods section (where it is now Table 1) to make the rationale of the scorecard available upfront. We hope this will alleviate the confusion regarding scoring.**

- - - - - - - - - - - - - - - - - -

7.44 11? 17? thought scores were 1 to 5. (Page 8, Table 2a)

**Authors: Please refer our response to the previous point (Reviewer’s comment 7.43)**

- - - - - - - - - - - - - - - - - -

8. The topic is interesting but the paper needs to be rewritten and the concept of a scorecard has to be rethought to ensure it has logical relevance to the reader, that the scoring is understandable and can be interpreted thereby leading to actionable insights.

**Authors: We have rewritten and reorganized the manuscript, and we hope that these changes address the reviewer’s concerns.**

- - - - - - - - - - - - - - - - - -

9. The paper has to be placed in context of other relevant studies completed to date.

**Authors: We have rewritten the Introduction to address this concern.**
