# Supplementary material for: A comparative analysis of important public clinical trial registries, and a proposal for an interim ideal one
Source: PLoS One. 2021 May 11;16(5):e0251191. doi: 10.1371/journal.pone.0251191 (PMC8112656; doi:10.1371/journal.pone.0251191)
Supplement: Attachment — Submitted filename: Response to reviewers.doc [file pone.0251191.s009.doc]

Dear Dr. Cox,

Please find below a response to each of the comments in the manuscript file, which we have numbered from 1–6 (including 4.1–4.11). In each case we have referenced (a) the line number in the original pdf, which the reviewer’s comment refers to and (b) the line number in the revised manuscript.

With regards,

Gayatri Saberwal

- - - - - - - - - - - - - - - - - -

1. Editor:

One reviewer also pointed out that having a unified registry is vey important in this era of Covid-19. I think that it would be worth discussing the importance of this work with respect to the Covid-19 epidemic.

**Authors:** We have addressed this in point 5.

- - - - - - - - - - - - - - - - - -

2. Reviewer #1:

One error in the edit (line 302) can be easily corrected.

**Authors:** Thank you for bringing this to our attention. We have rectified this error as follows [line 304 of the revised manuscript]:

ISCTR recommends that at the minimum, there must be a basic text search, **as well asand** it must be possible to searches within the interventions and conditions fields.

Changed to

ISCTR recommends that at the minimum, there must be a basic text search, **and** it must be possible to **conduct** searches within the interventions and conditions fields.

- - - - - - - - - - - - - - - - - -

3. Reviewer #2: Stopped reviewing content after a few pages; the content is too general, not descriptive enough; too many generalities are stated. The author needs to reconsider writing a laser-focused paper where the reader is taken step by step from purpose to approach to findings in a coherent, focused, understandable and 'supported by facts' approach.

**Authors:** We are sorry that the reviewer feels this way. We have done our best to answer his/her specific queries, listed below.

- - - - - - - - - - - - - - - - - -

4. Reviewer # 2’s comment bubbles in the manuscript.

4.1 why these 14 features? how were these 14 features from the total set? what is the total set?

(Line 39)

**Authors:** As described in the manuscript, we have compiled the list of 14 features based on a literature survey to understand the limitations of the registries [lines 129–133 of the revised manuscript]. However, we have evaluated these 14 features within the framework of the International Standards for Clinical Trial Registries (ISCTR) set forth by the WHO. As such, the total set of features can be taken as the set of standards described in ISCTR.

- - - - - - - - - - - - - - - - - -

4.2 not appropriate wording for a scientific paper. (Line 43)

**Authors:** Thank you for bringing this to our notice. We have rectified this error as follows [line 40–43 of the revised manuscript]:

The assessed features include the nature of the content; the number and nature of fields to conduct a search; data download formats; the nature of the audit trail; the health condition category; the documentation available on a registry website; and so on.

Changed to

The assessed features include the nature of the content; the number and nature of fields to conduct a search; data download formats; the nature of the audit trail; the health condition category; the documentation available on a registry website; etc.

- - - - - - - - - - - - - - - - - -

4.3 you are reporting scores without stating what was the scoring convention. (Line 44)

**Authors:** Thank you for this suggestion. We have now included, in the Abstract, a sentence about the scoring rationale, as follows:

“The registries received scores for their particular variation of a given feature based on a scoring rationale devised for each individual feature analysed.” [lines 43–45 of the revised manuscript]

- - - - - - - - - - - - - - - - - -

4.4 mere is not appropriate for scientific publications. (Line 50)

**Authors**: We have changed this [line 51–53 of the revised manuscript].

Even with this limited assessment, it is clear that some of the registries have much work to do, although a mere dozen improvements would significantly improve them.

Changed to

Even with this limited assessment, it is clear that some of the registries have much work to do, although even a few improvements would significantly improve them.

- - - - - - - - - - - - - - - - - -

4.5 word choice calls? not appropriate for a scientific publication. (Line 54)

**Authors:** The word ‘call’ is used quite routinely in the sense that we have used it. For example: “The first quantitative data demonstrating publication bias in clinical trials—and clear call for trial registries—was published in 1986”. (Goldacre B (2015) How to Get All Trials Reported: Audit, Better Data, and Individual Accountability. PLoS Med 12(4): e1001821. doi:10.1371/journal.pmed.1001821.) Therefore, we have not changed the word.

- - - - - - - - - - - - - - - - - -

4.6 why a battle? what is the supporting documentation of a battle? (Line 58)

**Authors:** For over 2 decades there have been efforts to try and get trialists to register their trials, and report their results on time. It has been described as a battle, such as in the following lines: “And does the registration battle foreshadow how current data transparency efforts—calls for companies to go beyond releasing summaries of trial results to releasing patient-level data from trials—will unfold?” Miller, J. E. (2013). (How a Clinical Trial Registry Became a Symbol of Misinformation. *[Hastings Center Report](http://onlinelibrary.wiley.com/doi/10.1002/hast.226/abstract#_blank)*, 43 (6), 11-12.) Therefore, we have not changed the word.

- - - - - - - - - - - - - - - - - -

4.7 conditions? do you mean disease areas or therapeutic areas? what is meant by an expanded access study? (Line 64)

**Authors:** We have modified this line and it now reads as [lines 63–66 of the revised manuscript]

...researchers have utilized the data in at least a dozen other ways, such as (i) analyzing the conditions, the medical interventions, the sponsors and so on of Expanded Access Studies registered in the United States (US) [5];

changed to

...researchers have utilized the data in at least a dozen other ways, such as (i) analyzing characteristics such as the disease areas, medical interventions, and sponsors of Expanded Access Studies registered in the United States (US) [5];

We have not changed or explained ‘Expanded Access Studies’, since this is a standard category of clinical studies permitted in the United States. An example is “Open-label, expanded access study of taliglucerase alfa in patients with Gaucher disease requiring enzyme replacement therapy. Kuter DJ, et al. *Blood Cells Mol Dis.* 2020 May;82:102418. doi: 10.1016/j.bcmd.2020.102418”

- - - - - - - - - - - - - - - - - -

4.8 contravention of the law? (Line 67)

**Authors:** In this instance, we are referring to cases where trials are conducted in violation of the law, as explained in reference 9.

- - - - - - - - - - - - - - - - - -

4.9 hidden duplicates? (Line 74)

**Authors:** The term hidden duplicates has been explained in references 15 and 16. It refers to a trial that is registered in two registries, but which cannot be unambiguously identified as duplicate entries of the same trial, because neither has listed the other’s ID in its ‘other study ID’ or ‘secondary ID’ field.

- - - - - - - - - - - - - - - - - -

4.10 Vague - why is the author making the statement? What advances? Writer needs to ground each statement in relevance of the argument being made with enough information for the reader to gain "usuable knowledge for the rest of the paper". (Line 75)

**Authors:** We have changed this [lines 76–79 of the revised manuscript].

Other studies have looked into the challenges faced, and advances made by individual registries [17,18].

changed to

Other studies have looked into the evolution of individual registries – the challenges faced, and advances made [17,18]. Such analyses have improved our understanding of clinical trial registries globally, and reinforce the efforts for creating uniform standards for trial registration globally.

- - - - - - - - - - - - - - - - - -

4.11 what scandals? (line 77)

**Authors:** We have changed this [lines 81–83 of the revised manuscript]:

Certain high profile scandals [19,20] resulted in numerous calls to increase transparency in clinical trials and to improve the public’s trust in the trials enterprise.

Changed to

Certain high profile scandals, such as the Vioxx case in 2004 and the Paxil case in 2015 [19,20], resulted in numerous calls to increase transparency in clinical trials and to improve the public’s trust in the trials enterprise.

- - - - - - - - - - - - - - - - - -

5. Reviewer #3

Interestingly in the era of Covid, the need for clinical trial registries that can be used for global clinical trials was not discussed.

**Authors**: We would like to clarify that although each registry discussed in this work has a well-defined remit in terms of what kinds of trials it hosts, all of them – as far as we can determine – are open to registering global trials, provided their own country or region is part of such trials. Perhaps the reviewer is referring to a unified registry, either for all conditions, or specifically for Covid-19 trials? We have added the following as lines 571–588 of the revised manuscript.

“A handful of the registries evaluated in this study have provided a customized search for Covid-19 trials on their home pages. Covid-19 knowledge hubs, that facilitate access to trials that are registered with existing registries such as those described in this work, have also been set up [53–55]. Undoubtedly, such efforts to catalogue all Covid-19 trials, and experiences with Covid-19, has contributed to a more rapid understanding of the virus, and a more efficient management of the pandemic.

This makes the case for (a) more of such knowledge hubs, or (b) a single world-wide trial registry. Arguments for a single world-wide registry have been made for many years [56]. However there are also counter-arguments, which highlight the fact that local registries (a) meet local ethics and regulatory requirements; (b) are easier to establish and monitor; (c) can be customized to local requirements; (d) are dependent on local infrastructure; (d) provide the national trial landscape and (e) can be a source of national pride [56,57].

Given that there are pros and cons to having a single world-wide registry, the best way forward may be to work to improve PR+ registries. If higher data quality and data sharing standards were more widespread, this would facilitate interoperability and data transfer while maintaining data integrity.

- - - - - - - - - - - - - - - - - -

6. While the discussion was good overall, the manuscript did lack detailed input from the different types of users as to the strengths and weaknesses of each of the registries reviewed. Such input would help identify the areas for improvement including how future registries need to support the wide range of individual users technical skills.

**Authors:** In the Discussion (line 544–550), while enumerating the various kinds of users of registry data, we have stated that “Registries have many users. The scorecard above analyses features that are of interest to the authors and, by extension, possibly to other researchers concerned with the health of the trial ecosystem overall. Other categories of users, such as medical professionals, patients, trial sponsors, policy makers, data scientists and so on, may wish to alter the assessed features, or the scoring, in order to rank the registries according to their priorities. For instance, a data scientist would be very appreciative of ANZCTR, which specifically enables web crawling of its records [49].”. We do not feel confident of creating different scoring systems. Ideally, this should be done by polling at least a few individuals in each category of users, and we would find it extremely challenging to do this in India. As such, any additional scoring system that we developed would be based on unvalidated assumptions, and would be unconvincing, even to us.
